# Exploring the role of octanol-water partition coefficient and Henry’s law constant in predicting the lipid-water partition coefficients of organic chemicals

**DOI:** 10.1038/s41598-022-19452-6

**Published:** 2022-09-02

**Authors:** Muhammad Irfan Khawar, Azhar Mahmood, Deedar Nabi

**Affiliations:** 1 Institute of Environmental Science and Engineering (IESE), School of Civil and Environmental Engineering (SCEE), National University of Sciences and Technology (NUST), Islamabad, H-12 Pakistan; 2 Environment and Agriculture Laboratory, School of Interdisciplinary Engineering and Sciences (SINES), National University of Sciences and Technology (NUST), Islamabad, H-12 Pakistan; 3 School of Natural Sciences (SNS), National University of Sciences and Technology (NUST), Islamabad, H-12 Pakistan

**Keywords:** Environmental chemistry, Cheminformatics, Environmental chemistry

## Abstract

Partition coefficients for storage lipid-water (logK_lw_) and phospholipid-water (logK_pw_) phases are key parameters to understand the bioaccumulation and toxicity of organic contaminants. However, the published experimental databases of these properties are dwarfs and current estimation approaches are cumbersome. Here, we present partition models that exploit the correlations of logK_lw_, and of logK_pw_ with the linear combinations of the octanol-water partition coefficient (logK_ow_) and the dimensionless Henry’s law constant (air–water partition coefficient, logK_aw_). The calibrated partition models successfully describe the variations in logK_lw_ data (n = 305, R^2^ = 0.971, root-mean-square-error (rmse) = 0.375), and in logK_pw_ data (n = 131, R^2^ = 0.953, rmse = 0.413). With the inputs of logK_ow_ and logK_aw_ estimated from the U.S. EPA’s EPI Suite, our models of logK_lw_ and logK_pw_ have exhibited rmse = 0.52 with respect to experimental values indicating suitability of these models for inclusion in the EPI Suite. Our models perform similar to or better than the previously reported models such as one parameter partition models, Abraham solvation models, and models based on quantum-chemical calculations. Taken together, our models are robust, easy-to-use, and provide insight into variations of logK_lw_ and logK_pw_ in terms of hydrophobicity and volatility trait of chemicals.

## Introduction

The lipid pool of an organism is predominantly comprised of storage lipids and membrane lipids^1^. Storage lipids are structurally triacylglycerides and constitute the main component of fat tissue. Membrane lipids exist in biological membranes and are mainly phospholipid in nature^2^. These two types are known to differ in their bioaccumulation capacities^3^. For toxicity assessment of chemicals and distribution of organic contaminants between living organisms and environmental media, storage lipid-water partition coefficient (logK_lw_) and phospholipid-water partition coefficient (logK_pw_) are important parameters^1,4^. The partitioning mechanisms of organic chemicals for these two types of lipids are different due to differences in their chemical structures and types of intermolecular interactions of these phases with contaminants^5^.

Experimental methods used to measure logK_lw_ and logK_pw_ are expensive, laborious, and time-consuming. Geisler and co-workers applied batch sorption experiments using headspace measurements to estimate the partitioning between the water phase and the storage lipid phases such as fish oil, linseed, olive, and goose fats^6^. Storage lipid-air partition coefficients (logK_la_) were measured for 80 chemicals using olive oil as the stationary phase in gas chromatography^7^. The logK_lw_ were then calculated using the thermodynamic cycle between logK_la_ and Henery’s Law Constant (HLC)^7^. In literature, different types of plant and animal storage lipids such as fish oil, olive oil, rapeseed oil, sunflower oil, seal oil and milk fat have been used to measure logK_lw_^6,8,9^. Silicone membrane samplers were successfully used to measure logK_lw_ for organochlorine pesticides (OCPs)^9^, polycyclic aromatic hydrocarbons (PAHs)^8^ and polychlorinated biphenyls (PCBs)^9^. Artificial lipid bilayers vesicles such as liposome have been extensively used to measure logK_pw_^10,11^. Methods such as ultracentrifugation, equilibrium dialysis, pH-metric titration, ultrafiltration, or third-phase (polymer, gas, or solvent)-mediated measurements were used to measure logK_pw_^5^. Endo and co-workers measured logK_pw_ for volatile and hydrophobic aliphatic chemicals using headspace sampling and solid phase dosing method, respectively^5^. However, these experimental methods are required to overcome the challenges such as ensuring the stable steady state concentrations, proper equilibrium time, mass balance consideration for all phases involved in the system, and reliable analytical quantification^8^. Consequently, there is a growing inclination towards reliable, robust, and fast estimation methods for the prediction of logK_lw_ and logK_pw_.

Estimation approaches based on one-parameter Linear Free Energy Relationship (op-LFER) models using octanol-water partition coefficient have been widely used to estimate storage lipid-water^2^ and phospholipid-water^5^ partition coefficients. Endo and co-workers reported R^2^ = 0.95 and rmse = 0.43 log unit with respect to experimental values of logK_pw_ for 156 neutral organic compounds^5^. The correlation of logK_lw_ with logK_ow_, which was estimated using KOWWIN module of U.S. Environmental Protection Agency’s Estimation Program Interface (EPI Suite)^12^, resulted in rmse = 0.61 log unit with respect to the experimental values of 305 chemicals^2^. Poly-parameter LFERs (pp-LFERs) based on Abraham solute descriptors (ASDs) have been found quite successful in predicting storage lipid-water^6^ and phospholipid-water^5^ partitioning properties. These ASDs include E (an indicator for polarizability), S (a depicter of a mix of polarity/polarizability), A and B (parameters for hydrogen bonding acidity and basicity, respectively), V (McGowan volume, as an indicator for cavity formation), and L (hexadecane − air partition coefficient accounting for dispersion interactions) descriptors. The reported rmse values were 0.20 log unit for storage lipid for a set of 247 chemicals, and 0.28 log unit for phospholipid for a set of 131 chemicals. Estimation methods based on quantum chemical calculations such as COSMOtherm and SPARC models^2,13^ exhibited rmse = 0.498 − 0.540 and 0.79 − 1.07 log units with respect to experimental values of logK_lw_ (n = 302 − 304) and of logK_pw_ (n = 207), respectively. However, these estimations methods suffer from a few theoretical and/or practical limitations. For instance, the op-LFERs are unable to account for all types of intermolecular interactions that diverse chemical families can experience during the partitioning process^1,14^. On the other hand, the available experimental database of all ASDs (E, S, A, B and L) for calibrated pp-LFERs is limited to about 3700 chemicals^15,16^. Though this database is gradually expanding, the experimental methods for the determination of ASM descriptors are challenging and require careful curation and considerations^17^. Additionally, there is redundancy in the information encoded in the ASDs, which can lead to inflated pp-LFERs if the calibration datasets are not carefully chosen^18^. Lastly, the methods based on quantum-chemical calculations are relatively sophisticated and require commercial software, which is not widely accessible to the users. Hence, there is a need to explore alternative estimation methods which overcome a number of these limitations in the existing approaches.

Recently, Naseem and coworkers demonstrated the importance of the inclusion of HLC in the formulation of two parameters LFER (tp-LFER) for the prediction of human skin permeation of neutral organic chemicals^19^. This study indicated that HLC is quantitatively more sensitive to specific intermolecular interactions such as dipole–dipole and hydrogen bonding interactions than logK_ow_, which significantly captures the nonspecific intermolecular interactions such as London dispersion forces. Thus, both descriptors complement each other by encompassing broad-spectrum intermolecular interactions in formulating the tp-LFER to describe the skin permeation of organic pollutants. Empirically speaking—besides these theoretical footings of tp-LFER, logK_ow_ enjoy wider experimental database of 13,700 chemicals^20,21^ and/or is easy to measure in the laboratory and/or is rapidly and reliably predictable^12,22^ than the ASDs. The experimental database of HLC is available for around two thousand chemicals^20,21^. However, it is difficult to measure the values of HLC in laboratory, but it can be rapidly predictable using Abraham solvation model’s equation and U.S, EPA’s EPI Suite. Thus, to capture all the specific and nonspecific intermolecular interactions, we decided to evaluate the role of logK_ow_ and logK_aw_ in combination to formulate tp-LFER. So, we systematically investigated the previously unexplored role of HLC in describing the partitioning variability for both types of lipids.

In the last, we comprehensively assessed the possible inclusion of our 2p-LFER models in the US Environmental protection agency’s Estimation Program Interface (EPI Suite) software which is a screening level tool and is being used to estimate several environmental properties and fate of chemicals. However, there is no module to predict logK_lw_ and logK_pw_ of organic chemicals. So, the integration of our models will enhance the capacity of this software. The objectives of this study are.

- To inspect the dimensionality and representativeness of datasets used to calibrate pp-LFERs and tp-LFERs models of logK_lw_ and logK_pw_.
- To develop and evaluate the performance of tp-LFERs models based on the linear combination of logK_ow_ and HLC for the prediction of logK_lw_ and logK_pw_.
- To assess the possible integration of newly developed models in EPI Suite software.

## Materials and methods

### Data source

To develop tp-LFER models, experimental values of logK_lw_ (n = 305, Table S1 in Supplementary material; SM) and logK_pw_ (n = 131, Table S2 in SM) were taken from literature^2,5^. In the published logK_lw_ dataset, the experimental values were measured at 37 °C for different types of lipids such as fish oil, linseed oil, goose fat, olive oil and milk fat. The fatty acid composition of these different types of lipids did not show any significant effect on the partitioning behavior^6^. Therefore, they were combined in a single dataset for calibration of tp-LFER. The logK_pw_ dataset comprised of the partition coefficients reported for liposomes (pure phosphatidylcholine or mixed with other lipid membranes) to water partitioning system. The experimental logK_pw_ values reported at a temperature ranging 20–40 °C were averaged due to nonsignificant variations found in their values^5^. The dataset represents different groups of chemicals like esters, ketones, alcohols, acids, alkanes, ethers, aldehydes, aromatic, and halogenated compounds with various substitutions.

HLC—which describes the partitioning tendency of organic pollutants between the air phase and water phase can be expressed as

1 $$ HLC = \frac{{P_{i} }}{{C_{w, i} }} $$

where *P*_*i*_ (in atm) and *C*_*w,i*_ (in mole/m^3^) respectively denote the partial pressure and molar concentration of chemical *i* in air phase and water phase. HLC values were made dimensionless using Eq. (2), which is also referred to as air–water partition coefficient (K_aw_).

2 $$ K_{aw} = \frac{HLC}{{RT}} $$

where *R* (8.205 × 10^–5^ m^3^. atm. K^−1^. mol^−1^) and *T* (298.15 K) are the molar gas constant and temperature.

To train the models for logK_lw_ and logK_pw_, the following three kinds of datasets were prepared based on logK_ow_ and logK_aw_. Initially, the values of logK_ow_ and logK_aw_ were calculated using respective Abraham Solvation Model equations^23–25^ from UFZ-LSER database calculator^16^ (dataset-I). Moreover, the experimental and estimated values of both logK_ow_ and logK_aw_ were also obtained from EPI Suite^21^. Here, we found 215 chemicals (Table S3 in SM) in logK_lw_ data and 93 chemicals (Table S4 in SM) in logK_pw_ dataset having the experimental values of both logK_ow_ and logK_aw_ (dataset-II). Similarly, the chemicals for which experimental logK_ow_, logK_aw_ or both were not available, their values were filled with estimated values from ASM equations (dataset-III, Tables S5 and S6 in SM). We used all these datasets (I, II and III) to develop tp-LFER models equations. Here, dataset-I depicts purely estimated values of base parameters (logK_ow_ and logK_aw_), dataset-II shows purely experimental values while dataset-III contains the mix of experimental and estimated values of logK_ow_ and logK_aw._

Furthermore, estimated values from EPI Suite for logK_ow_ and logK_aw_ were used as an input parameter in newly developed tp-LFER models (Tables S7 and S8 in SM) to find out the suitability of our models to be integrated in EPI Suite software as a new module. Comparison of different existing models with newly developed tp-LFER models can also be viewed (Table S9 in SM).

### Data analyses

All statistical analyses were performed using R statistical environment (version—4.0.3)^26^ and XLSTAT 2020^27^. Principal component analysis (PCA) was used to dissect the intermolecular interactions information encoded in ASDs and their correspondence with logK_ow_ and logK_aw_ obtained directly from UFZ-LSER database. Pearson correlation analysis was used to investigate the overlap in information among different variables used to develop these models. The linear relationship between two continuous random variables, as indicated by the Pearson correlation coefficient (*r*), is monotonic in nature^28^. Though quite arbitrary in nature, a general rule of thumb was followed in this study, which classifies the pairwise correlation between variables as negligible, weak, strong, and very strong relationship if the value of *r* respectively falls in the range of 0.00–0.010, 0.10–0.39, 0.40–0.69, and 0.90–1.00^28^.

For the development of two parameters models, dependent variables, logK_lw_ and logK_pw_, were regressed against independent variables, logK_ow_ and logK_aw_, using multiple linear regression. To delineate the applicability domains of all the tp-LFERs models, influence plots were used, which helps visualize the studentized residuals, hat-values, and Cook’s distance values simultaneously. Leverages higher than the critical values generally indicate possible issues with predictor variables, which in this case are logK_ow_ and logK_aw_. The values of studentized residual greater than the reference values indicate a possible problem in the measured value of the independent variables.

### Validation of the tp-LFER models

Cross-validation tests such as leave-one-out, k-fold (k = 10, repeat = 0 and 3), and bootstrapping 1000 resamples were performed to assess the internal validation, robustness, and predictive capability of each model (Sect. 1 in SM). For external validation, the complete dataset of logK_lw_ (Table S1 in SM) was split randomly into a training set (n_training_ = 245, Table S10in SM) and a validation set (n_validation_ = 60, Table S11in SM). Similarly, logK_pw_ dataset (Table S2 in SM) was split randomly into a training set (n_training_ = 107, Table S12 in SM) and a validation set (n_validation_ = 24, Table S13in SM).

The performance of tp-LFER of logK_lw_ was further evaluated using an independent dataset (henceforth called the test set) from the literature^29^ (n_test_ = 18, Table S14in SM), in which lipid (ultra-pure triolein)-water partition coefficients were measured for alkyl benzene, halogenated benzene, short-chain chlorinated hydrocarbons, organochlorine pesticides, polychlorinated biphenyl and polycyclic aromatic hydrocarbons (Sect. 5a in SM). Similarly, an independent test set of logK_pw_ values (n_test_ = 36, Table S15 in SM) was taken from the literature^30–38^ to validate the predictive power of the tp-LFER model. In this dataset, liposome-water partition coefficients were measured for neutral organic compounds (Sect. 5b in SM). However, these are non or weakly polar compounds thus too biased to evaluate the general predictive power of the developed models.

## Results and discussion

### Justification of two parameters LFER (tp-LFER) models

To evaluate the principle of parsimony for pp-LFERs reported for logK_lw_ and logK_pw_, dimensionality analyses were performed on their calibration datasets comprising of ASDs (Sect. 2 in SM). The aim was to know how many independent dimensions of information are required to explain the total variance coded in ASDs for these datasets. The PCA tests performed on a set of ASDs indicate that the first two dimensions represent 75.7% of the information for the logK_lw_ LFER dataset and 79.1% for the logK_pw_ LFER dataset (Sect. 2 in SM). This was expected as there is a considerable overlap in information among ASDs^39^, which warrants a careful selection of calibration dataset to avoid inflation in the fitted coefficients of ASM equations^18^.

**Figure 1:**
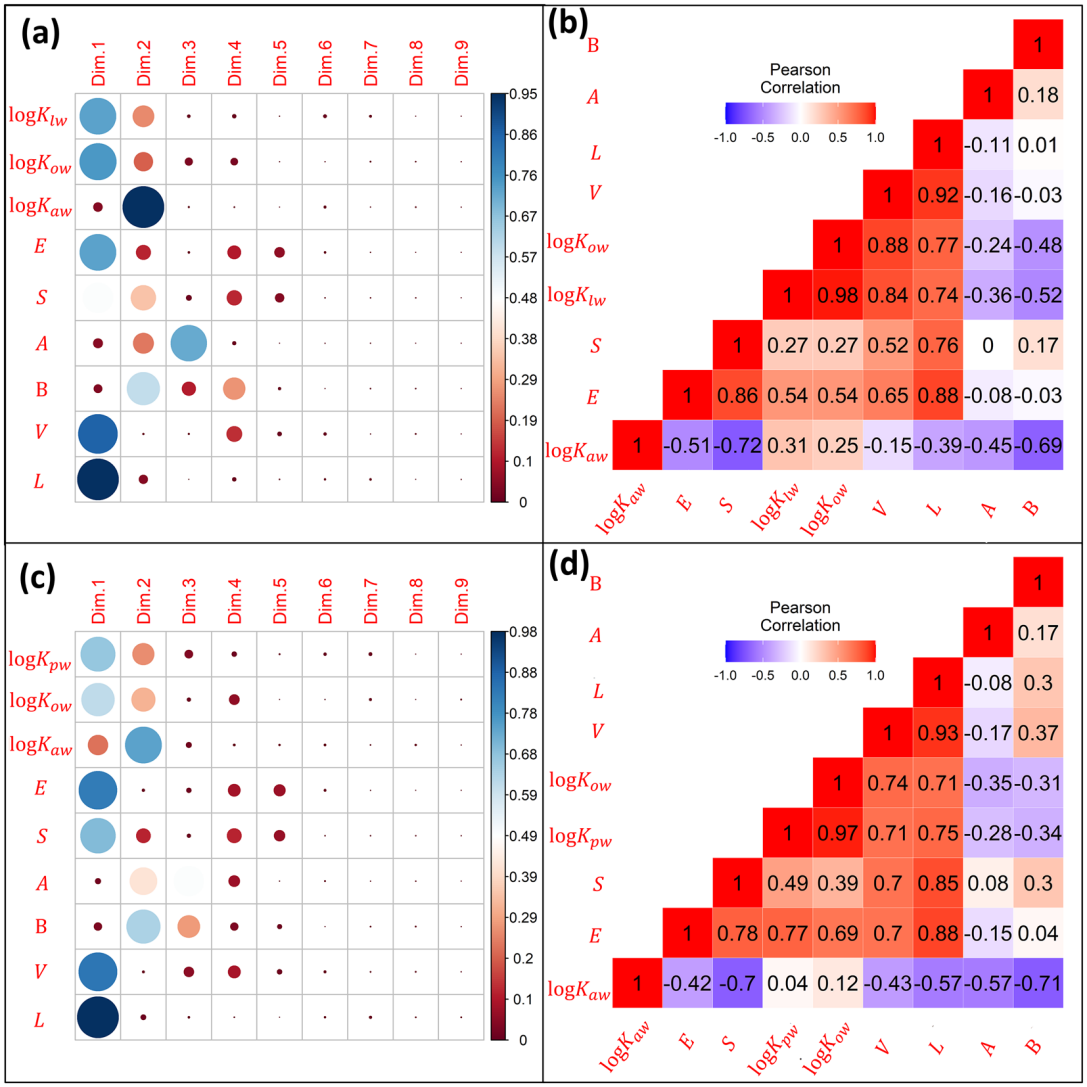
Dimensionality analyses on the calibration datasets for tp-LFER models of logK_lw_ and logK_pw_. The upper panels show the results obtained by **(a)** the Principal Component Analysis (PCA) and **(b)** Pearson Correlation Analysis performed on 305 × 9 matrix, [logK_lw_, E, S, A, B, V, L, logK_ow_, logK_aw_]. The lower panels show the results of (**c**) PCA and (**d**) Pearson Correlation Analysis on 131 × 9 matrix, [logK_pw_, E, S, A, B, V, L, logK_ow_, logK_aw_]. For left panels (**a**) and (**c**), the color intensity and size of the circle are proportional to the quality of presentation of a variable in each principal dimension (dim). For panels (**b**) and (**d**): each square contains the value of correlation coefficient for each pair of variables. Blue and red colors show negative and positive correlations between the pairs, respectively.

To investigate the correspondence of logK_ow_ and logK_aw_ with other descriptors, PCA was performed on ASDs along with logK_ow_, logK_aw_, logK_lw_, and logK_pw_ for all the datasets (Tables S1 and S2 in SM) used to calibrate the ASM equations for storage lipid-water and phospholipid-water partitioning properties. A PCA analysis on 305 × 9 matrix, [logK_lw_, E, S, A, B, V, L, logK_ow_ and logK_aw_], indicates that the logK_lw_ mainly contributes to the first 2 of 9 dimensions (Fig. 1a). The major contribution of logK_ow_ and logK_aw_ is partitioned into the first two dimensions indicating that they would significantly account for the variance in logK_lw_. Moreover, the non-specific ASDs (E, V and L) are dominantly contributing to the first dimension. The specific ASDs (S, A and B) show their presence from second to onward dimensions. These correspondences are further corroborated in the correlogram depicting the Pearson correlation (Fig. 1b). There is a strong correlation between logK_lw_ and logK_ow_ (*r* = 0.98), while a moderate correlation is found between logK_lw_ and logK_aw_ (*r* = 0.31).

The PCA on 131 × 9 matrix, [logK_pw_, E, S, A, B, V, L, logK_ow_ and logK_aw_], led to the partitioning of logK_pw_ principally in the first two dimensions with a negligible contribution in the remaining seven dimensions (Fig. 1c). The behavior of logK_ow_ is like that of logK_pw_ in terms of its distribution in PCA. Both logK_ow_ and logK_aw_ are primarily partitioned in the first two dimensions. There is a strong correlation between logK_pw_ and logK_ow_ (*r* = 0.97) (Fig. 1d)**.** However, the pairwise correlation between logK_pw_ and logK_aw_ (*r* = 0.04) appears to be weak, which indicates that the information coded by logK_aw_ alone is relatively lower than by logK_ow_ to estimate logK_pw_ for this particular dataset. However, the role of logK_aw_ is statistically significant when evaluated as a linear combination of logK_ow_ and logK_aw_ to describe the partitioning variability in logK_pw_ data. Correlations of logK_lw_ with hydrogen bonding interaction parameters A and B (*r* = 0.36 and − 0.52) are more negative than the correlations observed between logK_pw_ with A and B (*r* = − 0.28 and − 0.34). The correlation of logK_lw_ with the polarity/polarizability descriptor, S, is relatively weaker (*r* = 0.27) than the one observed for logK_pw_ and S (*r* = 0.49). Similarly, the correlation of logK_lw_ with the descriptor of cavity formation V (*r* = 0.84) is higher than with logK_pw_ (*r* = 0.71). This indicates that the phospholipids are slightly more polar in nature than storage lipids. This is further corroborated by pp-LFER equations for these two types of lipids. The magnitudes of system coefficients for the polar descriptors of the storage lipid-water system are smaller than those for the phospholipid-water system.

### Two parameters LFER (tp-LFER) models

This section reports the results of tp-LFER models developed on datasets I, II and III (detail has been given in subheading 2.1), with the input of logK_ow_ and logK_aw_ for the estimation of logK_lw_ and logK_pw_ of organic chemicals.

#### Storage Lipid-water tp-LFER model (dataset-I)

The tp-LFER model based on a relationship of logK_lw_ with a linear combination of logK_ow_ and logK_aw_, resulted in the following model equation (Eq. 3) for the logK_lw_ dataset.

3 $$ \begin{aligned} & logK_{lw} = { } - 0.236{ }\left( { \pm { }0.043} \right) + 1.102{ }\left( { \pm { }0.016} \right){ }logK_{ow} + 0.069{ }\left( { \pm { }0.01} \right){ }logK_{aw} { } \\ & {\text{n}}\, = \,{3}0{5}, \quad {\text{R2}}\, = \,0.{971}, \quad {\text{Adj}}.{\text{ R}}^{{2}} \, = \,0.{97}0, \quad {\text{rmse}}\, = \,0.{375},\quad {\text{F}}\,{\text{ statistics}}\, = \,{5}0{46} \\ \end{aligned} $$

here the value in parentheses depicts the standard error around the mean value of fitting coefficients obtained by bootstrap resampling. n denotes the number of experimental values of logK_lw_, R^2^ shows the coefficient of determination, Adj. R^2^ denotes the adjusted coefficient of determination, rmse and F statistics denote root-mean-squared-error and Fisher statistics respectively.

In Eq. (3), the role of logK_ow_ is stronger by one order of magnitude than that of logK_aw_ in explaining the variations of logK_lw_. This is expected as octanol is a good surrogate phase for lipids. However, by excluding the logK_aw_ from this equation, the accuracy of the model reduces by 0.024 log units (Sect. 4a in SM). Although this improvement in terms of the overall rmse of our model is fractional compared to op-LFER, but the rmse value reflects an error for the whole model that averages out the large and small residuals observed for example for influential observations, polar, nonpolar, and hydrophobic chemicals. In the case of the polar chemicals that depict significant hydrogen bonding interaction traits, the role of logK_aw_ in our two-parameter model (tp-LFER) generally becomes statistically and numerically significant. For example, for organochlorine pesticides such as lindane, dieldrin, heptachlor, chlordane, and p,p’-DDE (taken from the test set, Table 14 in SM), the departure of the predicted values from the experimental values can be doubled if logK_aw_ is ignored (i.e., if op-LFER is used to predict the values for these chemicals). The values of absolute residuals as a function of Abraham solute parameter B, for organochlorine pesticides obtained for both models (i.e., op-LFER and tp-LFER) can be viewed (Fig. S1 in SM). Here, we present another example of substituted benzenes: toluene and phenol from Table S1 of SM. Substituting a non-polar methyl group of toluene with a polar group such as OH makes toluene a strong bipolar molecule with strong hydrogen bonding interaction. Ignoring HLC—which shows strong correlations as depicted by the Pearson correlation of A, B, and S parameters with the HLC (Fig. 1b)—in formulating LFER significantly inflates the residuals for the phenol as compared to the toluene. To further corroborate the better performance of tp-LFER for polar chemicals, we used a subset of polar chemicals (having non-zero values of A and B parameters) for model training, which exhibited pronounced inferior statistics for op-LFER (R^2^ = 0.823, rmse = 0.510) compared to tp-LFER (R^2^ = 0.878, rmse = 0.426). (Sect. 4 in SM).

Comparatively, the pp-LFER based on ASDs exhibited slightly better statistics (n = 247, R^2^ = 0.977 − 0.988, rmse = 0.20 − 0.29) than those observed for Eq. (3). However, the experimental values of ASDs are not as frequently available as are the values for logK_ow_. Previously, a quantitative structure-property relationship (QSPR) model of logK_lw_^13^, based on quantum-chemical descriptors and octanol-water partitioning coefficient, exhibited rmse = 0.468 and R^2^ = 0.955. Compared to this QSPR model, our tp-LFER performed better by yielding (rmse = 0.375 and R^2^ = 0.971) for predicting storage lipid-water partition coefficients. However, the QSPR model is computationally expensive and requires commercial software, which is not the case for our model.

Moreover, four types of cross-validation tests (leave-one-out, k-fold (k = 10), repeated K-fold (3 times), and bootstrapping with 1000 resamples) performed on logK_lw_ dataset exhibited rmse values in a range of 0.369 − 0.378 and R^2^ values spanning 0.970 − 0.971 (Sect. 1 in SM), which are in close agreement with the regression statistics of Eq. (3). During external validation, Eq. (4) was obtained by calibrating tp-LFER on the training set (n_training_ = 245). The values of logK_lw_ for the validation set (n_validation_ = 60) and the test set (n_test_ = 18) were predicted using Eq. (4). These predicted values were then compared with the experimental values to calculate R^2^_validation_, rmse_validation_, R^2^_test_ and rmse_test_.

4 $$ \begin{aligned} & logK_{lw} = - 0.210 \left( { \pm 0.048} \right) + 1.102 \left( { \pm 0.013} \right) logK_{ow} + 0.078 \left( { \pm 0.012} \right) logK_{aw} \\ & \begin{array}{*{20}l} {{\text{n}}_{{{\text{training}}}} = { 245},} \hfill & {{\text{R}}^{{2}} = \, 0.{97}0,\quad {\text{ Adj}}.{\text{ R}}^{{2}} = \, 0.{97}0,} \hfill & {{\text{rmse }} = \, 0.{381},\quad {\text{ F statistics }} = { 3977}} \hfill \\ {{\text{n}}_{{{\text{validation}}}} = { 6}0,} \hfill & {{\text{R}}^{{2}}_{{{\text{validation}}}} = \, 0.{963},} \hfill & {{\text{rmse}}_{{{\text{validation}}}} = \, 0.{434}} \hfill \\ {{\text{n}}_{{{\text{test}}}} = { 18},} \hfill & {{\text{R}}^{{2}}_{{{\text{test}}}} = \, 0.{952},} \hfill & {{\text{rmse}}_{{{\text{test}}}} = \, 0.{358}} \hfill \\ \end{array} \, \\ \end{aligned} $$

As depicted by the R^2^_validation_, rmse_validation_, R^2^_test_ and rmse_test_, Eq. (4) reliably estimated the values of logK_lw_ for the external datasets. Moreover, the values of fitting coefficients in Eq. (3) are statistically similar to those in Eq. (4). Furthermore, regression statistics of Eq. (3) are in close agreement with regression statistics obtained for Eq. (4).

#### Phospholipid-water tp-LFER model (dataset-I)

The tp-LFER, which is trained on a linear combination of logK_ow_ and logK_aw_, successfully described the variation in logK_pw_ data via Eq. (5).

5 $$ \begin{aligned} & logK_{pw} = - 0.247\left( { \pm 0.095} \right) + 1.070\left( { \pm 0.021} \right)logK_{ow} - 0.056\left( { \pm 0.013} \right)logK_{aw} \\ & {\text{n }} = { 131},\quad {\text{R}}^{{2}} = \, 0.{953},\quad {\text{Adj}}.{\text{ R}}^{{2}} = \, 0.{952},\quad {\text{rmse }} = \, 0.{414},\quad {\text{F statistics }} = { 1293} \\ \end{aligned} $$

In Eq. (5), the influence of logK_ow_ variable is about an order of magnitude higher as compared to logK_aw_ variable. However, if the role of logK_aw_—which is statistically significant in Eq. (5)—is ignored in formulating the LFER, the model accuracy reduces by 0.027 log unit (Sect. 4b in SM). Chemicals with a higher logK_ow_ value tend to have a higher logK_pw_ value. On the other hand, a chemical having a higher logK_aw_ would have a lesser logK_pw_ value. The influence of logK_aw_, as indicated by relative values of fitting coefficient of logK_aw_ in Eqs. (3) and (5)—is slightly more pronounced in describing the variations in logK_lw_ than in logK_pw_**.** However, the role of logK_ow_ in describing the partitioning variability for both phases is almost similar. As indicated by (±) signs of fitting coefficient of logK_aw_ in Eqs. (3) and (5), the increase in logK_aw_ value of chemical slightly increases its logK_lw_ value but decreases its logK_pw_ value. This may be attributed to the slightly more polar nature of phospholipids compared to storage lipids. Hence, the fugacity (escape potential) difference experienced by the chemicals between the phospholipid and water is not as strong as in the storage lipid and water system. Being a descriptor of polar interactions, logK_aw_ favors the partitioning of chemicals with relatively higher solubility and less volatility towards a polar phase. This is further substantiated by our dimensionality analysis of ASDs along with logK_ow_ and logK_aw_ (Fig. 1a). The air–water system is more sensitive to polar interactions (Fig. 1b: *r* = − 0.72, − 0.45, − 0.69 for correlations between logK_aw_ and S, A, and B, respectively) compared to the octanol–water system (Fig. 1b: *r* = 0.27, − 0.24, − 0.48 for correlations of logK_ow_ with S, A, and B respectively). This is further corroborated by the respective pp-LFER equations for these two types of lipids, where the fitting coefficients of non-specific ASDs are higher in magnitude for logK_lw_ than the ones for logK_pw_. On the other hand, the fitting coefficients of specific ASDs in these ASM equations are lesser in magnitude for logK_lw_ than the ones for logK_pw_.

However, cross-validation of Eq. (5) indicates that the model is robust for the predictive purpose. The values of rmse (0.412 − 0.422) and R^2^ (0.948 − 0.951) obtained from the leave-one-out test, k-fold test (k = 10, repeat = 0 and 3), and bootstrapping test (1000 resamples) (Sect. 1 in SM) were not only internally consistent but were in close agreement with the values of rmse and R^2^ obtained for Eq. (5). The strong predictive power of tp-LFER model of logK_pw_ is further corroborated by the following external validation test. First, Eq. (6) was obtained by fitting tp-LFER model of logK_pw_ on the training set (n_training_ = 107). Second, Eq. (6) was used to make predictions for the validation set (n_validation_ = 24) and the test set (n_test_ = 36).

6 $$ \begin{aligned} & logK_{pw} = { } - 0.234{ }\left( { \pm { }0.108} \right) + 1.067{ }\left( { \pm { }0.024} \right){ }logK_{ow} - 0.049{ }\left( { \pm { }0.014} \right){ }logK_{aw} { } \\ & \begin{array}{*{20}l} {{\text{n}}_{{{\text{training}}}} = { 1}0{7},} \hfill & {{\text{R}}^{{2}} = \, 0.{95}0, \quad {\text{Adj}}.{\text{ R}}^{{2}} = \, 0.{949},} \hfill & {{\text{rmse }} = \, 0.{423}, \quad {\text{F statistics }} = { 99}0} \hfill \\ {{\text{n}}_{{{\text{validation}}}} = { 24},} \hfill & {{\text{R}}^{{2}}_{{{\text{validation}}}} = \, 0.{967},} \hfill & {{\text{rmse}}_{{{\text{validation}}}} = \, 0.{4}0{2}} \hfill \\ {{\text{n}}_{{{\text{test}}}} = { 36},} \hfill & {{\text{R}}^{{2}}_{{{\text{test}}}} = \, 0.{613},} \hfill & {{\text{rmse}}_{{{\text{test}}}} = \, 0.{6}0} \hfill \\ \end{array} \\ \end{aligned} $$

The predicted values were compared favorably with the experimental values for the validation set. However, for the test set the predictive performance was low, which may be attributed to the fact that this dataset contains complex molecules having multiple ionizable functional groups such as drugs. For instance, predicted values of logK_pw_ for warfarin, quinine, and 2,4,6-trimethylaniline deviated by more than one log unit with respect to their experimental values. These huge deviations may be attributed to the quality of experimental data used for the comparison with the prediction values. For example, there is about two order of magnitude difference observed in the measured values of logK_pw_ for the neutral and ionized warfarin^30^.

#### Two parameters (tp-) LFER models (dataset-II)

Here, we developed tp-LFER models with the input of purely experimental values of base parameters (logK_ow_ and logK_aw_). For the estimation of storage lipid-water (logK_lw_) partition coefficient, the model was trained on 215 chemicals (Table S3 in SM). The following equation was developed.

7 $$ \begin{aligned} & logK_{lw} = { } - 0.064{ }\left( { \pm { }0.055} \right) + 1.049{ }\left( { \pm { }0.014} \right){ }logK_{ow} + 0.121{ }\left( { \pm { }0.014} \right){ }logK_{aw} \\ & {\text{n }} = { 215},\quad {\text{R}}^{{2}} = \, 0.{97}0, \quad {\text{Adj}}.{\text{ R}}^{{2}} = \, 0.{969},\quad {\text{rmse }} = \, 0.{388},\quad {\text{F statistics }} = { 3299} \\ \end{aligned} $$

Similarly, for phospholipids-water partition coefficient (logK_pw_), the following model equation was developed for 93 chemicals (Table S4 in SM).

8 $$ \begin{aligned} & logK_{pw} = - 0.401\left( { \pm 0.0109} \right) + 1.070\left( { \pm 0.022} \right)logK_{ow} - 0.111\left( { \pm 0.021} \right)logK_{aw} \\ & {\text{n }} = { 93},\quad {\text{R}}^{{2}} = \, 0.{963}, \quad {\text{Adj}}.{\text{ R}}^{{2}} = \, 0.{963}, \quad {\text{rmse }} = \, 0.{353},\quad {\text{F statistics }} = { 1184} \\ \end{aligned} $$

#### Two parameters (tp-) LFER models (dataset-III)

Two parameters LFER models were also developed using dataset-III in which experimental values of logK_ow_ and logK_aw_ were taken and the missing values were filled with ASM estimated values. The following equation was developed for the estimation of logK_lw_.

9 $$ \begin{aligned} & logK_{lw} = { } - 0.128{ }\left( { \pm { }0.045} \right) + 1.064{ }\left( { \pm { }0.012} \right){ }logK_{ow} + 0.110{ }\left( { \pm { }0.011} \right){ }logK_{aw} \\ & {\text{n }} = { 3}0{5}, \quad {\text{R}}^{{2}} = \, 0.{969},\quad {\text{Adj}}.{\text{ R}}^{{2}} = \, 0.{969},\quad {\text{rmse }} = \, 0.{385}, \quad {\text{F statistics }} = { 4774} \\ \end{aligned} $$

Similarly, for logK_pw_ the following model equation was developed.

10 $$ \begin{aligned} & logK_{pw} = - 0.186\left( { \pm 0.101} \right) + 1.059\left( { \pm 0.022} \right)logK_{ow} - 0.040\left( { \pm 0.014} \right)logK_{aw} \\ & {\text{n }} = { 131}, \quad {\text{R}}^{{2}} = \, 0.{948},\quad {\text{Adj}}.{\text{ R}}^{{2}} = \, 0.{947},\quad {\text{rmse }} = \, 0.{434}, \quad {\text{F statistics }} = { 1168} \\ \end{aligned} $$

Observing the statistics of Eqs. (3), (4), (7), and (9) developed for logK_lw_, we noticed similar results in context of R^2^, Adj. R^2^ and rmse values. The same fashion was observed for Eqs. (5), (6), (8), and (10) of logK_pw_. It indicates that the models are well performing and robust. However, we recommend users to use Eqs. (3) or (9) and (5) or (10) for predicting logK_lw_ and logK_pw_ respectively as these were developed on large data size comparatively.

## Application domain

**Figure 2:**
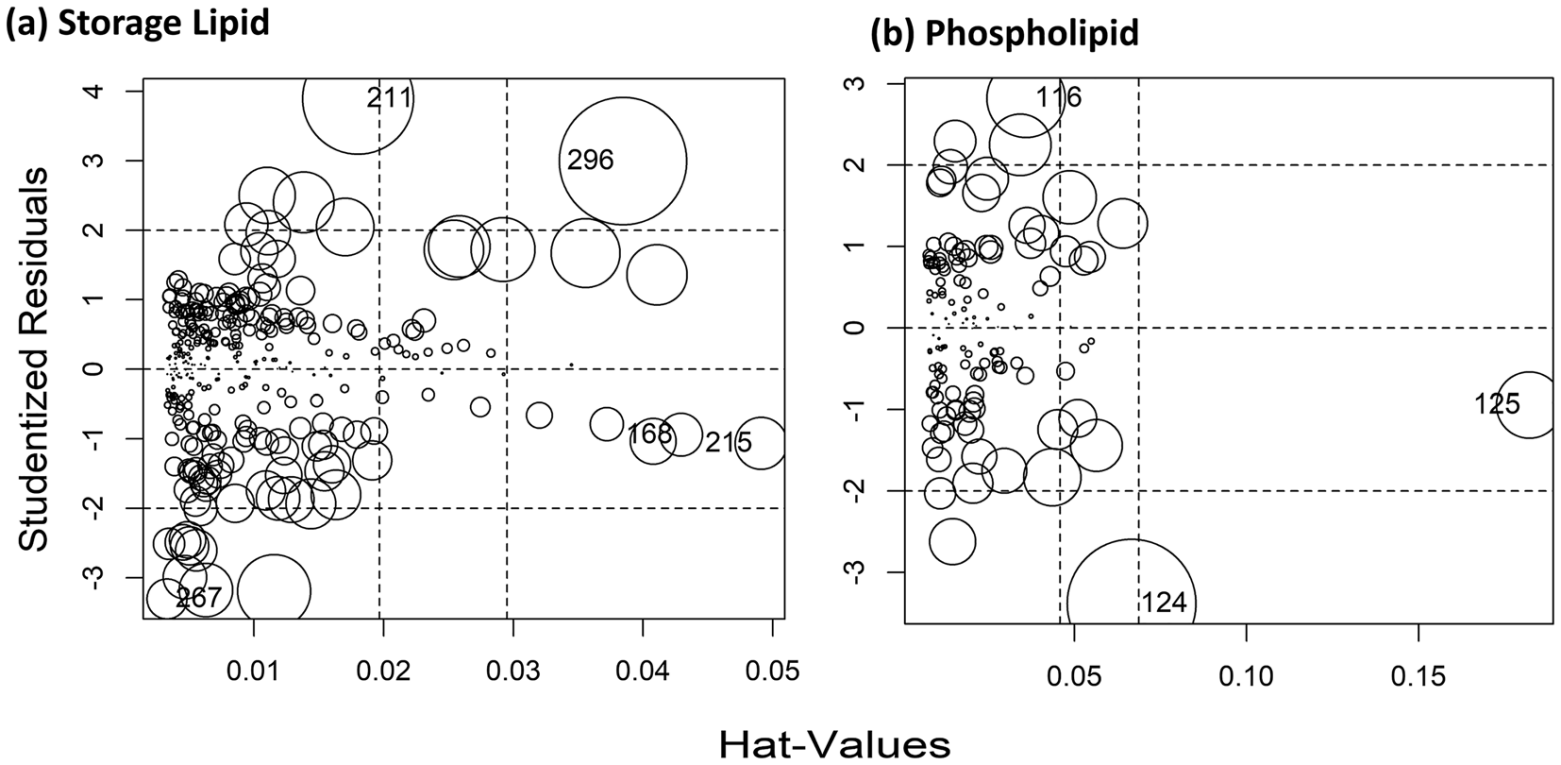
The application domain of tp-LFER models as evaluated by the plot of studentized residuals versus hat-values along with the Cook’s distance (which are proportional to circle size) for (**a**) storage lipid-water system and (**b**) phospholipid-water system. In panel **(a)**, observation numbers 168, 211, 215, 267, and 296—flagged as influential due to higher value than the critical values of either studentized residual or hat or Cook’s distance—correspond to pentadecane, 2,4-dinitrotoluene, hexadecane, 2,2,3,3,4,4,4-heptafluoro-1-butanol, and benzo[a]pyrene, respectively. In panel **(b)**, observation numbers 116, 124, and 125, which are flagged as influential, correspond to 3,4-dinitrophenol, estradiol, and estriol, respectively.

To ascertain the application domain for these developed models, influence plots were prepared (Fig. 2). The influence plot shows that most of the chemicals fall in the application domain of the models. However, the following 5 chemicals were flagged as influential observations for tp-LFER model of logK_lw_: 2,2,3,3,4,4,4-heptafluoro-1-butanol, pentadecane, 2,4-dinitrotoluene, hexadecane and benzo[a]pyrene. Values greater than the critical hat values for these chemicals indicate a likely issue with their measured value of logK_lw_. The values of logK_ow_ and logK_aw_ for very hydrophobic and fluorinated compounds might be in considerable error^17^. Higher than the critical studentized residual value of 2,4-dinitrotoluene indicates the possible problem with its measured value of logK_lw_ as it is very hydrophilic (logK_aw_ = − 5.88). While benzo[a]pyrene, pentadecane and hexadecane are very hydrophobic (logK_ow_ = 5.78, 8.8 and 9.3, respectively) in nature (Sect. 3 in SM).

For logK_pw_ tp-LFER model, the following 3 chemicals were flagged as influential based on their studentized residuals and hat values: 3,4-dinitrophenol, estradiol and estriol. All these 3 chemicals are very hydrophilic in nature (logK_aw_ = − 9.02, − 11.31 and − 17.17) respectively. Ensuring mass balance for such chemicals is quite challenging during the measurement due to their ultra-low accumulations in the lipid phase. Our models work within the confines of application domains of logK_ow_ and logK_aw_ estimation methods which are reported in the documentation of EPI Suite^12^ and UFZ-LSER database^21^. Our models are very suitable to deal with neutral organic compounds. The nature of influential chemicals of the current study highlighted the limitations of these developed models that there might have predicted errors for the compounds of very hydrophilic, very hydrophobic, and strong hydrogen bonds (H-bond) donor nature.

## Evaluation of tp-LFER models for possible inclusion in EPI Suite

EPI Suite is a screening-level tool, which comprises 14 modules, that helps estimate several environmental properties. However, there is no module to predict logK_lw_ and logK_pw_ in EPI Suite. The tp-LFER models developed in this study for the estimation of logK_lw_ and logK_pw_ were evaluated for possible inclusion in EPI Suite. For this purpose, we first evaluated the quality of the input parameters of tp-LFERs, logK_ow_ and logK_aw_, obtained from EPI Suite by comparing its predictions to the available experimental values in the main calibration datasets of logK_lw_ and logK_pw_. In this comparison, we also included the predictions of logK_ow_ and logK_aw_ retrieved by respective ASM equations. EPI Suite performed similarly to ASM in predicting the values of logK_ow_ and logK_aw_. Comparison of the predicted values of logK_ow_ obtained from EPI Suite and ASM with 304 experimental values of logK_ow_ resulted in rmse = 0.28 and 0.26, respectively. For logK_aw_, the comparisons of predicted values from EPI Suite and ASM equation with 296 experimental values exhibited rmse = 0.50 log unit for both models. Next, we inputted the EPI Suite estimated values of logK_ow_ and logK_aw_ in tp-LFER model equations for logK_lw_ (Table S7 in SM) and logK_pw_ (Table S8 in SM), which revealed rmse = 0.52 respectively for both models with respect to their experimental values. These comparisons imply that the estimated values of logK_ow_ and logK_aw_ from EPI Suite are of acceptable quality for the potential use of our tp-LFERs as EPI Suite modules.

## Conclusions

In this study, we have successfully demonstrated that the two parameters LFER (tp-LFER) model perform similar to parameter intensive Abraham solvation models for the prediction of logK_lw_ and logK_pw_. Comparatively, our models are easy-to-use and perform better than the recently reported QSPR based model for the estimation of lipid-water (logK_lw_) partition coefficients. These tp-LFER models can be used as an alternative estimation approach where the users do not have access to commercial software or experimental Abraham solute descriptors and reliable logK_ow_ and HLC data are available. The proposed models can be integrated within EPI Suite because the values of logK_ow_ and logK_aw_ can easily be obtained by respective modules of EPI Suite. Moreover, our models shed light on the partitioning behavior of neutral organic pollutants in terms of their hydrophobicity and volatility. These models can also be used for the risk assessment of organic chemicals.

## Supplemental material (SM)

Supplementary material contains; the list of chemicals used to train tp-LFER models with their values of logK_lw_ and logK_pw_ partition coefficients and logK_ow_ and logK_aw_, Cross validation, diagrams of dimensionality analyses and lists of flagged chemicals.

## Supplementary Information

Supplementary Information.

## Data Availability

All data generated or analyzed during this study are included in this published article and its supplementary material file.
