# Supplementary material for: Exploring the role of octanol-water partition coefficient and Henry’s law constant in predicting the lipid-water partition coefficients of organic chemicals
Source: Sci Rep. 2022 Sep 2;12:14936. doi: 10.1038/s41598-022-19452-6 (PMC9440013; doi:10.1038/s41598-022-19452-6)
Supplement: Supplementary file 1 — Supplementary Information. [file 41598_2022_19452_MOESM1_ESM.docx]

“SUPPLEMENTARY MATERIAL”

Exploring the role of Octanol-water partition coefficient and Henry’s law constant in predicting the lipid-water partition coefficients of organic chemicals

Muhammad Irfan Khawar^1,2^, Azhar Mahmood^3^, Deedar Nabi^1,2*^

^1^Institute of Environmental Science and Engineering (IESE), School of Civil and Environmental Engineering (SCEE), National University of Sciences and Technology (NUST), H-12, Islamabad, Pakistan.

^2^Environment and Agriculture Laboratory, School of Interdisciplinary Engineering and Sciences (SINES), National University of Sciences and Technology (NUST), H-12, Islamabad, Pakistan.

^3^School of Natural Sciences (SNS), National University of Sciences and Technology (NUST), H-12, Islamabad, Pakistan

*Corresponding author

Table of Contents

[**Table S1**. Chemicals with their experimental values of logK_lw_, and predicted values of logK_ow_ and logK_aw_ used to calibrate storage lipid-water tp-LFER model 3](#_Toc112248187)

[**Table S2**. Chemicals with their experimental values of logK_pw_ and predicted values of logK_ow_ and logK_aw_ used to calibrate phospholipid-water tp-LFER model 18](#_Toc112248188)

[**Table S3**. Showing chemicals having experimental values of logK_ow_ and logK_aw_ from the logK_lw_ dataset. 25](#_Toc112248189)

[**Table S4**. Showing chemicals having experimental values of logK_ow_ and logK_aw_ from the logK_pw_ dataset. 37](#_Toc112248190)

[**Table S5**. Showing chemicals with their experimental and estimated values (bold format) of logK_ow_ and logK_aw_ from EPI Suite used to formulate tp-LFER model equation of logK_lw_. 42](#_Toc112248191)

[**Table S6**. Showing chemicals with their experimental and estimated values (bold format) of logK_ow_ and logK_aw_ from EPI Suite used to formulate tp-LFER model equation of logK_pw_. 59](#_Toc112248192)

[**Table S7**. Showing chemicals with their estimated values of logK_ow_ and logK_aw_ from EPI Suite and estimated logK_lw_ from tp-LFER model equation in comparison to experimental logK_lw_. 67](#_Toc112248193)

[**Table S8**. Showing chemicals with their estimated values of logK_ow_ and logK_aw_ from EPI Suite and estimated logK_pw_ from tp-LFER model equation in comparison to experimental logK_pw_. 84](#_Toc112248194)

[**Table S9.** Showing the summary of results of existing models with the new developed models of current study for the estimation of logK_lw_ and logK_pw_. 92](#_Toc112248195)

[**Table S10**. Training set for logK_lw_ 92](#_Toc112248196)

[**Table S11**. Validation set for logK_lw_ 106](#_Toc112248197)

[**Table S12**. Training set for logK_pw_ 109](#_Toc112248198)

[**Table S13**. Validation set for logK_pw_ 116](#_Toc112248199)

[**Table S14**. Test set used to predict logK_lw_ with their experimental values of logK_tw_ and predicted values of logK_lw_ from tp-LFER model and the residuals 117](#_Toc112248200)

[**Table S15**. Test set used to predict logK_pw_ with their experimental values of logK_lipw_ and predicted values of logK_pw_ from tp-LFER model and the residuals 119](#_Toc112248201)

[**Figure S1**. The values of absolute residuals for op-LFER and tp-LFER as a function of Abraham solute parameter B, for organochlorine pesticides. 122](#_Toc112248202)

[**Section 1**. Cross Validation of tp-LFER Models 122](#_Toc112248203)

[**Table S16.** Cross-Validation Test for tp-LFER model of logK_lw_ (n=305) 123](#_Toc112248204)

[**Table S17.** Cross-Validation Test for tp-LFER model of logK_pw_ (n=131) 123](#_Toc112248205)

[**Section 2**. Dimensionality analysis of all models 124](#_Toc112248206)

[**Figure S2**. PCA plot on Abraham solute descriptors for (a) logK_lw_ and (b) logK_pw_ tp-LFER models 124](#_Toc112248207)

[**Figure S3**. Scree plot (a) and correlation circle (b) of logKlw tp-LFER model 124](#_Toc112248208)

[**Figure S4**. Scree plot (a) and correlation circle (b) of logK_pw_tp-LFER model 125](#_Toc112248209)

[**Section 3**. Application domain of all models (flagged chemicals) 125](#_Toc112248210)

[**Table S18.** List of flagged chemicals for logK_lw_ tp-LFER model 125](#_Toc112248211)

[**Table S19.** List of flagged chemicals for logK_pw_ tp-LFER model 126](#_Toc112248212)

[**Section 4.** One parameter LFER (op-LFER) equations for all models 126](#_Toc112248213)

[**Section 5.** Test sets 127](#_Toc112248214)

[References 127](#_Toc112248215)

## **Table S1**. Chemicals with their experimental values of logK_lw_, and predicted values of logK_ow_ and logK_aw_ used to calibrate storage lipid-water tp-LFER model

| **S. No** | **Chemicals** | **CAS No** | **logK_lw_** | **logK_ow_** | **logK_aw_** | **E** | **S** | **A** | **B** | **V** | **L** |
| --- | --- | --- | --- | --- | --- | --- | --- | --- | --- | --- | --- |
| 1 | 1,1,1,2-Tetrachloroethane | 630-20-6 | 2.85 | 2.84 | -0.97 | 0.54 | 0.63 | 0.1 | 0.08 | 0.88 | 3.641 |
| 2 | 1,1,1-Trichloroethane | 71-55-6 | 2.66 | 2.45 | -0.08 | 0.37 | 0.41 | 0 | 0.09 | 0.7576 | 2.733 |
| 3 | 1,1,2,2-Tetrachloroethane | 79-34-5 | 2.46 | 2.58 | -1.74 | 0.6 | 0.76 | 0.16 | 0.12 | 0.88 | 3.803 |
| 4 | 1,1,2-Trichloroethane | 79-00-5 | 2.09 | 2.32 | -1.33 | 0.5 | 0.68 | 0.13 | 0.13 | 0.7576 | 3.29 |
| 5 | 1,1-Dichloro-1-fluoroethane | 1717-00-6 | 1.34 | 1.96 | 0.16 | 0.08 | 0.43 | 0.01 | 0.05 | 0.6529 | 1.92 |
| 6 | 1,1-Dichloroethane | 75-34-3 | 1.74 | 1.81 | -0.77 | 0.32 | 0.49 | 0.1 | 0.1 | 0.6352 | 2.316 |
| 7 | 1,1-Dichloroethene | 75-35-4 | 2.19 | 2.02 | 0.14 | 0.36 | 0.34 | 0 | 0.05 | 0.5922 | 2.11 |
| 8 | 1,2,3-Trimethylbenzene | 526-73-8 | 3.32 | 3.53 | -0.88 | 0.73 | 0.61 | 0 | 0.19 | 1.1391 | 4.565 |
| 9 | 1,2,4-Trifluorobenzene | 367-23-7 | 2.67 | 2.31 | -0.38 | 0.31 | 0.65 | 0 | 0.05 | 0.7695 | 2.801 |
| 10 | 1,2,4-Trimethylbenzene | 95-63-6 | 3.43 | 3.55 | -0.72 | 0.68 | 0.56 | 0 | 0.19 | 1.1391 | 4.441 |
| 11 | 1,2-Dibromoethane | 106-93-4 | 1.67 | 1.90 | -1.91 | 0.75 | 0.76 | 0.1 | 0.17 | 0.7404 | 3.382 |
| 12 | 1,2-Dichlorobenzene | 95-50-1 | 3.71 | 3.36 | -0.94 | 0.87 | 0.78 | 0 | 0.04 | 0.9612 | 4.518 |
| 13 | 1,2-Dichloroethane | 107-06-2 | 1.60 | 1.68 | -1.25 | 0.42 | 0.64 | 0.1 | 0.11 | 0.6352 | 2.573 |
| 14 | 1,2-Dichloropropane | 78-87-5 | 1.99 | 1.96 | -0.92 | 0.37 | 0.63 | 0 | 0.17 | 0.7761 | 2.836 |
| 15 | 1,2-Difluorobenzene | 367-11-3 | 2.62 | 2.28 | -0.45 | 0.39 | 0.63 | 0 | 0.06 | 0.7518 | 2.843 |
| 16 | 1,3,5-Trifluorobenzene | 372-38-3 | 2.93 | 2.53 | 0.13 | 0.27 | 0.37 | 0 | 0.08 | 0.7695 | 2.641 |
| 17 | 1,3,5-Trimethylbenzene | 108-67-8 | 3.49 | 3.56 | -0.59 | 0.65 | 0.52 | 0 | 0.19 | 1.1391 | 4.344 |
| 18 | 1,3-Dichlorobenzene | 541-73-1 | 3.84 | 3.45 | -0.68 | 0.85 | 0.73 | 0 | 0.02 | 0.9612 | 4.41 |
| 19 | 1,4-Difluorobenzene | 540-36-3 | 2.58 | 2.29 | -0.35 | 0.38 | 0.6 | 0 | 0.06 | 0.7518 | 2.766 |
| 20 | 1-Bromo-2-chloroethane | 107-04-0 | 2.07 | 1.96 | -1.34 | 0.57 | 0.7 | 0.1 | 0.09 | 0.6878 | 2.982 |
| 21 | 1-Butanol | 71-36-3 | 0.02 | 0.91 | -3.36 | 0.22 | 0.42 | 0.37 | 0.48 | 0.7309 | 2.601 |
| 22 | 1-Chlorobutane | 109-69-3 | 2.78 | 2.52 | -0.01 | 0.21 | 0.4 | 0 | 0.1 | 0.7946 | 2.722 |
| 23 | 1-Chloropentane | 543-59-9 | 3.48 | 3.07 | 0.11 | 0.21 | 0.38 | 0 | 0.09 | 0.9355 | 3.223 |
| 24 | 1-Chloropropane | 540-54-5 | 2.22 | 1.95 | -0.12 | 0.22 | 0.4 | 0 | 0.1 | 0.6537 | 2.202 |
| 25 | 1-Hexanol | 111-27-3 | 1.36 | 2.02 | -3.13 | 0.21 | 0.42 | 0.37 | 0.48 | 1.0127 | 3.61 |
| 26 | 1-Methoxy-2-propanol | 107-98-2 | -1.53 | -0.26 | -4.92 | 0.22 | 0.53 | 0.33 | 0.81 | 0.7896 | 2.655 |
| 27 | 1-Nitropropane | 108-03-2 | 0.97 | 0.88 | -2.48 | 0.24 | 0.95 | 0 | 0.31 | 0.7055 | 2.894 |
| 28 | 1-Pentanol | 71-41-0 | 0.54 | 1.46 | -3.24 | 0.22 | 0.42 | 0.37 | 0.48 | 0.8718 | 3.106 |
| 29 | 1-Propanol | 71-23-8 | -0.48 | 0.32 | -3.45 | 0.24 | 0.42 | 0.37 | 0.48 | 0.59 | 2.031 |
| 30 | 2,2,4-Trimethylpentane | 540-84-1 | 4.64 | 4.65 | 2.25 | 0 | 0 | 0 | 0 | 1.2358 | 3.106 |
| 31 | 2,2-Dichloro-1,1,1-trifluoroethane | 306-83-2 | 1.81 | 2.15 | -0.13 | -0.09 | 0.34 | 0.21 | 0 | 0.6883 | 1.746 |
| 32 | 2,2-Dimethylbutane | 75-83-2 | 3.79 | 3.65 | 1.89 | 0 | 0 | 0 | 0 | 0.954 | 2.352 |
| 33 | 2,3,4-Trimethylpentane | 565-75-3 | 5.05 | 4.82 | 2.07 | 0 | 0 | 0 | 0 | 1.2358 | 3.481 |
| 34 | 2-Butoxyethanol | 111-76-2 | -0.21 | 0.94 | -4.68 | 0.2 | 0.53 | 0.26 | 0.83 | 1.0714 | 3.656 |
| 35 | 2-Chloropropane | 75-29-6 | 2.03 | 1.86 | 0.00 | 0.18 | 0.35 | 0 | 0.12 | 0.6537 | 1.97 |
| 36 | 2-Ethoxyethanol | 110-80-5 | -1.27 | -0.17 | -4.93 | 0.24 | 0.55 | 0.29 | 0.82 | 0.7896 | 2.719 |
| 37 | 2-Fluoropropane | 420-26-8 | 1.38 | 1.33 | 0.33 | 0.03 | 0.21 | 0 | 0.12 | 0.549 | 1.15 |
| 38 | 2-Heptanone | 110-43-0 | 1.81 | 1.92 | -2.27 | 0.12 | 0.68 | 0 | 0.51 | 1.1106 | 3.76 |
| 39 | 2-Hexanone | 591-78-6 | 1.19 | 1.37 | -2.41 | 0.14 | 0.68 | 0 | 0.51 | 0.9697 | 3.286 |
| 40 | 2-Isopropoxyethanol | 109-59-1 | -1.16 | 0.11 | -4.78 | 0.2 | 0.48 | 0.21 | 0.91 | 0.9305 | 3.214 |
| 41 | 2-Methoxyethanol | 109-86-4 | -1.67 | -0.68 | -5.18 | 0.27 | 0.5 | 0.3 | 0.84 | 0.6487 | 2.49 |
| 42 | 2-Methyl-1-propanol | 78-83-1 | -0.06 | 0.87 | -3.21 | 0.22 | 0.39 | 0.37 | 0.48 | 0.7309 | 2.413 |
| 43 | 2-Methyl-2-propanol | 75-65-0 | -0.73 | 0.40 | -3.17 | 0.18 | 0.3 | 0.31 | 0.6 | 0.7309 | 1.963 |
| 44 | 2-Methylpentane | 107-83-5 | 3.99 | 3.72 | 1.82 | 0 | 0 | 0 | 0 | 0.954 | 2.503 |
| 45 | 2-Nitropropane | 79-46-9 | 0.61 | 0.74 | -2.30 | 0.22 | 0.92 | 0 | 0.33 | 0.7055 | 2.55 |
| 46 | 2-Pentanone | 107-87-9 | 0.65 | 0.80 | -2.51 | 0.14 | 0.68 | 0 | 0.51 | 0.8288 | 2.755 |
| 47 | 2-Propanol | 67-63-0 | -0.82 | 0.02 | -3.44 | 0.21 | 0.36 | 0.33 | 0.56 | 0.59 | 1.764 |
| 48 | 3-Methyl-1-butanol | 137-32-6 | 0.41 | 1.46 | -3.13 | 0.22 | 0.39 | 0.37 | 0.48 | 0.8718 | 3.011 |
| 49 | 3-Methylhexane | 589-34-4 | 4.61 | 4.29 | 1.92 | 0 | 0 | 0 | 0 | 1.0949 | 3.044 |
| 50 | 3-Methylpentane | 96-14-0 | 4.07 | 3.75 | 1.78 | 0 | 0 | 0 | 0 | 0.954 | 2.581 |
| 51 | 3-Pentanone | 96-22-0 | 0.56 | 0.86 | -2.50 | 0.15 | 0.66 | 0 | 0.51 | 0.8288 | 2.811 |
| 52 | 4-Methyl-2-pentanone | 108-10-1 | 1.05 | 1.33 | -2.25 | 0.11 | 0.65 | 0 | 0.51 | 0.9697 | 3.089 |
| 53 | Acetone | 67-64-1 | -0.32 | -0.30 | -2.81 | 0.18 | 0.7 | 0.04 | 0.49 | 0.547 | 1.696 |
| 54 | Allylbenzene | 300-57-2 | 2.96 | 3.16 | -0.91 | 0.72 | 0.6 | 0 | 0.22 | 1.0961 | 4.136 |
| 55 | Benzene | 71-43-2 | 2.12 | 2.05 | -0.68 | 0.61 | 0.52 | 0 | 0.14 | 0.7164 | 2.786 |
| 56 | Bromochloromethane | 74-97-5 | 1.49 | 1.37 | -1.17 | 0.54 | 0.8 | 0.01 | 0.06 | 0.5469 | 2.445 |
| 57 | 2-Butanone | 78-93-3 | 0.18 | 0.23 | -2.69 | 0.17 | 0.7 | 0 | 0.51 | 0.6879 | 2.287 |
| 58 | Butane | 106-97-8 | 3.03 | 2.65 | 1.53 | 0 | 0 | 0 | 0 | 0.6722 | 1.615 |
| 59 | Butyl acetate | 123-86-4 | 1.59 | 1.86 | -1.83 | 0.07 | 0.6 | 0 | 0.45 | 1.0284 | 3.353 |
| 60 | Carbon tetrachloride | 56-23-5 | 3.18 | 2.80 | 0.33 | 0.46 | 0.38 | 0 | 0 | 0.7391 | 2.823 |
| 61 | 1,2-Dichlorotetrafluoroethane | 76-14-2 | 3.00 | 2.58 | 1.60 | -0.11 | 0.02 | 0 | 0 | 0.706 | 1.414 |
| 62 | 1,1,2,2,3,3,4,4-Octafluorobutane | 377-36-6 | 2.50 | 2.27 | 0.52 | -0.79 | 0.08 | 0.15 | 0.05 | 0.8138 | 1.456 |
| 63 | 1,1,2,2,3,3-Hexafluoropropane | 680-00-2 | 1.49 | 1.48 | 0.45 | -0.59 | 0.21 | 0.15 | 0.1 | 0.6375 | 0.621 |
| 64 | 1,1,2,2-Tetrafluoroethane | 359-35-3 | 0.92 | 0.85 | 0.13 | -0.39 | 0.24 | 0.1 | 0.12 | 0.4612 | 0.394 |
| 65 | 1,1-Difluoroethane | 75-37-6 | 0.91 | 0.70 | -0.06 | -0.22 | 0.46 | 0.03 | 0.07 | 0.4258 | 0.561 |
| 66 | Halothane | 151-67-7 | 2.28 | 2.40 | -0.05 | 0.1 | 0.39 | 0.13 | 0.05 | 0.7409 | 1.982 |
| 67 | 1,1,1,2-Tetrafluoroethane | 811-97-2 | 1.03 | 1.20 | 0.41 | -0.41 | 0.41 | 0.06 | 0.02 | 0.4612 | 0.53 |
| 68 | Fluroxene | 406-90-6 | 1.26 | 1.46 | -0.22 | 0.18 | 0.3 | 0 | 0.27 | 0.741 | 1.6 |
| 69 | Carbon tetrafluoride | 75-73-0 | 1.12 | 1.13 | 2.34 | -0.55 | -0.25 | 0 | 0 | 0.3203 | -0.819 |
| 70 | 1,3-Difluoropropane | 462-39-5 | 0.66 | 0.76 | -1.21 | -0.2 | 0.55 | 0.12 | 0.21 | 0.5667 | 1.35 |
| 71 | Enflurane | 13838-16-9 | 2.03 | 2.03 | 0.03 | -0.24 | 0.4 | 0.07 | 0.13 | 0.8009 | 1.75 |
| 72 | Isoflurane | 26675-46-7 | 1.99 | 1.88 | -0.01 | -0.24 | 0.56 | 0 | 0.08 | 0.8009 | 1.969 |
| 73 | Desflurane | 57041-67-5 | 1.88 | 1.76 | 0.73 | -0.47 | 0.38 | 0.05 | 0.04 | 0.6962 | 0.99 |
| 74 | Sevoflurane | 28523-86-6 | 1.87 | 1.91 | 0.40 | -0.47 | 0.56 | 0 | 0.1 | 0.8548 | 1.502 |
| 75 | Chlorobenzene | 108-90-7 | 2.73 | 2.78 | -0.71 | 0.72 | 0.65 | 0 | 0.07 | 0.8388 | 3.657 |
| 76 | Chlorodibromomethane | 124-48-1 | 2.12 | 2.18 | -1.49 | 0.78 | 0.68 | 0.12 | 0.1 | 0.7219 | 3.304 |
| 77 | Chloroethane | 75-00-3 | 1.58 | 1.39 | -0.22 | 0.23 | 0.4 | 0 | 0.1 | 0.5128 | 1.678 |
| 78 | Chloroform | 67-66-3 | 2.02 | 2.11 | -0.69 | 0.43 | 0.49 | 0.15 | 0.02 | 0.6167 | 2.48 |
| 79 | cis-1,2-Dichloroethene | 156-59-2 | 1.59 | 1.76 | -0.98 | 0.44 | 0.61 | 0.11 | 0.05 | 0.5922 | 2.439 |
| 80 | Cycloheptane | 291-64-5 | 4.82 | 4.17 | 1.12 | 0.35 | 0.1 | 0 | 0 | 0.9863 | 3.704 |
| 81 | Cyclohexane | 110-82-7 | 4.05 | 3.51 | 1.12 | 0.31 | 0.1 | 0 | 0 | 0.8454 | 2.964 |
| 82 | Cyclopentane | 287-92-3 | 3.50 | 2.96 | 0.99 | 0.26 | 0.1 | 0 | 0 | 0.7045 | 2.477 |
| 83 | Cyclopropane | 75-19-4 | 1.73 | 1.60 | 0.56 | 0.41 | 0.23 | 0 | 0 | 0.4227 | 1.314 |
| 84 | Decane | 124-18-5 | 6.39 | 6.01 | 2.21 | 0 | 0 | 0 | 0 | 1.5176 | 4.686 |
| 85 | Dibromethane | 74-95-3 | 1.76 | 1.72 | -1.51 | 0.71 | 0.69 | 0.11 | 0.07 | 0.5995 | 2.886 |
| 86 | Dichloromethane | 75-09-2 | 1.28 | 1.41 | -0.91 | 0.39 | 0.57 | 0.1 | 0.05 | 0.4943 | 2.019 |
| 87 | Diethyl ether | 60-29-7 | 0.79 | 1.08 | -1.19 | 0.04 | 0.25 | 0 | 0.45 | 0.7309 | 2.015 |
| 88 | Difluoromethane | 75-10-5 | 0.53 | 0.17 | -0.18 | -0.32 | 0.58 | 0.03 | 0.03 | 0.2849 | 0.025 |
| 89 | Divinyl ether | 109-93-3 | 1.69 | 1.65 | -0.05 | 0.26 | 0.39 | 0 | 0.13 | 0.6449 | 1.76 |
| 90 | Ethane | 74-84-0 | 1.73 | 1.49 | 1.35 | 0 | 0 | 0 | 0 | 0.3904 | 0.492 |
| 91 | Ethanol | 64-17-5 | -1.14 | -0.25 | -3.54 | 0.25 | 0.42 | 0.37 | 0.48 | 0.4491 | 1.485 |
| 92 | Ethene | 74-85-1 | 0.84 | 0.92 | 0.79 | 0.11 | 0.1 | 0 | 0.07 | 0.3474 | 0.289 |
| 93 | Ethyl acetate | 141-78-6 | 0.46 | 0.71 | -2.09 | 0.11 | 0.62 | 0 | 0.45 | 0.7466 | 2.314 |
| 94 | Ethyl tert-butyl ether | 637-92-3 | 1.37 | 1.72 | -1.16 | 0 | 0.16 | 0 | 0.57 | 1.0127 | 2.652 |
| 95 | Ethyl tert-pentyl ether | 919-94-8 | 1.93 | 2.19 | -1.33 | -0.1 | 0.19 | 0 | 0.6 | 1.1536 | 3.074 |
| 96 | Ethylbenzene | 100-41-4 | 3.14 | 3.13 | -0.46 | 0.61 | 0.51 | 0 | 0.15 | 0.9982 | 3.778 |
| 97 | Fluorobenzene | 462-06-6 | 2.41 | 2.16 | -0.54 | 0.48 | 0.57 | 0 | 0.1 | 0.7341 | 2.788 |
| 98 | Fluoroethane | 353-36-6 | 0.62 | 0.73 | 0.14 | 0.05 | 0.34 | 0 | 0.05 | 0.4081 | 0.751 |
| 99 | Fluorochloromethane | 593-70-4 | 0.77 | 0.69 | -0.60 | 0.04 | 0.61 | 0.07 | 0.04 | 0.3896 | 0.982 |
| 100 | Heptane | 142-82-5 | 4.71 | 4.34 | 1.86 | 0 | 0 | 0 | 0 | 1.0949 | 3.173 |
| 101 | Hexafluorobenzene | 392-56-3 | 2.45 | 2.51 | 0.35 | 0.09 | 0.56 | 0 | 0.01 | 0.8226 | 2.345 |
| 102 | Hexane | 110-54-3 | 4.11 | 3.79 | 1.74 | 0 | 0 | 0 | 0 | 0.954 | 2.668 |
| 103 | Isobutyl acetate | 110-19-0 | 1.60 | 1.75 | -1.77 | 0.05 | 0.57 | 0 | 0.47 | 1.0284 | 3.161 |
| 104 | Isopentyl acetate | 123-92-2 | 2.11 | 2.34 | -1.69 | 0.05 | 0.57 | 0 | 0.47 | 1.1693 | 3.74 |
| 105 | Isopropyl acetate | 108-21-4 | 1.02 | 1.15 | -1.84 | 0.06 | 0.57 | 0 | 0.47 | 0.8875 | 2.546 |
| 106 | Isopropylbenzene | 98-82-8 | 3.52 | 3.60 | -0.26 | 0.6 | 0.49 | 0 | 0.16 | 1.1391 | 4.084 |
| 107 | Methoxyflurane | 76-38-0 | 2.11 | 2.52 | -0.82 | 0.2 | 0.42 | 0.18 | 0.14 | 0.8702 | 2.831 |
| 108 | Methane | 74-82-8 | 0.75 | 0.80 | 1.38 | 0 | 0 | 0 | 0 | 0.2495 | -0.323 |
| 109 | Methanol | 67-56-1 | -1.95 | -0.82 | -3.87 | 0.28 | 0.44 | 0.43 | 0.47 | 0.3082 | 0.97 |
| 110 | Methyl acetate | 79-20-9 | -0.02 | 0.17 | -2.30 | 0.14 | 0.64 | 0 | 0.45 | 0.6057 | 1.911 |
| 111 | Methyl chloride | 74-87-3 | 0.80 | 0.85 | -0.30 | 0.25 | 0.43 | 0 | 0.08 | 0.3719 | 1.163 |
| 112 | Methylcyclopentane | 96-37-7 | 4.00 | 3.45 | 1.19 | 0.23 | 0.1 | 0 | 0 | 0.8454 | 2.907 |
| 113 | Methylpentafluorobenzene | 771-56-2 | 3.27 | 3.15 | 0.17 | 0.16 | 0.59 | 0 | 0.01 | 0.9458 | 3.244 |
| 114 | m-Methylstyrene | 100-80-1 | 3.23 | 3.33 | -0.94 | 0.87 | 0.65 | 0 | 0.18 | 1.0961 | 4.375 |
| 115 | m-Xylene | 108-38-3 | 3.16 | 3.11 | -0.56 | 0.62 | 0.52 | 0 | 0.16 | 0.9982 | 3.839 |
| 116 | Nonane | 111-84-2 | 5.82 | 5.46 | 2.09 | 0 | 0 | 0 | 0 | 1.3767 | 4.182 |
| 117 | o-Xylene | 95-47-6 | 3.12 | 3.10 | -0.69 | 0.66 | 0.56 | 0 | 0.16 | 0.9982 | 3.939 |
| 118 | p-Xylene | 106-42-3 | 3.16 | 3.11 | -0.56 | 0.61 | 0.52 | 0 | 0.16 | 0.9982 | 3.839 |
| 119 | Pentachloroethane | 76-01-7 | 2.93 | 3.42 | -1.18 | 0.65 | 0.66 | 0.17 | 0.06 | 1.0024 | 4.267 |
| 120 | Pentafluorobenzene | 363-72-4 | 2.31 | 2.36 | -0.10 | 0.15 | 0.58 | 0.06 | 0.02 | 0.8049 | 2.614 |
| 121 | Pentane | 109-66-0 | 3.49 | 3.23 | 1.63 | 0 | 0 | 0 | 0 | 0.8131 | 2.162 |
| 122 | Pentyl acetate | 628-63-7 | 2.11 | 2.41 | -1.71 | 0.07 | 0.6 | 0 | 0.45 | 1.1693 | 3.844 |
| 123 | p-Methylstyrene | 622-97-9 | 3.21 | 3.34 | -0.95 | 0.87 | 0.65 | 0 | 0.18 | 1.0961 | 4.399 |
| 124 | Propane | 74-98-6 | 2.35 | 2.07 | 1.44 | 0 | 0 | 0 | 0 | 0.5313 | 1.05 |
| 125 | Propyl acetate | 109-60-4 | 1.02 | 1.29 | -1.93 | 0.09 | 0.6 | 0 | 0.45 | 0.8875 | 2.819 |
| 126 | Propylbenzene | 103-65-1 | 3.65 | 3.68 | -0.30 | 0.6 | 0.5 | 0 | 0.15 | 1.1391 | 4.23 |
| 127 | Tetrachloroethene | 127-18-4 | 3.57 | 3.28 | 0.09 | 0.64 | 0.44 | 0 | 0 | 0.837 | 3.584 |
| 128 | Toluene | 108-88-3 | 2.67 | 2.62 | -0.58 | 0.6 | 0.52 | 0 | 0.14 | 0.8573 | 3.325 |
| 129 | trans-1,2-Dichloroethene | 156-60-5 | 2.06 | 1.98 | -0.42 | 0.43 | 0.41 | 0.09 | 0.05 | 0.5922 | 2.278 |
| 130 | Trichloroethene | 79-01-6 | 2.80 | 2.71 | -0.23 | 0.52 | 0.37 | 0.08 | 0.03 | 0.7146 | 2.997 |
| 131 | tridecane | 629-50-5 | 8.16 | 7.68 | 2.56 | 0 | 0 | 0 | 0 | 1.9403 | 6.2 |
| 132 | triethylamine | 121-44-8 | 1.04 | 1.25 | -2.34 | 0.1 | 0.15 | 0 | 0.79 | 1.0538 | 3.04 |
| 133 | undecane | 1120-21-4 | 7.03 | 6.57 | 2.33 | 0 | 0 | 0 | 0 | 1.6585 | 5.191 |
| 134 | alpha-pinene | 80-56-8 | 4.58 | 4.61 | 0.85 | 0.44 | 0.2 | 0 | 0.14 | 1.2574 | 4.256 |
| 135 | 1,2-dimethoxyethane | 110-71-4 | -0.33 | 0.09 | -3.37 | 0.12 | 0.67 | 0 | 0.68 | 0.7896 | 2.654 |
| 136 | 1,4-dioxane | 123-91-1 | -0.24 | -0.04 | -3.73 | 0.33 | 0.75 | 0 | 0.64 | 0.681 | 2.892 |
| 137 | 1-heptanol | 111-70-6 | 1.84 | 2.57 | -3.01 | 0.21 | 0.42 | 0.37 | 0.48 | 1.1536 | 4.115 |
| 138 | 2-methylpyridine | 109-06-8 | 0.61 | 0.75 | -3.30 | 0.6 | 0.75 | 0 | 0.58 | 0.8162 | 3.422 |
| 139 | 3-methylpyridine | 108-99-6 | 0.84 | 0.86 | -3.38 | 0.63 | 0.81 | 0 | 0.54 | 0.8162 | 3.631 |
| 140 | 4-methylpyridine | 108-89-4 | 0.82 | 0.82 | -3.45 | 0.63 | 0.82 | 0 | 0.54 | 0.8162 | 3.64 |
| 141 | benzyl alcohol | 100-51-6 | 0.12 | 1.14 | -4.84 | 0.8 | 0.87 | 0.39 | 0.56 | 0.916 | 4.221 |
| 142 | bromobenzene | 108-86-1 | 3.27 | 2.89 | -1.03 | 0.88 | 0.73 | 0 | 0.09 | 0.8914 | 4.041 |
| 143 | butyl formate | 592-84-7 | 1.46 | 1.55 | -1.72 | 0.12 | 0.63 | 0 | 0.38 | 0.8875 | 2.958 |
| 144 | butyl propanoate | 590-01-2 | 2.35 | 2.40 | -1.72 | 0.06 | 0.56 | 0 | 0.47 | 1.1693 | 3.833 |
| 145 | butylbenzene | 104-51-8 | 4.13 | 4.22 | -0.20 | 0.6 | 0.51 | 0 | 0.15 | 1.28 | 4.73 |
| 146 | 1,1-difluoro-2-chloroethene | 359-10-4 | 1.63 | 1.45 | 0.40 | -0.34 | 0.28 | 0.15 | 0 | 0.5052 | 0.723 |
| 147 | 1-chloro-2,2,2-trifluoroethane | 75-88-7 | 1.34 | 1.62 | 0.09 | 0.01 | 0.4 | 0.15 | 0 | 0.5659 | 1.168 |
| 148 | bis-(2,2,2-trifluoroethyl)ether | 333-36-8 | 1.92 | 1.69 | -0.09 | -0.47 | 0.12 | 0.07 | 0.32 | 0.8371 | 1.419 |
| 149 | cyclohexene | 110-83-8 | 3.33 | 2.95 | 0.29 | 0.4 | 0.28 | 0 | 0.09 | 0.8024 | 2.952 |
| 150 | cyclopentanone | 120-92-3 | 0.39 | 0.45 | -3.43 | 0.37 | 0.86 | 0 | 0.52 | 0.7202 | 3.221 |
| 151 | difluorochloromethane | 75-45-6 | 0.72 | 0.90 | 0.12 | -0.06 | 0.38 | 0.04 | 0.05 | 0.4073 | 0.692 |
| 152 | diisopropyl ether | 108-20-3 | 1.29 | 1.72 | -1.08 | -0.06 | 0.17 | 0 | 0.57 | 1.0127 | 2.501 |
| 153 | dimethoxymethane | 109-87-5 | 0.18 | 0.28 | -2.15 | 0.1 | 0.46 | 0 | 0.52 | 0.6487 | 1.894 |
| 154 | dimethyl ether | 115-10-6 | 0.13 | 0.18 | -1.44 | 0 | 0.27 | 0 | 0.41 | 0.4491 | 1.285 |
| 155 | dimethylacetamide | 127-19-5 | -1.25 | -0.76 | -5.69 | 0.36 | 1.38 | 0 | 0.8 | 0.7877 | 3.639 |
| 156 | dimethylformamide | 68-12-2 | -1.57 | -1.10 | -5.55 | 0.37 | 1.31 | 0 | 0.74 | 0.6468 | 3.173 |
| 157 | di-n-butyl ether | 142-96-1 | 3.14 | 3.24 | -0.70 | 0 | 0.25 | 0 | 0.45 | 1.2945 | 3.924 |
| 158 | dodecane | 112-40-3 | 7.59 | 7.13 | 2.44 | 0 | 0 | 0 | 0 | 1.7994 | 5.696 |
| 159 | ethyl formate | 109-94-4 | 0.16 | 0.35 | -1.97 | 0.15 | 0.66 | 0 | 0.38 | 0.6057 | 1.845 |
| 160 | ethyl propanoate | 105-37-3 | 1.13 | 1.32 | -1.89 | 0.09 | 0.58 | 0 | 0.45 | 0.8875 | 2.807 |
| 161 | iodoethane | 75-03-6 | 1.85 | 1.93 | -0.55 | 0.64 | 0.4 | 0 | 0.14 | 0.6486 | 2.573 |
| 162 | fluorotrichloromethane | 75-69-4 | 2.28 | 2.13 | 0.43 | 0.21 | 0.24 | 0 | 0.07 | 0.6344 | 1.95 |
| 163 | methyl formate | 107-31-3 | -0.48 | -0.26 | -2.10 | 0.19 | 0.68 | 0 | 0.38 | 0.4648 | 1.285 |
| 164 | methylcyclohexane | 108-87-2 | 4.54 | 4.00 | 1.30 | 0.24 | 0.06 | 0 | 0 | 0.9863 | 3.319 |
| 165 | nitroethane | 79-24-3 | 0.45 | 0.26 | -2.78 | 0.27 | 0.95 | 0.02 | 0.33 | 0.5646 | 2.414 |
| 166 | nitromethane | 75-52-5 | -0.02 | -0.28 | -2.98 | 0.31 | 0.95 | 0.06 | 0.31 | 0.4237 | 1.892 |
| 167 | N,N-dimethylaniline | 121-69-7 | 2.29 | 2.37 | -2.65 | 0.96 | 0.81 | 0 | 0.41 | 1.098 | 4.701 |
| 168 | pentadecane | 629-62-9 | 9.31 | 8.80 | 2.80 | 0 | 0 | 0 | 0 | 2.2221 | 7.209 |
| 169 | piperidine | 110-89-4 | 1.01 | 0.67 | -3.87 | 0.42 | 0.4 | 0.06 | 0.77 | 0.8043 | 3.075 |
| 170 | propyl bromide | 106-94-5 | 2.47 | 2.19 | -0.28 | 0.37 | 0.4 | 0 | 0.12 | 0.7063 | 2.62 |
| 171 | propyl formate | 110-74-7 | 0.87 | 0.99 | -1.83 | 0.13 | 0.63 | 0 | 0.38 | 0.7466 | 2.433 |
| 172 | pyridine | 110-86-1 | 0.15 | 0.29 | -3.41 | 0.63 | 0.84 | 0 | 0.52 | 0.6753 | 3.022 |
| 173 | tetradecane | 629-59-4 | 8.74 | 8.24 | 2.68 | 0 | 0 | 0 | 0 | 2.0812 | 6.705 |
| 174 | tetrahydrofuran | 109-99-9 | 0.62 | 0.58 | -2.50 | 0.29 | 0.52 | 0 | 0.48 | 0.6223 | 2.636 |
| 175 | halopropane | 679-84-5 | 2.91 | 2.65 | 0.28 | -0.06 | 0.53 | 0.05 | 0.2 | 0.7771 | 2.388 |
| 176 | Cyclooctane | 292-64-8 | 5.34 | 4.78 | 1.18 | 0.41 | 0.1 | 0 | 0 | 1.1272 | 4.329 |
| 177 | Octan-1-ol | 111-87-5 | 2.46 | 3.13 | -2.89 | 0.2 | 0.42 | 0.37 | 0.48 | 1.2945 | 4.619 |
| 178 | Nonan-1-ol | 143-08-8 | 3.01 | 3.69 | -2.77 | 0.19 | 0.42 | 0.37 | 0.48 | 1.4354 | 5.12 |
| 179 | Hexanal | 66-25-1 | 1.92 | 1.65 | -2.09 | 0.15 | 0.65 | 0 | 0.45 | 0.9697 | 3.357 |
| 180 | Heptanal | 111-71-7 | 2.46 | 2.21 | -1.97 | 0.14 | 0.65 | 0 | 0.45 | 1.1106 | 3.865 |
| 181 | Octanal | 124-13-0 | 3.01 | 2.76 | -1.85 | 0.14 | 0.65 | 0 | 0.45 | 1.2515 | 4.361 |
| 182 | Nonanal / n-Nonyl Aldehyde | 124-19-6 | 3.42 | 3.31 | -1.73 | 0.15 | 0.65 | 0 | 0.45 | 1.3924 | 4.834 |
| 183 | 1-Chloroheptane | 629-06-1 | 4.37 | 4.21 | 0.32 | 0.19 | 0.4 | 0 | 0.09 | 1.2173 | 4.208 |
| 184 | 1-Chlorooctane | 111-85-3 | 4.95 | 4.76 | 0.53 | 0.19 | 0.4 | 0 | 0.09 | 1.3582 | 4.708 |
| 185 | 1-Hexene | 592-41-6 | 3.51 | 3.29 | 1.17 | 0.08 | 0.08 | 0 | 0.07 | 0.911 | 2.572 |
| 186 | 1-Heptene | 592-76-7 | 4.04 | 3.84 | 1.30 | 0.09 | 0.08 | 0 | 0.07 | 1.0519 | 3.063 |
| 187 | 1-Octene | 111-66-0 | 4.60 | 4.39 | 1.41 | 0.09 | 0.08 | 0 | 0.07 | 1.1928 | 3.568 |
| 188 | 1-Nonene | 124-11-8 | 5.15 | 4.95 | 1.53 | 0.09 | 0.08 | 0 | 0.07 | 1.3337 | 4.073 |
| 189 | 1-Decene | 872-05-9 | 5.64 | 5.49 | 1.67 | 0.09 | 0.08 | 0 | 0.07 | 1.4746 | 4.533 |
| 190 | 1,2,4-Trichlorobenzene | 120-82-1 | 4.17 | 4.07 | -0.84 | 0.98 | 0.81 | 0 | 0 | 1.0836 | 5.248 |
| 191 | Di-n-propyl ether | 111-43-3 | 2.06 | 2.16 | -0.77 | 0.01 | 0.25 | 0 | 0.45 | 1.0127 | 2.954 |
| 192 | Dipentyl ether | 693-65-2 | 4.16 | 4.33 | -0.44 | 0 | 0.25 | 0 | 0.45 | 1.5763 | 4.875 |
| 193 | 2-octanone | 111-13-7 | 2.31 | 2.47 | -2.15 | 0.11 | 0.68 | 0 | 0.51 | 1.2515 | 4.257 |
| 194 | 2-nonanone | 821-55-6 | 2.79 | 3.01 | -2.02 | 0.11 | 0.68 | 0 | 0.51 | 1.3924 | 4.735 |
| 195 | 1-Nitrobutane | 627-05-4 | 1.53 | 1.51 | -2.27 | 0.23 | 0.95 | 0 | 0.29 | 0.8464 | 3.415 |
| 196 | 1-Nitrohexane | 646-14-0 | 2.58 | 2.62 | -2.03 | 0.2 | 0.95 | 0 | 0.29 | 1.1282 | 4.416 |
| 197 | 4-Ethylpyridine | 536-75-4 | 1.14 | 1.33 | -3.38 | 0.63 | 0.8 | 0 | 0.57 | 0.9571 | 4.124 |
| 198 | 1-Chloro-4-nitrobenzene | 100-00-5 | 2.38 | 2.53 | -2.94 | 0.98 | 1.22 | 0 | 0.24 | 1.013 | 5.22 |
| 199 | Nitrobenzene | 98-95-3 | 1.92 | 1.91 | -2.99 | 0.87 | 1.11 | 0 | 0.28 | 0.8906 | 4.557 |
| 200 | 2-Nitrotoluene | 88-72-2 | 2.40 | 2.39 | -2.78 | 0.87 | 1.11 | 0 | 0.28 | 1.0315 | 4.878 |
| 201 | 2,6-Dinitrotoluene | 606-20-2 | 1.94 | 2.16 | -4.69 | 1.15 | 1.55 | 0 | 0.45 | 1.2057 | 6.162 |
| 202 | 4-Nitroanisole | 100-17-4 | 2.32 | 1.77 | -4.67 | 0.98 | 1.49 | 0 | 0.37 | 1.0902 | 5.62 |
| 203 | 1,4-Dimethoxybenzene | 150-78-7 | 2.12 | 2.06 | -3.49 | 0.81 | 1 | 0 | 0.5 | 1.1156 | 5.044 |
| 204 | 4-Chlorophenol | 106-48-9 | 1.51 | 2.19 | -5.13 | 0.92 | 1.08 | 0.67 | 0.2 | 0.8975 | 4.775 |
| 205 | Ethyl benzoate | 93-89-0 | 2.59 | 2.66 | -2.75 | 0.69 | 0.85 | 0 | 0.46 | 1.2135 | 5.075 |
| 206 | Indole | 120-72-9 | 1.99 | 2.49 | -4.55 | 1.2 | 1.26 | 0.44 | 0.18 | 0.9464 | 5.31 |
| 207 | 2-Ethyl-1-hexanol | 104-76-7 | 2.04 | 3.09 | -2.74 | 0.21 | 0.39 | 0.37 | 0.48 | 1.2945 | 4.433 |
| 208 | 3-Ethyl-3-hexanol | 597-76-2 | 1.70 | 2.76 | -2.85 | 0.2 | 0.3 | 0.31 | 0.6 | 1.2945 | 4.29 |
| 209 | 4-Ethyl-3-hexanol | 19780-44-0 | 1.92 | 2.72 | -2.85 | 0.17 | 0.36 | 0.33 | 0.57 | 1.2945 | 4.177 |
| 210 | 3-Ethyl-3-pentanol | 597-49-9 | 1.13 | 2.08 | -3.19 | 0.23 | 0.3 | 0.31 | 0.64 | 1.1536 | 3.838 |
| 211 | 2,4-Dinitrotoluene | 121-14-2 | 2.34 | 1.42 | -5.88 | 1.15 | 1.58 | 0 | 0.49 | 1.2057 | 6.258 |
| 212 | 1-fluropropane | 460-13-9 | 0.98 | 1.20 | 0.10 | 0.03 | 0.21 | 0 | 0.12 | 0.549 | 1.251 |
| 213 | Hexachloroethane | 67-72-1 | 3.71 | 4.12 | -0.21 | 0.68 | 0.68 | 0 | 0 | 1.1248 | 4.573 |
| 214 | Biphenyl | 92-52-4 | 4.14 | 3.82 | -2.24 | 1.36 | 0.99 | 0 | 0.26 | 1.3242 | 6.014 |
| 215 | hexadecane | 544-76-3 | 9.88 | 9.35 | 2.91 | 0 | 0 | 0 | 0 | 2.363 | 7.714 |
| 216 | isopropyl bromide | 75-26-3 | 2.28 | 2.09 | -0.16 | 0.33 | 0.35 | 0 | 0.14 | 0.7063 | 2.39 |
| 217 | beta-pinene | 127-91-3 | 4.11 | 4.27 | 0.27 | 0.52 | 0.19 | 0 | 0.15 | 1.2574 | 4.515 |
| 218 | limonene | 138-86-3 | 4.17 | 4.31 | -0.05 | 0.5 | 0.31 | 0 | 0.23 | 1.323 | 4.688 |
| 219 | Fluoromethane | 593-53-3 | 0.00 | 0.20 | 0.08 | 0.07 | 0.35 | 0 | 0.09 | 0.2672 | 0.057 |
| 220 | Tricyclo[5.2.1.0(2,6)]decane | 2825-83-4 | 4.62 | 4.45 | 0.08 | 0.59 | 0.68 | 0 | 0.06 | 1.1918 | 4.651 |
| 221 | Methyl tert-butyl ether | 1634-04-4 | 0.89 | 1.16 | -1.49 | 0.02 | 0.22 | 0 | 0.55 | 0.8718 | 2.372 |
| 222 | vinyl chloride | 75-01-4 | 1.58 | 1.37 | 0.08 | 0.26 | 0.38 | 0 | 0.05 | 0.4698 | 1.404 |
| 223 | dimethyl sulfoxide | 67-68-5 | -2.66 | -2.19 | -7.40 | 0.52 | 1.72 | 0 | 0.97 | 0.6126 | 3.459 |
| 224 | formic acid | 64-18-6 | -1.69 | -0.55 | -5.27 | 0.34 | 0.75 | 0.76 | 0.33 | 0.3239 | 1.545 |
| 225 | 3-carene | 13466-78-9 | 4.71 | 4.71 | 0.62 | 0.49 | 0.22 | 0 | 0.14 | 1.2574 | 4.679 |
| 226 | vinyl bromide | 593-60-2 | 1.34 | 1.45 | -0.34 | 0.43 | 0.44 | 0 | 0.08 | 0.5224 | 1.887 |
| 227 | 4-Chloroaniline | 106-47-8 | 1.62 | 1.99 | -4.31 | 1.06 | 1.13 | 0.3 | 0.31 | 0.9386 | 4.889 |
| 228 | allyl chloride | 107-05-1 | 1.84 | 1.76 | -0.27 | 0.33 | 0.56 | 0 | 0.05 | 0.6107 | 2.109 |
| 229 | Styrene | 100-42-5 | 2.68 | 2.83 | -0.95 | 0.85 | 0.65 | 0 | 0.16 | 0.9552 | 3.856 |
| 230 | Octane | 111-65-9 | 5.27 | 4.90 | 1.98 | 0 | 0 | 0 | 0 | 1.2358 | 3.677 |
| 231 | 3-Chlorophenol | 108-43-0 | 1.66 | 2.42 | -4.87 | 0.91 | 1.06 | 0.69 | 0.15 | 0.8975 | 4.773 |
| 232 | Benzyl acetate | 140-11-4 | 1.70 | 1.69 | -4.09 | 0.8 | 0.85 | 0 | 0.65 | 1.2135 | 5.03 |
| 233 | 1-Naphthol | 90-15-3 | 2.19 | 2.87 | -5.65 | 1.52 | 1.1 | 0.66 | 0.34 | 1.1441 | 6.284 |
| 234 | 4-bromophenol | 106-41-2 | 1.61 | 2.38 | -5.31 | 1.08 | 1.17 | 0.67 | 0.19 | 0.9501 | 5.004 |
| 235 | 4-Iodoaniline | 540-37-4 | 2.19 | 2.14 | -5.14 | 1.53 | 1.28 | 0.31 | 0.3 | 1.0744 | 5.695 |
| 236 | N,N-Diethylaniline | 91-66-7 | 3.17 | 3.40 | -2.08 | 0.95 | 0.8 | 0 | 0.41 | 1.3798 | 5.287 |
| 237 | 4-n-Propylphenol | 645-56-7 | 2.19 | 2.84 | -4.49 | 0.79 | 0.88 | 0.54 | 0.35 | 1.1978 | 5.185 |
| 238 | 4-iodophenol | 540-38-5 | 2.29 | 2.66 | -5.41 | 1.38 | 1.22 | 0.68 | 0.2 | 1.0333 | 5.492 |
| 239 | 1,3-Dinitrobenzene | 99-65-0 | 1.42 | 1.57 | -5.13 | 1.15 | 1.6 | 0 | 0.47 | 1.0648 | 5.903 |
| 240 | Anthracene | 120-12-7 | 4.83 | 4.24 | -3.47 | 2.29 | 1.34 | 0 | 0.28 | 1.4544 | 7.568 |
| 241 | Phenanthrene | 85-01-8 | 4.80 | 4.41 | -3.30 | 2.06 | 1.29 | 0 | 0.29 | 1.4544 | 7.632 |
| 242 | Fluoranthene | 206-44-0 | 5.18 | 4.94 | -3.98 | 2.38 | 1.55 | 0 | 0.24 | 1.5846 | 8.827 |
| 243 | Pyrene | 129-00-0 | 5.26 | 4.58 | -4.51 | 2.81 | 1.71 | 0 | 0.28 | 1.5846 | 8.833 |
| 244 | Fluorene | 86-73-7 | 4.39 | 4.23 | -2.69 | 1.59 | 1.06 | 0 | 0.25 | 1.3565 | 6.922 |
| 245 | Acenaphthene | 83-32-9 | 3.97 | 3.92 | -2.55 | 1.6 | 1.05 | 0 | 0.22 | 1.2586 | 6.469 |
| 246 | phenol | 108-95-2 | -0.16 | 1.43 | -4.75 | 0.81 | 0.89 | 0.6 | 0.3 | 0.7751 | 3.766 |
| 247 | carbon disulfide | 75-15-0 | 2.46 | 2.07 | 0.02 | 0.88 | 0.26 | 0 | 0.03 | 0.4905 | 2.37 |
| 248 | acetylene | 74-86-2 | -0.63 | 0.25 | -0.32 | 0.19 | 0.47 | 0.12 | 0.05 | 0.3044 | 0.07 |
| 249 | methanal | 50-00-0 | -0.41 | -0.83 | -2.14 | 0.22 | 0.62 | 0 | 0.33 | 0.2652 | 0.73 |
| 250 | 1-Propanethiol | 107-03-9 | 1.93 | 1.85 | -0.82 | 0.39 | 0.42 | 0 | 0.21 | 0.6948 | 2.598 |
| 251 | 1-Butanethiol | 109-79-5 | 2.53 | 2.37 | -0.67 | 0.38 | 0.43 | 0 | 0.21 | 0.8357 | 3.059 |
| 252 | 1-Pentanethiol | 110-66-7 | 3.28 | 2.92 | -0.57 | 0.37 | 0.43 | 0 | 0.21 | 0.9766 | 3.624 |
| 253 | 1-Hexanethiol | 111-31-9 | 4.02 | 3.49 | -0.44 | 0.36 | 0.42 | 0 | 0.21 | 1.1175 | 4.133 |
| 254 | 2-Pentanol | 6032-29-7 | 0.30 | 1.16 | -3.23 | 0.2 | 0.36 | 0.33 | 0.56 | 0.8718 | 2.84 |
| 255 | 2-Hexanol | 626-93-7 | 0.91 | 1.72 | -3.11 | 0.19 | 0.36 | 0.33 | 0.56 | 1.0127 | 3.34 |
| 256 | 3-Hexanol | 623-37-0 | 0.48 | 1.72 | -3.12 | 0.2 | 0.36 | 0.33 | 0.56 | 1.0127 | 3.343 |
| 257 | 2-Heptanol | 543-49-7 | 1.52 | 2.27 | -2.99 | 0.19 | 0.36 | 0.33 | 0.56 | 1.1536 | 3.838 |
| 258 | 4-Heptanol | 589-55-9 | 1.67 | 2.28 | -3.00 | 0.18 | 0.36 | 0.33 | 0.56 | 1.1536 | 3.85 |
| 259 | 4-Octanol | 589-62-8 | 2.42 | 2.81 | -2.86 | 0.16 | 0.36 | 0.33 | 0.56 | 1.2945 | 4.3 |
| 260 | 2,2,2-Trifluoroethanol | 75-89-8 | -0.55 | 0.27 | -3.27 | 0.02 | 0.6 | 0.57 | 0.25 | 0.5022 | 1.224 |
| 261 | 1,1,1-Trifluoro-2-propanol | 374-01-6 | -0.05 | 0.76 | -2.80 | 0.11 | 0.47 | 0.37 | 0.36 | 0.6431 | 1.963 |
| 262 | 2,2,3,3-Tetrafluoro-1-propanol | 76-37-9 | -0.10 | 1.39 | -3.27 | 0.01 | 0.44 | 0.77 | 0.18 | 0.6608 | 1.949 |
| 263 | 2,2,3,3,3-Pentafluoro-1-propanol | 422-05-9 | 0.34 | 1.50 | -2.23 | -0.17 | 0.51 | 0.62 | 0.08 | 0.6785 | 1.777 |
| 264 | 1,1,1,3,3,3-Hexafluoro-2-propanol | 920-66-1 | 0.76 | 1.36 | -2.75 | -0.24 | 0.55 | 0.77 | 0.1 | 0.6962 | 1.392 |
| 265 | 1,1,1,3,3,3-Hexafluoro-2-methyl-2-propanol | 1515-14-6 | 1.10 | 2.16 | -1.63 | -0.19 | 0.25 | 0.54 | 0.18 | 0.8371 | 2.034 |
| 266 | 2,2,3,4,4,4-Hexafluoro-1-butanol | 382-31-0 | 0.66 | 1.99 | -2.80 | -0.22 | 0.54 | 0.71 | 0.14 | 0.8371 | 2.328 |
| 267 | 2,2,3,3,4,4,4-Heptafluoro-1-butanol | 375-01-9 | 1.17 | 2.48 | -1.60 | -0.3 | 0.18 | 0.69 | 0.11 | 0.8548 | 1.937 |
| 268 | 2-Butanol | 78-92-2 | -0.28 | 0.61 | -3.35 | 0.22 | 0.36 | 0.33 | 0.56 | 0.7309 | 2.338 |
| 269 | Triethyl phosphate | 78-40-0 | 0.22 | 0.67 | -5.37 | 0 | 1.12 | 0 | 1.05 | 1.3934 | 5.04 |
| 270 | 1,2-Dihydroxybenzene / Catechol | 120-80-9 | 0.79 | 0.81 | -7.29 | 0.97 | 1.1 | 0.88 | 0.47 | 0.8338 | 4.45 |
| 271 | PCB 28 | 7012-37-5 | 5.83 | 5.42 | -2.37 | 1.76 | 1.33 | 0 | 0.15 | 1.6914 | 7.904 |
| 272 | PCB 31 | 16606-02-3 | 5.83 | 5.40 | -2.35 | 1.77 | 1.33 | 0 | 0.15 | 1.6914 | 7.862 |
| 273 | PCB 44 | 41464-39-5 | 6.01 | 5.68 | -2.57 | 1.9 | 1.48 | 0 | 0.15 | 1.8138 | 8.312 |
| 274 | PCB 49 | 41464-40-8 | 6.03 | 5.63 | -2.51 | 1.89 | 1.48 | 0 | 0.15 | 1.8138 | 8.186 |
| 275 | PCB 52 | 35693-99-3 | 6.04 | 5.61 | -2.49 | 1.9 | 1.48 | 0 | 0.15 | 1.8138 | 8.144 |
| 276 | PCB 99 | 38380-01-7 | 6.52 | 6.12 | -2.72 | 2.03 | 1.61 | 0 | 0.13 | 1.9362 | 8.91 |
| 277 | PCB 101 | 37680-73-2 | 6.52 | 6.10 | -2.70 | 2.04 | 1.61 | 0 | 0.13 | 1.9362 | 8.868 |
| 278 | PCB 105 | 32598-14-4 | 6.82 | 6.51 | -2.90 | 2.04 | 1.59 | 0 | 0.11 | 1.9362 | 9.594 |
| 279 | PCB 110 | 38380-03-9 | 6.53 | 6.23 | -2.84 | 2.04 | 1.61 | 0 | 0.13 | 1.9362 | 9.161 |
| 280 | PCB 118 | 31508-00-6 | 6.81 | 6.43 | -2.81 | 2.06 | 1.59 | 0 | 0.11 | 1.9362 | 9.396 |
| 281 | PCB 128 | 38380-07-3 | 6.97 | 6.75 | -3.08 | 2.18 | 1.74 | 0 | 0.11 | 2.0586 | 9.957 |
| 282 | PCB 138 | 35065-28-2 | 7.00 | 6.67 | -2.99 | 2.18 | 1.74 | 0 | 0.11 | 2.0586 | 9.772 |
| 283 | PCB 149 | 38380-04-0 | 6.83 | 6.49 | -2.79 | 2.16 | 1.74 | 0 | 0.11 | 2.0586 | 9.352 |
| 284 | PCB 151 | 52663-63-5 | 6.77 | 6.40 | -2.69 | 2.17 | 1.74 | 0 | 0.11 | 2.0586 | 9.142 |
| 285 | PCB 153 | 35065-27-1 | 7.03 | 6.59 | -2.90 | 2.18 | 1.74 | 0 | 0.11 | 2.0586 | 9.587 |
| 286 | PCB 156 | 38380-08-4 | 7.34 | 6.95 | -3.06 | 2.21 | 1.72 | 0 | 0.09 | 2.0586 | 10.2 |
| 287 | PCB 170 | 35065-30-6 | 7.45 | 7.20 | -3.23 | 2.33 | 1.87 | 0 | 0.09 | 2.181 | 10.577 |
| 288 | PCB 180 | 35065-29-3 | 7.49 | 7.13 | -3.16 | 2.29 | 1.87 | 0 | 0.09 | 2.181 | 10.415 |
| 289 | PCB 187 | 52663-68-0 | 7.27 | 6.89 | -2.89 | 2.31 | 1.87 | 0 | 0.09 | 2.181 | 9.864 |
| 290 | PCB 188 | 74487-85-7 | 7.03 | 6.70 | -2.68 | 2.28 | 1.87 | 0 | 0.09 | 2.181 | 9.428 |
| 291 | PCB 194 | 35694-08-7 | 8.01 | 7.67 | -3.34 | 2.48 | 2 | 0 | 0.06 | 2.3034 | 11.186 |
| 292 | PCB 209 | 2051-24-3 | 8.26 | 8.26 | -3.31 | 2.72 | 2.26 | 0 | 0.02 | 2.5482 | 11.703 |
| 293 | Naphthalene | 91-20-3 | 3.61 | 3.19 | -2.00 | 1.34 | 0.92 | 0 | 0.2 | 1.0854 | 5.161 |
| 294 | Benz[a]anthracene | 56-55-3 | 6.26 | 5.62 | -4.83 | 2.99 | 1.7 | 0 | 0.35 | 1.8234 | 10.291 |
| 295 | Chrysene | 218-01-9 | 6.25 | 5.60 | -4.91 | 3.03 | 1.73 | 0 | 0.36 | 1.8234 | 10.334 |
| 296 | Benzo[a]pyrene | 50-32-8 | 6.79 | 5.78 | -6.30 | 3.63 | 1.96 | 0 | 0.37 | 1.9536 | 11.736 |
| 297 | Benzo[k]fluoranthene | 207-08-9 | 6.84 | 6.21 | -5.56 | 3.19 | 1.91 | 0 | 0.33 | 1.9536 | 11.607 |
| 298 | Teflurane | 124-72-1 | 1.80 | 2.13 | 0.39 | -0.06 | 0.3 | 0.09 | 0.04 | 0.6362 | 1.54 |
| 299 | 2-[Chloro(fluoro)methoxy]-1,1,1,2-tetrafluoroethane | 56885-28-0 | 2.15 | 1.99 | 0.17 | -0.24 | 0.5 | 0 | 0.11 | 0.8009 | 1.865 |
| 300 | 1,1,1,2,3,4,4,4-Octafluorobutane | 75995-72-1 | 2.44 | 2.29 | 1.00 | -0.79 | 0.2 | 0.15 | 0.07 | 0.8138 | 1.14 |
| 301 | 1,1,1,2,2,3,3,4,4-nonafluorobutane | 375-17-7 | 2.82 | 2.90 | 2.63 | -0.89 | -0.43 | 0.04 | 0.1 | 0.8315 | 0.7 |
| 302 | Sulfur hexafluoride | 2551-62-4 | 1.85 | 1.69 | 2.25 | -0.6 | -0.2 | 0 | 0 | 0.4643 | -0.12 |
| 303 | 2-(Chlorodifluoromethoxy)-1,1,1-trifluoroethane | 33018-78-9 | 2.73 | 2.58 | 1.39 | -0.24 | 0.15 | 0 | 0.04 | 0.8009 | 1.577 |
| 304 | 3,4-Dichlorophenol | 95-77-2 | 2.42 | 3.33 | -4.64 | 1.02 | 1.24 | 0.93 | 0 | 1.0199 | 5.48 |
| 305 | 1-Nitronaphthalene | 86-57-7 | 3.45 | 3.17 | -4.29 | 1.6 | 1.51 | 0 | 0.29 | 1.2596 | 7.056 |

## **Table S2**. Chemicals with their experimental values of logK_pw_ and predicted values of logK_ow_ and logK_aw_ used to calibrate phospholipid-water tp-LFER model

| **S. No** | **Chemicals** | **CAS-RN** | **logK_pw_** | **logK_ow_** | **logK_aw_** | **E** | **S** | **A** | **B** | **V** | **L** |
| --- | --- | --- | --- | --- | --- | --- | --- | --- | --- | --- | --- |
| 1 | n-hexane | 110-54-3 | 3.91 | 3.79 | 1.74 | 0 | 0 | 0 | 0 | 0.954 | 2.668 |
| 2 | n-heptane | 142-82-5 | 4.55 | 4.34 | 1.86 | 0 | 0 | 0 | 0 | 1.0949 | 3.173 |
| 3 | n-octane | 111-65-9 | 4.67 | 4.90 | 1.98 | 0 | 0 | 0 | 0 | 1.2358 | 3.677 |
| 4 | 2,2,4-trimethylpentane | 540-84-1 | 4.61 | 4.65 | 2.25 | 0 | 0 | 0 | 0 | 1.2358 | 3.106 |
| 5 | cyclohexane | 110-82-7 | 3.27 | 3.51 | 1.12 | 0.31 | 0.1 | 0 | 0 | 0.8454 | 2.964 |
| 6 | tetrachloromethane | 56-23-5 | 2.61 | 2.80 | 0.33 | 0.46 | 0.38 | 0 | 0 | 0.7391 | 2.823 |
| 7 | trichloroethene | 65386 | 2.43 | 2.71 | -0.23 | 0.52 | 0.37 | 0.08 | 0.03 | 0.7146 | 2.997 |
| 8 | tetrachloroethene | 127-18-4 | 3.08 | 3.28 | 0.09 | 0.64 | 0.44 | 0 | 0 | 0.837 | 3.584 |
| 9 | tribromomethane | 75-25-2 | 2.33 | 2.64 | -1.50 | 0.97 | 0.68 | 0.15 | 0.06 | 0.7745 | 3.784 |
| 10 | di-n-butyl ether | 142-96-1 | 2.78 | 3.24 | -0.70 | 0 | 0.25 | 0 | 0.45 | 1.2945 | 3.924 |
| 11 | di-n-pentyl ether | 693-65-2 | 3.77 | 4.33 | -0.44 | 0 | 0.25 | 0 | 0.45 | 1.5763 | 4.875 |
| 12 | acetone | 67-64-1 | 0.06 | -0.30 | -2.81 | 0.18 | 0.7 | 0.04 | 0.49 | 0.547 | 1.696 |
| 13 | 2-octanone | 111-13-7 | 2.42 | 2.47 | -2.15 | 0.11 | 0.68 | 0 | 0.51 | 1.2515 | 4.257 |
| 14 | 2-nonanone | 821-55-6 | 2.83 | 3.01 | -2.02 | 0.11 | 0.68 | 0 | 0.51 | 1.3924 | 4.735 |
| 15 | 2-decanone | 693-54-9 | 3.16 | 3.57 | -1.91 | 0.11 | 0.68 | 0 | 0.51 | 1.5333 | 5.245 |
| 16 | cyclopentanone | 120-92-3 | 0.30 | 0.45 | -3.43 | 0.37 | 0.86 | 0 | 0.52 | 0.7202 | 3.221 |
| 17 | cyclohexanone | 108-94-1 | 0.54 | 0.90 | -3.54 | 0.4 | 0.86 | 0 | 0.56 | 0.8611 | 3.792 |
| 18 | ethyl acetate | 141-78-6 | 0.46 | 0.71 | -2.09 | 0.11 | 0.62 | 0 | 0.45 | 0.7466 | 2.314 |
| 19 | propyl acetate | 109-60-4 | 1.01 | 1.29 | -1.93 | 0.09 | 0.6 | 0 | 0.45 | 0.8875 | 2.819 |
| 20 | methanol | 67-56-1 | -0.53 | -0.82 | -3.87 | 0.28 | 0.44 | 0.43 | 0.47 | 0.3082 | 0.97 |
| 21 | ethanol | 64-17-5 | -0.26 | -0.25 | -3.54 | 0.25 | 0.42 | 0.37 | 0.48 | 0.4491 | 1.485 |
| 22 | 1-propanol | 71-23-8 | 0.17 | 0.32 | -3.45 | 0.24 | 0.42 | 0.37 | 0.48 | 0.59 | 2.031 |
| 23 | 2-propanol | 67-63-0 | -0.04 | 0.02 | -3.44 | 0.21 | 0.36 | 0.33 | 0.56 | 0.59 | 1.764 |
| 24 | 1-butanol | 71-36-3 | 0.51 | 0.91 | -3.36 | 0.22 | 0.42 | 0.37 | 0.48 | 0.7309 | 2.601 |
| 25 | tert-butanol | 75-65-0 | 0.16 | 0.40 | -3.17 | 0.18 | 0.3 | 0.31 | 0.6 | 0.7309 | 1.963 |
| 26 | 1-pentanol | 71-41-0 | 1.08 | 1.46 | -3.24 | 0.22 | 0.42 | 0.37 | 0.48 | 0.8718 | 3.106 |
| 27 | 3-pentanol | 584-02-1 | 1.00 | 1.17 | -3.24 | 0.22 | 0.36 | 0.33 | 0.56 | 0.8718 | 2.86 |
| 28 | 1-hexanol | 111-27-3 | 1.88 | 2.02 | -3.13 | 0.21 | 0.42 | 0.37 | 0.48 | 1.0127 | 3.61 |
| 29 | 1-heptanol | 111-70-6 | 2.38 | 2.57 | -3.01 | 0.21 | 0.42 | 0.37 | 0.48 | 1.1536 | 4.115 |
| 30 | 4-heptanol | 589-55-9 | 1.70 | 2.28 | -3.00 | 0.18 | 0.36 | 0.33 | 0.56 | 1.1536 | 3.85 |
| 31 | 1-octanol | 111-87-5 | 2.66 | 3.13 | -2.89 | 0.2 | 0.42 | 0.37 | 0.48 | 1.2945 | 4.619 |
| 32 | cyclopentanol | 96-41-3 | 0.52 | 0.82 | -4.04 | 0.43 | 0.54 | 0.26 | 0.57 | 0.7632 | 3.173 |
| 33 | cyclohexanol | 108-93-0 | 1.01 | 1.35 | -3.98 | 0.46 | 0.59 | 0.29 | 0.59 | 0.9041 | 3.732 |
| 34 | cycloheptanol | 502-41-0 | 1.51 | 1.93 | -3.98 | 0.56 | 0.59 | 0.26 | 0.59 | 1.045 | 4.175 |
| 35 | ethylene glycol | 107-21-1 | -0.79 | -1.36 | -7.18 | 0.4 | 0.9 | 0.58 | 0.78 | 0.5078 | 2.661 |
| 36 | 2-butoxyethanol | 111-76-2 | 0.60 | 0.94 | -4.68 | 0.2 | 0.53 | 0.26 | 0.83 | 1.0714 | 3.656 |
| 37 | benzyl alcohol | 100-51-6 | 1.14 | 1.14 | -4.84 | 0.8 | 0.87 | 0.39 | 0.56 | 0.916 | 4.221 |
| 38 | p-xylene | 106-42-3 | 2.98 | 3.11 | -0.56 | 0.61 | 0.52 | 0 | 0.16 | 0.9982 | 3.839 |
| 39 | chlorobenzene | 108-90-7 | 2.91 | 2.78 | -0.71 | 0.72 | 0.65 | 0 | 0.07 | 0.8388 | 3.657 |
| 40 | 1,2-dichlorobenzene | 95-50-1 | 3.64 | 3.36 | -0.94 | 0.87 | 0.78 | 0 | 0.04 | 0.9612 | 4.518 |
| 41 | 1,3-dichlorobenzene | 541-73-1 | 3.71 | 3.45 | -0.68 | 0.85 | 0.73 | 0 | 0.02 | 0.9612 | 4.41 |
| 42 | 1,4-dichlorobenzene | 106-46-7 | 3.57 | 3.44 | -0.74 | 0.83 | 0.75 | 0 | 0.02 | 0.9612 | 4.435 |
| 43 | 1,2,3-trichlorobenzene | 87-61-6 | 4.19 | 4.07 | -1.03 | 1.03 | 0.86 | 0 | 0 | 1.0836 | 5.419 |
| 44 | 1,2,4-trichlorobenzene | 120-82-1 | 4.20 | 4.07 | -0.84 | 0.98 | 0.81 | 0 | 0 | 1.0836 | 5.248 |
| 45 | 1,3,5-trichlorobenzene | 108-70-3 | 4.16 | 4.09 | -0.58 | 0.98 | 0.73 | 0 | 0 | 1.0836 | 5.045 |
| 46 | 1,2,3,5-tetrachlorobenzene | 634-90-2 | 4.77 | 4.59 | -0.94 | 1.16 | 0.85 | 0 | 0 | 1.206 | 5.922 |
| 47 | 1,2,4,5-tetrachlorobenzene | 95-94-3 | 4.73 | 4.58 | -0.96 | 1.16 | 0.86 | 0 | 0 | 1.206 | 5.926 |
| 48 | pentachlorobenzene | 608-93-5 | 5.18 | 5.08 | -1.33 | 1.33 | 0.92 | 0.06 | 0 | 1.3284 | 6.633 |
| 49 | hexachlorobenzene | 118-74-1 | 5.64 | 5.62 | -1.31 | 1.49 | 0.75 | 0 | 0.09 | 1.4508 | 6.986 |
| 50 | 2,4,5-trichlorotoluene | 6639-30-1 | 4.72 | 4.59 | -0.83 | 1.06 | 0.86 | 0 | 0 | 1.2245 | 5.805 |
| 51 | 1,4-dibromobenzene | 106-37-6 | 4.30 | 3.85 | -1.22 | 1.15 | 0.86 | 0 | 0.04 | 1.0664 | 5.324 |
| 52 | 2 ,2',4,6-tetrachlorobiphenyl (PCB 50) | 62796-65-0 | 5.92 | 5.48 | -2.35 | 1.86 | 1.48 | 0 | 0.15 | 1.8138 | 7.765 |
| 53 | 2 ,2',5,5'-tetrachlorobiphenyl (PCB 52) | 35693-99-3 | 5.94 | 5.61 | -2.49 | 1.9 | 1.48 | 0 | 0.15 | 1.8138 | 8.144 |
| 54 | 3 ,3',4,5-tetrachlorobiphenyl (PCB 78) | 70362-49-1 | 6.53 | 6.19 | -2.64 | 1.94 | 1.44 | 0 | 0.11 | 1.8138 | 9.039 |
| 55 | 2 ,2',4,5',6-pentachlorobiphenyl (PCB 103) | 60145-21-3 | 6.32 | 5.91 | -2.48 | 2.01 | 1.61 | 0 | 0.13 | 1.9362 | 8.429 |
| 56 | 2 ,2',4,6,6'-pentachlorobiphenyl (PCB 104) | 56558-16-8 | 6.13 | 5.83 | -2.40 | 1.98 | 1.61 | 0 | 0.13 | 1.9362 | 8.244 |
| 57 | 2 ,2',3,3',6,6'-hexachlorobiphenyl (PCB 136) | 38411-22-2 | 6.50 | 6.39 | -2.67 | 2.14 | 1.74 | 0 | 0.11 | 2.0586 | 9.117 |
| 58 | 2 ,2',3,4,4',5,6'-heptachlorobiphenyl (PCB 182) | 60145-23-5 | 6.83 | 6.93 | -2.94 | 2.3 | 1.87 | 0 | 0.09 | 2.181 | 9.958 |
| 59 | phenanthrene | 67580 | 4.95 | 4.41 | -3.30 | 2.06 | 1.29 | 0 | 0.29 | 1.4544 | 7.632 |
| 60 | anthracene | 120-12-7 | 5.21 | 4.24 | -3.47 | 2.29 | 1.34 | 0 | 0.28 | 1.4544 | 7.568 |
| 61 | fluoranthene | 206-44-0 | 5.58 | 4.94 | -3.98 | 2.38 | 1.55 | 0 | 0.24 | 1.5846 | 8.827 |
| 62 | pyrene | 129-00-0 | 5.71 | 4.58 | -4.51 | 2.81 | 1.71 | 0 | 0.28 | 1.5846 | 8.833 |
| 63 | benzo[a]anthracene | 56-55-3 | 6.44 | 5.62 | -4.83 | 2.99 | 1.7 | 0 | 0.35 | 1.8234 | 10.291 |
| 64 | chrysene | 218-01-9 | 6.40 | 5.60 | -4.91 | 3.03 | 1.73 | 0 | 0.36 | 1.8234 | 10.334 |
| 65 | benzo[b]fluoranthene | 205-99-2 | 7.11 | 6.10 | -5.73 | 3.19 | 1.82 | 0 | 0.4 | 1.9536 | 11.632 |
| 66 | benzo[k]fluoranthene | 207-08-9 | 7.13 | 6.21 | -5.56 | 3.19 | 1.91 | 0 | 0.33 | 1.9536 | 11.607 |
| 67 | benzo[a]pyrene | 50-32-8 | 7.19 | 5.78 | -6.30 | 3.63 | 1.96 | 0 | 0.37 | 1.9536 | 11.736 |
| 68 | benzo[ghi]perylene | 191-24-2 | 7.78 | 6.83 | -6.59 | 4.07 | 1.9 | 0 | 0.45 | 2.0838 | 13.447 |
| 69 | dibenz[a,h]anthracene | 53-70-3 | 7.72 | 6.80 | -6.41 | 4 | 2.04 | 0 | 0.44 | 2.1924 | 12.96 |
| 70 | dibenz[a,c]anthracene | 215-58-7 | 7.49 | 6.97 | -6.20 | 4 | 1.93 | 0 | 0.44 | 2.1924 | 12.998 |
| 71 | indeno[1,2,3-cd]pyrene | 193-39-5 | 7.86 | 6.65 | -6.23 | 3.61 | 1.93 | 0 | 0.42 | 2.0838 | 12.699 |
| 72 | nitrobenzene | 98-95-3 | 2.01 | 1.91 | -2.99 | 0.87 | 1.11 | 0 | 0.28 | 0.8906 | 4.557 |
| 73 | 2-nitrotoluene | 88-72-2 | 2.41 | 2.39 | -2.78 | 0.87 | 1.11 | 0 | 0.28 | 1.0315 | 4.878 |
| 74 | quinoline | 91-22-5 | 1.67 | 1.97 | -4.00 | 1.27 | 0.97 | 0 | 0.54 | 1.0443 | 5.457 |
| 75 | Diethyl phthalate | 84-66-2 | 1.77 | 2.37 | -5.49 | 0.73 | 1.26 | 0 | 0.9 | 1.7106 | 7.214 |
| 76 | Dibutyl phthalate | 84-74-2 | 3.87 | 4.58 | -4.83 | 0.7 | 1.27 | 0 | 0.95 | 2.2742 | 8.97 |
| 77 | phenol | 108-95-2 | 1.96 | 1.43 | -4.75 | 0.81 | 0.89 | 0.6 | 0.3 | 0.7751 | 3.766 |
| 78 | 2-methylphenol | 95-48-7 | 2.45 | 1.99 | -4.30 | 0.84 | 0.86 | 0.52 | 0.3 | 0.916 | 4.218 |
| 79 | 3-methylphenol | 108-39-4 | 2.34 | 1.88 | -4.71 | 0.82 | 0.88 | 0.57 | 0.34 | 0.916 | 4.31 |
| 80 | 4-methylphenol | 106-44-5 | 2.35 | 1.97 | -4.60 | 0.82 | 0.87 | 0.57 | 0.31 | 0.916 | 4.312 |
| 81 | 2-ethylphenol | 90-00-6 | 2.81 | 2.32 | -4.38 | 0.83 | 0.84 | 0.52 | 0.37 | 1.0569 | 4.612 |
| 82 | 4-ethylphenol | 123-07-9 | 2.78 | 2.31 | -4.62 | 0.8 | 0.9 | 0.55 | 0.36 | 1.0569 | 4.737 |
| 83 | 2,6-dimethylphenol | 576-26-1 | 2.47 | 2.37 | -3.93 | 0.84 | 0.79 | 0.39 | 0.38 | 1.0569 | 4.68 |
| 84 | 2-n-propylphenol | 644-35-9 | 3.13 | 2.77 | -4.22 | 0.82 | 0.84 | 0.52 | 0.37 | 1.1978 | 5.248 |
| 85 | 4-n-propylphenol | 645-56-7 | 2.92 | 2.84 | -4.49 | 0.79 | 0.88 | 0.54 | 0.35 | 1.1978 | 5.185 |
| 86 | 4-isopropylphenol | 99-89-8 | 3.25 | 2.70 | -4.46 | 0.79 | 0.89 | 0.55 | 0.35 | 1.1978 | 4.984 |
| 87 | 3,4,5-trimethylphenol | 527-54-8 | 2.66 | 2.76 | -5.01 | 0.83 | 0.89 | 0.58 | 0.42 | 1.1978 | 5.568 |
| 88 | 4-n-butylphenol | 1638-22-8 | 3.13 | 3.38 | -4.35 | 0.8 | 0.88 | 0.55 | 0.37 | 1.3387 | 5.75 |
| 89 | 2-sec-butylphenol | 89-72-5 | 3.47 | 2.95 | -4.21 | 0.82 | 0.91 | 0.52 | 0.41 | 1.3387 | 5.87 |
| 90 | 2-tert-butylphenol | 88-18-6 | 3.51 | 2.95 | -4.17 | 0.82 | 0.92 | 0.52 | 0.4 | 1.3387 | 5.903 |
| 91 | 4-tert-butylphenol | 98-54-4 | 3.48 | 3.13 | -4.32 | 0.81 | 0.91 | 0.56 | 0.4 | 1.3387 | 5.876 |
| 92 | 4-tert-amylphenol | 80-46-6 | 3.54 | 3.62 | -4.30 | 0.81 | 0.89 | 0.56 | 0.42 | 1.4796 | 6.2 |
| 93 | 2-phenylphenol | 90-43-7 | 3.43 | 3.02 | -6.69 | 1.4 | 1.31 | 0.52 | 0.49 | 1.3829 | 7.227 |
| 94 | 4-phenylphenol | 92-69-3 | 3.52 | 3.06 | -6.54 | 1.4 | 1.31 | 0.61 | 0.42 | 1.3829 | 7.23 |
| 95 | bisphenol A | 65873 | 3.92 | 3.44 | -10.56 | 1.61 | 1.49 | 1.01 | 0.91 | 1.8643 | 9.239 |
| 96 | 2-chlorophenol | 95-57-8 | 2.76 | 1.93 | -3.63 | 0.85 | 0.88 | 0.32 | 0.31 | 0.8975 | 4.178 |
| 97 | 3-chlorophenol | 108-43-0 | 2.78 | 2.42 | -4.87 | 0.91 | 1.06 | 0.69 | 0.15 | 0.8975 | 4.773 |
| 98 | 4-chlorophenol | 106-48-9 | 2.73 | 2.19 | -5.13 | 0.92 | 1.08 | 0.67 | 0.2 | 0.8975 | 4.775 |
| 99 | 4-chloro-3-methylphenol | 59-50-7 | 3.32 | 2.77 | -4.92 | 0.92 | 0.99 | 0.67 | 0.22 | 1.0384 | 5.081 |
| 100 | 2,4-dichlorophenol | 120-83-2 | 3.57 | 3.06 | -3.67 | 0.96 | 0.82 | 0.54 | 0.17 | 1.0199 | 4.896 |
| 101 | 2,6-dichlorophenol | 87-65-0 | 2.86 | 2.82 | -3.68 | 0.9 | 0.86 | 0.36 | 0.24 | 1.0199 | 4.777 |
| 102 | 3,4-dichlorophenol | 95-77-2 | 3.76 | 3.43 | -4.75 | 1.02 | 1.24 | 0.93 | 0 | 1.0199 | 5.48 |
| 103 | 2,4,5-trichlorophenol | 95-95-4 | 4.46 | 3.78 | -4.32 | 1.07 | 0.92 | 0.73 | 0.1 | 1.1423 | 5.665 |
| 104 | 2,4,6-trichlorophenol | 68821 | 3.80 | 3.76 | -4.10 | 1.01 | 0.8 | 0.6 | 0.15 | 1.1423 | 5.67 |
| 105 | 3,4,5-trichlorophenol | 609-19-8 | 4.71 | 4.35 | -5.08 | 1.13 | 1.15 | 0.99 | 0 | 1.1423 | 6.464 |
| 106 | 2,3,4,5-tetrachlorophenol | 4901-51-3 | 4.76 | 4.30 | -4.26 | 1.17 | 0.88 | 0.7 | 0.13 | 1.2647 | 6.353 |
| 107 | pentachlorophenol | 87-86-5 | 5.10 | 5.27 | -3.92 | 1.22 | 0.91 | 0.66 | 0.06 | 1.3871 | 6.805 |
| 108 | 4-fluorophenol | 371-41-5 | 2.19 | 1.63 | -4.67 | 0.67 | 0.97 | 0.63 | 0.23 | 0.7928 | 3.844 |
| 109 | 4-bromophenol | 106-41-2 | 2.40 | 2.38 | -5.31 | 1.08 | 1.17 | 0.67 | 0.19 | 0.9501 | 5.004 |
| 110 | 4-iodophenol | 540-38-5 | 2.55 | 2.66 | -5.41 | 1.38 | 1.22 | 0.68 | 0.2 | 1.0333 | 5.492 |
| 111 | 2-nitrophenol | 88-75-5 | 1.89 | 1.91 | -3.43 | 1.02 | 1.05 | 0.05 | 0.37 | 0.9493 | 4.76 |
| 112 | 3-nitrophenol | 554-84-7 | 2.56 | 1.93 | -6.99 | 1.05 | 1.57 | 0.79 | 0.23 | 0.9493 | 5.692 |
| 113 | 4-nitrophenol | 100-02-7 | 2.72 | 1.68 | -7.65 | 1.07 | 1.72 | 0.82 | 0.26 | 0.9493 | 5.876 |
| 114 | 2,4-dinitrophenol | 51-28-5 | 2.67 | 1.57 | -5.56 | 1.2 | 1.49 | 0.09 | 0.56 | 1.1235 | 5.981 |
| 115 | 2,6-dinitrophenol | 573-56-8 | 2.03 | 1.15 | -6.70 | 1.22 | 2.04 | 0.17 | 0.48 | 1.1235 | 6.631 |
| 116 | 3,4-dinitrophenol | 577-71-9 | 3.17 | 1.68 | -9.02 | 1.32 | 2.25 | 1.14 | 0.16 | 1.1235 | 7.111 |
| 117 | 2-sec-butyl-4,6-dinitrophenol | 88-85-7 | 3.73 | 3.66 | -5.32 | 1.25 | 1.72 | 0.28 | 0.52 | 1.6871 | 8.346 |
| 118 | 4-cyanophenol | 767-00-0 | 2.11 | 1.44 | -7.38 | 0.94 | 1.63 | 0.8 | 0.29 | 0.9298 | 5.42 |
| 119 | aniline | 62-53-3 | 1.63 | 1.18 | -4.16 | 0.96 | 0.96 | 0.26 | 0.41 | 0.8162 | 3.934 |
| 120 | 3,4-dimethylaniline | 95-64-7 | 2.11 | 2.08 | -4.18 | 0.96 | 0.97 | 0.2 | 0.49 | 1.098 | 5.089 |
| 121 | 3-nitroaniline | 72930 | 2.17 | 1.56 | -6.42 | 1.2 | 1.71 | 0.4 | 0.35 | 0.9904 | 5.88 |
| 122 | N,N-dimethylaniline | 121-69-7 | 2.33 | 2.37 | -2.65 | 0.96 | 0.81 | 0 | 0.41 | 1.098 | 4.701 |
| 123 | estrone | 53-16-7 | 3.59 | 3.46 | -10.42 | 1.73 | 2.05 | 0.5 | 1.08 | 2.1558 | 10.78 |
| 124 | -estradiol | 50-28-2 | 3.33 | 3.97 | -11.31 | 1.8 | 1.77 | 0.86 | 1.1 | 2.1988 | 11.1 |
| 125 | estriol | 50-27-1 | 1.96 | 1.50 | -17.17 | 1.97 | 1.74 | 1.06 | 1.63 | 2.2575 | 11.7 |
| 126 | progesterone | 57-83-0 | 3.28 | 3.27 | -10.87 | 1.45 | 3.29 | 0 | 1.14 | 2.6215 | 13.27 |
| 127 | diazepam | 439-14-5 | 2.99 | 3.24 | -8.59 | 2.17 | 1.78 | 0 | 1.27 | 2.0739 | 11.01 |
| 128 | lidocaine | 137-58-6 | 2.15 | 2.57 | -7.52 | 1.11 | 1.51 | 0.07 | 1.24 | 2.0589 | 9.127 |
| 129 | diclofenac | 15307-86-5 | 4.45 | 4.60 | -9.14 | 1.81 | 1.85 | 0.55 | 0.77 | 2.025 | 11.025 |
| 130 | ibuprofen | 15687-27-1 | 3.80 | 3.90 | -5.68 | 0.73 | 0.7 | 0.57 | 0.79 | 1.7771 | 7.184 |
| 131 | salicylic acid | 69-72-7 | 2.55 | 2.16 | -5.40 | 0.9 | 0.85 | 0.73 | 0.37 | 0.9904 | 4.732 |

## **Table S3**. Showing chemicals having experimental values of logK_ow_ and logK_aw_ from the logK_lw_ dataset.

| **S. No** | **Chemicals** | **CAS-RN** | **SMILES** | **K_ow_-experimental** | **K_aw_-experimental** |
| --- | --- | --- | --- | --- | --- |
| 1 | 1,1,1-Trichloroethane | 71-55-6 | CC(Cl)(Cl)Cl | 2.49 | -0.14 |
| 2 | 1,1,2,2-Tetrachloroethane | 79-34-5 | ClC(C(Cl)Cl)Cl | 2.39 | -1.82 |
| 3 | 1,1,2-Trichloroethane | 79-00-5 | ClCC(Cl)Cl | 1.89 | -1.46 |
| 4 | 1,1-Dichloroethane | 75-34-3 | CC(Cl)Cl | 1.79 | -0.63 |
| 5 | 1,1-Dichloroethene | 75-35-4 | ClC(=C)Cl | 2.13 | 0.04 |
| 6 | 1,2,3-Trimethylbenzene | 526-73-8 | Cc1c(C)cccc1C | 3.66 | -0.74 |
| 7 | 1,2,4-Trimethylbenzene | 95-63-6 | Cc1ccc(c(c1)C)C | 3.63 | -0.59 |
| 8 | 1,2-Dibromoethane | 106-93-4 | BrCCBr | 1.96 | -1.57 |
| 9 | 1,2-Dichlorobenzene | 95-50-1 | Clc1ccccc1Cl | 3.43 | -1.10 |
| 10 | 1,2-Dichloroethane | 107-06-2 | ClCCCl | 1.48 | -1.31 |
| 11 | 1,2-Dichloropropane | 78-87-5 | ClCC(Cl)C | 1.98 | -0.93 |
| 12 | 1,3,5-Trimethylbenzene | 108-67-8 | Cc1cc(C)cc(c1)C | 3.42 | -0.44 |
| 13 | 1,3-Dichlorobenzene | 541-73-1 | Clc1cccc(c1)Cl | 3.53 | -0.96 |
| 14 | 1-Butanol | 71-36-3 | CCCCO | 0.88 | -3.44 |
| 15 | 1-Chlorobutane | 109-69-3 | CCCCCl | 2.64 | -0.16 |
| 16 | 1-Chloropentane | 543-59-9 | CCCCCCl | 2.73 | 0.00 |
| 17 | 1-Chloropropane | 540-54-5 | CCCCl | 2.04 | -0.26 |
| 18 | 1-Hexanol | 111-27-3 | CCCCCCO | 2.03 | -3.15 |
| 19 | 1-Nitropropane | 108-03-2 | CCCN(=O)=O | 0.87 | -2.44 |
| 20 | 1-Pentanol | 71-41-0 | CCCCCO | 1.51 | -3.27 |
| 21 | 1-Propanol | 71-23-8 | CCCO | 0.25 | -3.51 |
| 22 | 2,2-Dimethylbutane | 75-83-2 | CCC(C)(C)C | 3.82 | 1.80 |
| 23 | 2-Butoxyethanol | 111-76-2 | CCCCOCCO | 0.83 | -4.18 |
| 24 | 2-Chloropropane | 75-29-6 | CC(Cl)C | 1.90 | -0.14 |
| 25 | 2-Ethoxyethanol | 110-80-5 | OCCOCC | -0.32 | -4.71 |
| 26 | 2-Heptanone | 110-43-0 | CCCCCC(=O)C | 1.98 | -2.15 |
| 27 | 2-Hexanone | 591-78-6 | CCCCC(=O)C | 1.38 | -2.41 |
| 28 | 2-Isopropoxyethanol | 109-59-1 | OCCOC(C)C | 0.05 | -4.42 |
| 29 | 2-Methoxyethanol | 109-86-4 | COCCO | -0.77 | -4.86 |
| 30 | 2-Methyl-1-propanol | 78-83-1 | OCC(C)C | 0.76 | -3.39 |
| 31 | 2-Methyl-2-propanol | 75-65-0 | CC(O)(C)C | 0.35 | -3.42 |
| 32 | 2-Nitropropane | 79-46-9 | CC(N(=O)=O)C | 0.93 | -2.30 |
| 33 | 2-Pentanone | 107-87-9 | CCCC(=O)C | 0.91 | -2.46 |
| 34 | 2-Propanol | 67-63-0 | CC(O)C | 0.05 | -3.47 |
| 35 | 3-Methyl-1-butanol | 137-32-6 | CCC(CO)C | 1.29 | -3.23 |
| 36 | 3-Methylpentane | 96-14-0 | CCC(CC)C | 3.60 | 1.85 |
| 37 | 3-Pentanone | 96-22-0 | CCC(=O)CC | 0.99 | -2.68 |
| 38 | 4-Methyl-2-pentanone | 108-10-1 | CC(CC(=O)C)C | 1.31 | -2.24 |
| 39 | Acetone | 67-64-1 | CC(=O)C | -0.24 | -2.84 |
| 40 | Benzene | 71-43-2 | c1ccccc1 | 2.13 | -0.64 |
| 41 | Bromochloromethane | 74-97-5 | ClCBr | 1.41 | -1.22 |
| 42 | 2-Butanone | 78-93-3 | CCC(=O)C | 0.29 | -2.63 |
| 43 | Butane | 106-97-8 | CCCC | 2.89 | 1.60 |
| 44 | Butyl acetate | 123-86-4 | CCCCOC(=O)C | 1.78 | -1.93 |
| 45 | Carbon tetrachloride | 56-23-5 | ClC(Cl)(Cl)Cl | 2.83 | 0.06 |
| 46 | 1,2-Dichlorotetrafluoroethane | 76-14-2 | FC(C(Cl)(F)F)(Cl)F | 2.82 | 2.07 |
| 47 | 1,1-Difluoroethane | 75-37-6 | CC(F)F | 0.75 | -0.07 |
| 48 | Halothane | 151-67-7 | ClC(C(F)(F)F)Br | 2.30 | -0.07 |
| 49 | Carbon tetrafluoride | 75-73-0 | C(F)(F)(F)F | 1.18 | 2.33 |
| 50 | Isoflurane | 26675-46-7 | FC(OC(C(F)(F)F)Cl)F | 2.06 | 0.08 |
| 51 | Chlorobenzene | 108-90-7 | Clc1ccccc1 | 2.84 | -0.89 |
| 52 | Chlorodibromomethane | 124-48-1 | ClC(Br)Br | 2.16 | -1.49 |
| 53 | Chloroethane | 75-00-3 | CCCl | 1.43 | -0.33 |
| 54 | Chloroform | 67-66-3 | ClC(Cl)Cl | 1.97 | -0.82 |
| 55 | cis-1,2-Dichloroethene | 156-59-2 | Cl/C=C\Cl | 2.00 | -0.77 |
| 56 | Cycloheptane | 291-64-5 | C1CCCCCC1 | 4.00 | 0.59 |
| 57 | Cyclohexane | 110-82-7 | C1CCCCC1 | 3.44 | 0.80 |
| 58 | Cyclopentane | 287-92-3 | C1CCCC1 | 3.00 | 0.80 |
| 59 | Cyclopropane | 75-19-4 | C1CC1 | 1.72 | 1.52 |
| 60 | Decane | 124-18-5 | CCCCCCCCCC | 5.01 | 2.33 |
| 61 | Dibromethane | 74-95-3 | BrCBr | 1.70 | -1.47 |
| 62 | Dichloromethane | 75-09-2 | ClCCl | 1.25 | -0.87 |
| 63 | Diethyl ether | 60-29-7 | CCOCC | 0.89 | -1.29 |
| 64 | Ethane | 74-84-0 | CC | 1.81 | 1.32 |
| 65 | Ethanol | 64-17-5 | CCO | -0.31 | -3.68 |
| 66 | Ethene | 74-85-1 | C=C | 1.13 | 0.98 |
| 67 | Ethyl acetate | 141-78-6 | CCOC(=O)C | 0.73 | -2.25 |
| 68 | Ethylbenzene | 100-41-4 | CCc1ccccc1 | 3.15 | -0.48 |
| 69 | Fluorobenzene | 462-06-6 | Fc1ccccc1 | 2.27 | -0.58 |
| 70 | Fluorochloromethane | 593-70-4 | FCCl | 0.51 | -0.56 |
| 71 | Heptane | 142-82-5 | CCCCCCC | 4.66 | 1.92 |
| 72 | Hexane | 110-54-3 | CCCCCC | 3.90 | 1.88 |
| 73 | Isobutyl acetate | 110-19-0 | CC(COC(=O)C)C | 1.78 | -1.72 |
| 74 | Isopentyl acetate | 123-92-2 | CC(CCOC(=O)C)C | 2.25 | -1.61 |
| 75 | Isopropylbenzene | 98-82-8 | CC(c1ccccc1)C | 3.66 | -0.32 |
| 76 | Methoxyflurane | 76-38-0 | COC(C(Cl)Cl)(F)F | 2.21 | -0.81 |
| 77 | Methane | 74-82-8 | C | 1.09 | 1.44 |
| 78 | Methanol | 67-56-1 | CO | -0.77 | -3.72 |
| 79 | Methyl acetate | 79-20-9 | COC(=O)C | 0.18 | -2.32 |
| 80 | Methyl chloride | 74-87-3 | CCl | 0.91 | -0.43 |
| 81 | Methylcyclopentane | 96-37-7 | CC1CCCC1 | 3.37 | 1.18 |
| 82 | m-Xylene | 108-38-3 | Cc1cccc(c1)C | 3.20 | -0.52 |
| 83 | Nonane | 111-84-2 | CCCCCCCCC | 5.65 | 2.15 |
| 84 | o-Xylene | 95-47-6 | Cc1ccccc1C | 3.16 | -0.67 |
| 85 | p-Xylene | 106-42-3 | Cc1ccc(cc1)C | 3.15 | -0.54 |
| 86 | Pentachloroethane | 76-01-7 | ClC(C(Cl)(Cl)Cl)Cl | 3.22 | -1.09 |
| 87 | Pentane | 109-66-0 | CCCCC | 3.39 | 1.72 |
| 88 | Pentyl acetate | 628-63-7 | CCCCCOC(=O)C | 2.30 | -1.79 |
| 89 | Propane | 74-98-6 | CCC | 2.36 | 1.47 |
| 90 | Propyl acetate | 109-60-4 | CCCOC(=O)C | 1.24 | -2.04 |
| 91 | Propylbenzene | 103-65-1 | CCCc1ccccc1 | 3.69 | -0.36 |
| 92 | Tetrachloroethene | 127-18-4 | ClC(=C(Cl)Cl)Cl | 3.40 | -0.13 |
| 93 | Toluene | 108-88-3 | Cc1ccccc1 | 2.73 | -0.56 |
| 94 | trans-1,2-Dichloroethene | 156-60-5 | Cl/C=C/Cl | 2.00 | -0.77 |
| 95 | Trichloroethene | 79-01-6 | ClC=C(Cl)Cl | 2.42 | -0.39 |
| 96 | triethylamine | 121-44-8 | CCN(CC)CC | 1.45 | -2.21 |
| 97 | alpha-pinene | 80-56-8 | CC1=CCC2CC1C2(C)C | 4.44 | 1.09 |
| 98 | 1,4-dioxane | 123-91-1 | O1CCOCC1 | -0.27 | -3.70 |
| 99 | 1-heptanol | 111-70-6 | CCCCCCCO | 2.62 | -3.11 |
| 100 | 2-methylpyridine | 109-06-8 | Cc1ccccn1 | 1.11 | -3.38 |
| 101 | 3-methylpyridine | 108-99-6 | Cc1cccnc1 | 1.20 | -3.49 |
| 102 | 4-methylpyridine | 108-89-4 | Cc1ccncc1 | 1.22 | -3.60 |
| 103 | benzyl alcohol | 100-51-6 | OCc1ccccc1 | 1.10 | -4.85 |
| 104 | bromobenzene | 108-86-1 | Brc1ccccc1 | 2.99 | -0.99 |
| 105 | butylbenzene | 104-51-8 | CCCCc1ccccc1 | 4.38 | -0.18 |
| 106 | cyclohexene | 110-83-8 | C1CCC=CC1 | 2.86 | 0.28 |
| 107 | cyclopentanone | 120-92-3 | O=C1CCCC1 | 0.38 | -3.38 |
| 108 | difluorochloromethane | 75-45-6 | FC(Cl)F | 1.08 | 0.23 |
| 109 | diisopropyl ether | 108-20-3 | CC(OC(C)C)C | 1.52 | -0.97 |
| 110 | dimethoxymethane | 109-87-5 | COCOC | 0.00 | -2.14 |
| 111 | dimethyl ether | 115-10-6 | COC | 0.10 | -1.38 |
| 112 | dimethylacetamide | 127-19-5 | CC(=O)N(C)C | -0.77 | -6.26 |
| 113 | dimethylformamide | 68-12-2 | O=CN(C)C | -1.01 | -5.51 |
| 114 | di-n-butyl ether | 142-96-1 | CCCCOCCCC | 3.21 | -0.60 |
| 115 | dodecane | 112-40-3 | CCCCCCCCCCCC | 6.10 | 2.53 |
| 116 | ethyl formate | 109-94-4 | CCOC=O | 0.23 | -1.79 |
| 117 | ethyl propanoate | 105-37-3 | CCOC(=O)CC | 1.21 | -1.98 |
| 118 | iodoethane | 75-03-6 | CCI | 2.00 | -0.54 |
| 119 | fluorotrichloromethane | 75-69-4 | FC(Cl)(Cl)Cl | 2.53 | 0.61 |
| 120 | methyl formate | 107-31-3 | COC=O | 0.03 | -2.03 |
| 121 | methylcyclohexane | 108-87-2 | CC1CCCCC1 | 3.61 | 1.25 |
| 122 | nitroethane | 79-24-3 | CCN(=O)=O | 0.18 | -2.70 |
| 123 | nitromethane | 75-52-5 | CN(=O)=O | -0.35 | -2.92 |
| 124 | N,N-dimethylaniline | 121-69-7 | CN(c1ccccc1)C | 2.31 | -2.63 |
| 125 | piperidine | 110-89-4 | C1CCCNC1 | 0.84 | -3.73 |
| 126 | propyl bromide | 106-94-5 | CCCBr | 2.10 | -0.52 |
| 127 | propyl formate | 110-74-7 | CCCOC=O | 0.83 | -1.74 |
| 128 | pyridine | 110-86-1 | c1cccnc1 | 0.65 | -3.34 |
| 129 | tetradecane | 629-59-4 | CCCCCCCCCCCCCC | 7.20 | 2.58 |
| 130 | tetrahydrofuran | 109-99-9 | C1CCCO1 | 0.46 | -2.53 |
| 131 | Octan-1-ol | 111-87-5 | CCCCCCCCO | 3.00 | -2.99 |
| 132 | Nonan-1-ol | 143-08-8 | CCCCCCCCCO | 3.77 | -2.89 |
| 133 | Hexanal | 66-25-1 | CCCCCC=O | 1.78 | -2.05 |
| 134 | 1-Hexene | 592-41-6 | CCCCC=C | 3.39 | 1.23 |
| 135 | 1-Heptene | 592-76-7 | CCCCCC=C | 3.99 | 1.24 |
| 136 | 1-Octene | 111-66-0 | CCCCCCC=C | 4.57 | 1.42 |
| 137 | 1-Nonene | 124-11-8 | CCCCCCCC=C | 5.15 | 1.52 |
| 138 | 1-Decene | 872-05-9 | CCCCCCCCC=C | 5.70 | 1.35 |
| 139 | 1,2,4-Trichlorobenzene | 120-82-1 | Clc1ccc(c(c1)Cl)Cl | 4.02 | -1.23 |
| 140 | Di-n-propyl ether | 111-43-3 | CCCOCCC | 2.03 | -1.04 |
| 141 | 2-octanone | 111-13-7 | CCCCCCC(=O)C | 2.37 | -2.11 |
| 142 | 2-nonanone | 821-55-6 | CCCCCCCC(=O)C | 3.14 | -1.82 |
| 143 | 1-Nitrobutane | 627-05-4 | CCCCN(=O)=O | 1.47 | -2.30 |
| 144 | 4-Ethylpyridine | 536-75-4 | CCc1ccncc1 | 1.65 | -3.45 |
| 145 | 1-Chloro-4-nitrobenzene | 100-00-5 | Clc1ccc(cc1)N(=O)=O | 2.39 | -3.69 |
| 146 | Nitrobenzene | 98-95-3 | O=N(=O)c1ccccc1 | 1.85 | -3.00 |
| 147 | 2-Nitrotoluene | 88-72-2 | O=N(=O)c1ccccc1C | 2.30 | -3.28 |
| 148 | 2,6-Dinitrotoluene | 606-20-2 | Cc1c(cccc1N(=O)=O)N(=O)=O | 2.10 | -4.51 |
| 149 | 4-Chlorophenol | 106-48-9 | Oc1ccc(cc1)Cl | 2.39 | -4.58 |
| 150 | Ethyl benzoate | 93-89-0 | CCOC(=O)c1ccccc1 | 2.64 | -2.52 |
| 151 | Indole | 120-72-9 | c1ccc2c(c1)[nH]cc2 | 2.14 | -4.66 |
| 152 | 2,4-Dinitrotoluene | 121-14-2 | O=N(=O)c1ccc(c(c1)N(=O)=O)C | 1.98 | -5.65 |
| 153 | Hexachloroethane | 67-72-1 | ClC(C(Cl)(Cl)Cl)(Cl)Cl | 4.14 | -0.79 |
| 154 | Biphenyl | 92-52-4 | c1ccc(cc1)c1ccccc1 | 4.01 | -1.89 |
| 155 | isopropyl bromide | 75-26-3 | CC(Br)C | 2.14 | -0.34 |
| 156 | limonene | 138-86-3 | CC1=CCC(CC1)C(=C)C | 4.38 | 0.12 |
| 157 | Fluoromethane | 593-53-3 | CF | 0.51 | -0.15 |
| 158 | Methyl tert-butyl ether | 1634-04-4 | COC(C)(C)C | 0.94 | -1.61 |
| 159 | dimethyl sulfoxide | 67-68-5 | CS(=O)C | -1.35 | -7.20 |
| 160 | formic acid | 64-18-6 | OC=O | -0.54 | -5.16 |
| 161 | 4-Chloroaniline | 106-47-8 | Nc1ccc(cc1)Cl | 1.83 | -4.32 |
| 162 | Styrene | 100-42-5 | C=Cc1ccccc1 | 2.95 | -0.94 |
| 163 | Octane | 111-65-9 | CCCCCCCC | 5.18 | 2.13 |
| 164 | 3-Chlorophenol | 108-43-0 | Oc1cccc(c1)Cl | 2.50 | -4.84 |
| 165 | Benzyl acetate | 140-11-4 | CC(=O)OCc1ccccc1 | 1.96 | -3.33 |
| 166 | 1-Naphthol | 90-15-3 | Oc1cccc2c1cccc2 | 2.85 | -5.60 |
| 167 | 4-bromophenol | 106-41-2 | Oc1ccc(cc1)Br | 2.59 | -5.20 |
| 168 | N,N-Diethylaniline | 91-66-7 | CCN(c1ccccc1)CC | 3.31 | -2.10 |
| 169 | 4-n-Propylphenol | 645-56-7 | CCCc1ccc(cc1)O | 3.20 | -4.32 |
| 170 | 1,3-Dinitrobenzene | 99-65-0 | O=N(=O)c1cccc(c1)N(=O)=O | 1.49 | -5.69 |
| 171 | Anthracene | 120-12-7 | c1ccc2c(c1)cc1c(c2)cccc1 | 4.45 | -2.64 |
| 172 | Phenanthrene | 85-01-8 | c1ccc2c(c1)c1ccccc1cc2 | 4.46 | -2.75 |
| 173 | Fluoranthene | 206-44-0 | c1ccc2c(c1)c1cccc3c1c2ccc3 | 5.16 | -3.43 |
| 174 | Pyrene | 129-00-0 | c1cc2ccc3c4c2c(c1)ccc4ccc3 | 4.88 | -3.30 |
| 175 | Fluorene | 86-73-7 | c1ccc2c(c1)Cc1c2cccc1 | 4.18 | -2.40 |
| 176 | Acenaphthene | 83-32-9 | c1cc2cccc3c2c(c1)CC3 | 3.92 | -2.12 |
| 177 | phenol | 108-95-2 | Oc1ccccc1 | 1.46 | -4.86 |
| 178 | carbon disulfide | 75-15-0 | S=C=S | 1.94 | -0.22 |
| 179 | acetylene | 74-86-2 | C#C | 0.37 | -0.04 |
| 180 | methanal | 50-00-0 | C=O | 0.35 | -4.85 |
| 181 | 1-Propanethiol | 107-03-9 | CCCS | 1.81 | -0.77 |
| 182 | 1-Butanethiol | 109-79-5 | CCCCS | 2.28 | -0.72 |
| 183 | 2-Pentanol | 6032-29-7 | CCCC(O)C | 1.19 | -3.21 |
| 184 | 2-Hexanol | 626-93-7 | CCCCC(O)C | 1.76 | -2.99 |
| 185 | 3-Hexanol | 623-37-0 | CCCC(CC)O | 1.65 | -2.78 |
| 186 | 2-Heptanol | 543-49-7 | CCCCCC(O)C | 2.31 | -2.64 |
| 187 | 2,2,2-Trifluoroethanol | 75-89-8 | OCC(F)(F)F | 0.41 | -3.14 |
| 188 | 1,1,1-Trifluoro-2-propanol | 374-01-6 | CC(C(F)(F)F)O | 0.71 | -3.04 |
| 189 | 2,2,3,3,3-Pentafluoro-1-propanol | 422-05-9 | OCC(C(F)(F)F)(F)F | 1.23 | -3.03 |
| 190 | 1,1,1,3,3,3-Hexafluoro-2-propanol | 920-66-1 | OC(C(F)(F)F)C(F)(F)F | 1.66 | -2.75 |
| 191 | 2-Butanol | 78-92-2 | CCC(O)C | 0.61 | -3.42 |
| 192 | Triethyl phosphate | 78-40-0 | CCOP(=O)(OCC)OCC | 0.80 | -5.82 |
| 193 | 1,2-Dihydroxybenzene / Catechol | 120-80-9 | Oc1ccccc1O | 0.88 | -7.30 |
| 194 | PCB 28 | 7012-37-5 | Clc1ccc(cc1)c1ccc(cc1Cl)Cl | 5.62 | -2.08 |
| 195 | PCB 31 | 16606-02-3 | Clc1ccc(cc1)c1cc(Cl)ccc1Cl | 5.69 | -2.10 |
| 196 | PCB 44 | 41464-39-5 | Clc1ccc(c(c1)c1cccc(c1Cl)Cl)Cl | 5.81 | -2.23 |
| 197 | PCB 49 | 41464-40-8 | Clc1ccc(c(c1)Cl)c1cc(Cl)ccc1Cl | 6.22 | -2.06 |
| 198 | PCB 52 | 35693-99-3 | Clc1ccc(cc1c1cc(Cl)ccc1Cl)Cl | 6.09 | -2.08 |
| 199 | PCB 99 | 38380-01-7 | Clc1ccc(c(c1)Cl)c1cc(Cl)c(cc1Cl)Cl | 7.21 | -2.49 |
| 200 | PCB 101 | 37680-73-2 | Clc1ccc(c(c1)c1cc(Cl)c(cc1Cl)Cl)Cl | 6.80 | -2.43 |
| 201 | PCB 105 | 32598-14-4 | Clc1ccc(cc1Cl)c1ccc(c(c1Cl)Cl)Cl | 6.79 | -1.93 |
| 202 | PCB 118 | 31508-00-6 | Clc1cc(Cl)c(cc1c1ccc(c(c1)Cl)Cl)Cl | 7.12 | -1.92 |
| 203 | PCB 128 | 38380-07-3 | Clc1c(ccc(c1Cl)Cl)c1ccc(c(c1Cl)Cl)Cl | 7.31 | -3.27 |
| 204 | PCB 138 | 35065-28-2 | Clc1cc(Cl)c(cc1c1ccc(c(c1Cl)Cl)Cl)Cl | 7.44 | -3.06 |
| 205 | PCB 149 | 38380-04-0 | Clc1cc(Cl)c(cc1c1c(Cl)ccc(c1Cl)Cl)Cl | 7.28 | -1.24 |
| 206 | PCB 153 | 35065-27-1 | Clc1cc(Cl)c(cc1c1cc(Cl)c(cc1Cl)Cl)Cl | 7.75 | -3.02 |
| 207 | PCB 156 | 38380-08-4 | Clc1ccc(cc1Cl)c1cc(Cl)c(c(c1Cl)Cl)Cl | 7.60 | -2.22 |
| 208 | PCB 194 | 35694-08-7 | Clc1cc(c(c(c1Cl)Cl)Cl)c1cc(Cl)c(c(c1Cl)Cl)Cl | 8.68 | -3.38 |
| 209 | PCB 209 | 2051-24-3 | Clc1c(c2c(Cl)c(Cl)c(c(c2Cl)Cl)Cl)c(Cl)c(c(c1Cl)Cl)Cl | 8.27 | -0.41 |
| 210 | Naphthalene | 91-20-3 | c1ccc2c(c1)cccc2 | 3.30 | -1.74 |
| 211 | Benz[a]anthracene | 56-55-3 | c1ccc2c(c1)cc1c(c2)ccc2c1cccc2 | 5.76 | -3.30 |
| 212 | Chrysene | 218-01-9 | c1ccc2c(c1)c1ccc3c(c1cc2)cccc3 | 5.81 | -3.66 |
| 213 | Benzo[a]pyrene | 50-32-8 | c1ccc2c(c1)c1ccc3c4c1c(c2)ccc4ccc3 | 6.13 | -4.72 |
| 214 | Sulfur hexafluoride | 2551-62-4 | FS(F)(F)(F)(F)F | 1.68 | 2.27 |
| 215 | 1-Nitronaphthalene | 86-57-7 | O=N(=O)c1cccc2c1cccc2 | 3.19 | -4.13 |

## **Table S4**. Showing chemicals having experimental values of logK_ow_ and logK_aw_ from the logK_pw_ dataset.

| **S. No** | **Chemicals** | **CAS-RN** | **SMILES** | **K_ow__experimental** | **K_aw__experimental** |
| --- | --- | --- | --- | --- | --- |
| 1 | 1,1,1-Trichloroethane | 71-55-6 | CC(Cl)(Cl)Cl | 2.49 | -0.14 |
| 2 | 1,1,2,2-Tetrachloroethane | 79-34-5 | ClC(C(Cl)Cl)Cl | 2.39 | -1.82 |
| 3 | 1,1,2-Trichloroethane | 79-00-5 | ClCC(Cl)Cl | 1.89 | -1.46 |
| 4 | 1,1-Dichloroethane | 75-34-3 | CC(Cl)Cl | 1.79 | -0.63 |
| 5 | 1,1-Dichloroethene | 75-35-4 | ClC(=C)Cl | 2.13 | 0.04 |
| 6 | 1,2,3-Trimethylbenzene | 526-73-8 | Cc1c(C)cccc1C | 3.66 | -0.74 |
| 7 | 1,2,4-Trimethylbenzene | 95-63-6 | Cc1ccc(c(c1)C)C | 3.63 | -0.59 |
| 8 | 1,2-Dibromoethane | 106-93-4 | BrCCBr | 1.96 | -1.57 |
| 9 | 1,2-Dichlorobenzene | 95-50-1 | Clc1ccccc1Cl | 3.43 | -1.10 |
| 10 | 1,2-Dichloroethane | 107-06-2 | ClCCCl | 1.48 | -1.31 |
| 11 | 1,2-Dichloropropane | 78-87-5 | ClCC(Cl)C | 1.98 | -0.93 |
| 12 | 1,3,5-Trimethylbenzene | 108-67-8 | Cc1cc(C)cc(c1)C | 3.42 | -0.44 |
| 13 | 1,3-Dichlorobenzene | 541-73-1 | Clc1cccc(c1)Cl | 3.53 | -0.96 |
| 14 | 1-Butanol | 71-36-3 | CCCCO | 0.88 | -3.44 |
| 15 | 1-Chlorobutane | 109-69-3 | CCCCCl | 2.64 | -0.16 |
| 16 | 1-Chloropentane | 543-59-9 | CCCCCCl | 2.73 | 0.00 |
| 17 | 1-Chloropropane | 540-54-5 | CCCCl | 2.04 | -0.26 |
| 18 | 1-Hexanol | 111-27-3 | CCCCCCO | 2.03 | -3.15 |
| 19 | 1-Nitropropane | 108-03-2 | CCCN(=O)=O | 0.87 | -2.44 |
| 20 | 1-Pentanol | 71-41-0 | CCCCCO | 1.51 | -3.27 |
| 21 | 1-Propanol | 71-23-8 | CCCO | 0.25 | -3.51 |
| 22 | 2,2-Dimethylbutane | 75-83-2 | CCC(C)(C)C | 3.82 | 1.80 |
| 23 | 2-Butoxyethanol | 111-76-2 | CCCCOCCO | 0.83 | -4.18 |
| 24 | 2-Chloropropane | 75-29-6 | CC(Cl)C | 1.90 | -0.14 |
| 25 | 2-Ethoxyethanol | 110-80-5 | OCCOCC | -0.32 | -4.71 |
| 26 | 2-Heptanone | 110-43-0 | CCCCCC(=O)C | 1.98 | -2.15 |
| 27 | 2-Hexanone | 591-78-6 | CCCCC(=O)C | 1.38 | -2.41 |
| 28 | 2-Isopropoxyethanol | 109-59-1 | OCCOC(C)C | 0.05 | -4.42 |
| 29 | 2-Methoxyethanol | 109-86-4 | COCCO | -0.77 | -4.86 |
| 30 | 2-Methyl-1-propanol | 78-83-1 | OCC(C)C | 0.76 | -3.39 |
| 31 | 2-Methyl-2-propanol | 75-65-0 | CC(O)(C)C | 0.35 | -3.42 |
| 32 | 2-Nitropropane | 79-46-9 | CC(N(=O)=O)C | 0.93 | -2.30 |
| 33 | 2-Pentanone | 107-87-9 | CCCC(=O)C | 0.91 | -2.46 |
| 34 | 2-Propanol | 67-63-0 | CC(O)C | 0.05 | -3.47 |
| 35 | 3-Methyl-1-butanol | 137-32-6 | CCC(CO)C | 1.29 | -3.23 |
| 36 | 3-Methylpentane | 96-14-0 | CCC(CC)C | 3.60 | 1.85 |
| 37 | 3-Pentanone | 96-22-0 | CCC(=O)CC | 0.99 | -2.68 |
| 38 | 4-Methyl-2-pentanone | 108-10-1 | CC(CC(=O)C)C | 1.31 | -2.24 |
| 39 | Acetone | 67-64-1 | CC(=O)C | -0.24 | -2.84 |
| 40 | Benzene | 71-43-2 | c1ccccc1 | 2.13 | -0.64 |
| 41 | Bromochloromethane | 74-97-5 | ClCBr | 1.41 | -1.22 |
| 42 | 2-Butanone | 78-93-3 | CCC(=O)C | 0.29 | -2.63 |
| 43 | Butane | 106-97-8 | CCCC | 2.89 | 1.60 |
| 44 | Butyl acetate | 123-86-4 | CCCCOC(=O)C | 1.78 | -1.93 |
| 45 | Carbon tetrachloride | 56-23-5 | ClC(Cl)(Cl)Cl | 2.83 | 0.06 |
| 46 | 1,2-Dichlorotetrafluoroethane | 76-14-2 | FC(C(Cl)(F)F)(Cl)F | 2.82 | 2.07 |
| 47 | 1,1-Difluoroethane | 75-37-6 | CC(F)F | 0.75 | -0.07 |
| 48 | Halothane | 151-67-7 | ClC(C(F)(F)F)Br | 2.30 | -0.07 |
| 49 | Carbon tetrafluoride | 75-73-0 | C(F)(F)(F)F | 1.18 | 2.33 |
| 50 | Isoflurane | 26675-46-7 | FC(OC(C(F)(F)F)Cl)F | 2.06 | 0.08 |
| 51 | Chlorobenzene | 108-90-7 | Clc1ccccc1 | 2.84 | -0.89 |
| 52 | Chlorodibromomethane | 124-48-1 | ClC(Br)Br | 2.16 | -1.49 |
| 53 | Chloroethane | 75-00-3 | CCCl | 1.43 | -0.33 |
| 54 | Chloroform | 67-66-3 | ClC(Cl)Cl | 1.97 | -0.82 |
| 55 | cis-1,2-Dichloroethene | 156-59-2 | Cl/C=C\Cl | 2.00 | -0.77 |
| 56 | Cycloheptane | 291-64-5 | C1CCCCCC1 | 4.00 | 0.59 |
| 57 | Cyclohexane | 110-82-7 | C1CCCCC1 | 3.44 | 0.80 |
| 58 | Cyclopentane | 287-92-3 | C1CCCC1 | 3.00 | 0.80 |
| 59 | Cyclopropane | 75-19-4 | C1CC1 | 1.72 | 1.52 |
| 60 | Decane | 124-18-5 | CCCCCCCCCC | 5.01 | 2.33 |
| 61 | Dibromethane | 74-95-3 | BrCBr | 1.70 | -1.47 |
| 62 | Dichloromethane | 75-09-2 | ClCCl | 1.25 | -0.87 |
| 63 | Diethyl ether | 60-29-7 | CCOCC | 0.89 | -1.29 |
| 64 | Ethane | 74-84-0 | CC | 1.81 | 1.32 |
| 65 | Ethanol | 64-17-5 | CCO | -0.31 | -3.68 |
| 66 | Ethene | 74-85-1 | C=C | 1.13 | 0.98 |
| 67 | Ethyl acetate | 141-78-6 | CCOC(=O)C | 0.73 | -2.25 |
| 68 | Ethylbenzene | 100-41-4 | CCc1ccccc1 | 3.15 | -0.48 |
| 69 | Fluorobenzene | 462-06-6 | Fc1ccccc1 | 2.27 | -0.58 |
| 70 | Fluorochloromethane | 593-70-4 | FCCl | 0.51 | -0.56 |
| 71 | Heptane | 142-82-5 | CCCCCCC | 4.66 | 1.92 |
| 72 | Hexane | 110-54-3 | CCCCCC | 3.90 | 1.88 |
| 73 | Isobutyl acetate | 110-19-0 | CC(COC(=O)C)C | 1.78 | -1.72 |
| 74 | Isopentyl acetate | 123-92-2 | CC(CCOC(=O)C)C | 2.25 | -1.61 |
| 75 | Isopropylbenzene | 98-82-8 | CC(c1ccccc1)C | 3.66 | -0.32 |
| 76 | Methoxyflurane | 76-38-0 | COC(C(Cl)Cl)(F)F | 2.21 | -0.81 |
| 77 | Methane | 74-82-8 | C | 1.09 | 1.44 |
| 78 | Methanol | 67-56-1 | CO | -0.77 | -3.72 |
| 79 | Methyl acetate | 79-20-9 | COC(=O)C | 0.18 | -2.32 |
| 80 | Methyl chloride | 74-87-3 | CCl | 0.91 | -0.43 |
| 81 | Methylcyclopentane | 96-37-7 | CC1CCCC1 | 3.37 | 1.18 |
| 82 | m-Xylene | 108-38-3 | Cc1cccc(c1)C | 3.20 | -0.52 |
| 83 | Nonane | 111-84-2 | CCCCCCCCC | 5.65 | 2.15 |
| 84 | o-Xylene | 95-47-6 | Cc1ccccc1C | 3.16 | -0.67 |
| 85 | p-Xylene | 106-42-3 | Cc1ccc(cc1)C | 3.15 | -0.54 |
| 86 | Pentachloroethane | 76-01-7 | ClC(C(Cl)(Cl)Cl)Cl | 3.22 | -1.09 |
| 87 | Pentane | 109-66-0 | CCCCC | 3.39 | 1.72 |
| 88 | Pentyl acetate | 628-63-7 | CCCCCOC(=O)C | 2.30 | -1.79 |
| 89 | Propane | 74-98-6 | CCC | 2.36 | 1.47 |
| 90 | Propyl acetate | 109-60-4 | CCCOC(=O)C | 1.24 | -2.04 |
| 91 | Propylbenzene | 103-65-1 | CCCc1ccccc1 | 3.69 | -0.36 |
| 92 | Tetrachloroethene | 127-18-4 | ClC(=C(Cl)Cl)Cl | 3.40 | -0.13 |
| 93 | Toluene | 108-88-3 | Cc1ccccc1 | 2.73 | -0.56 |

## **Table S5**. Showing chemicals with their experimental and estimated values (bold format) of logK_ow_ and logK_aw_ from EPI Suite used to formulate tp-LFER model equation of logK_lw_.

| **S. No** | **Chemicals** | **CAS-RN** | **SMILES** | **logK_ow_** | **logK_aw_** |
| --- | --- | --- | --- | --- | --- |
| 1 | 1,1,1,2-Tetrachloroethane | 630-20-6 | ClCC(Cl)(Cl)Cl | **2.84** | -0.98 |
| 2 | 1,1,1-Trichloroethane | 71-55-6 | CC(Cl)(Cl)Cl | 2.49 | -0.14 |
| 3 | 1,1,2,2-Tetrachloroethane | 79-34-5 | ClC(C(Cl)Cl)Cl | 2.39 | -1.82 |
| 4 | 1,1,2-Trichloroethane | 79-00-5 | ClCC(Cl)Cl | 1.89 | -1.46 |
| 5 | 1,1-Dichloro-1-fluoroethane | 1717-00-6 | CC(Cl)(Cl)F | **1.96** | -0.04 |
| 6 | 1,1-Dichloroethane | 75-34-3 | CC(Cl)Cl | 1.79 | -0.63 |
| 7 | 1,1-Dichloroethene | 75-35-4 | ClC(=C)Cl | 2.13 | 0.04 |
| 8 | 1,2,3-Trimethylbenzene | 526-73-8 | Cc1c(C)cccc1C | 3.66 | -0.74 |
| 9 | 1,2,4-Trifluorobenzene | 367-23-7 | Fc1ccc(c(c1)F)F | 2.52 | **-0.38** |
| 10 | 1,2,4-Trimethylbenzene | 95-63-6 | Cc1ccc(c(c1)C)C | 3.63 | -0.59 |
| 11 | 1,2-Dibromoethane | 106-93-4 | BrCCBr | 1.96 | -1.57 |
| 12 | 1,2-Dichlorobenzene | 95-50-1 | Clc1ccccc1Cl | 3.43 | -1.10 |
| 13 | 1,2-Dichloroethane | 107-06-2 | ClCCCl | 1.48 | -1.31 |
| 14 | 1,2-Dichloropropane | 78-87-5 | ClCC(Cl)C | 1.98 | -0.93 |
| 15 | 1,2-Difluorobenzene | 367-11-3 | Fc1ccccc1F | 2.37 | **-0.45** |
| 16 | 1,3,5-Trifluorobenzene | 372-38-3 | Fc1cc(F)cc(c1)F | **2.53** | **0.13** |
| 17 | 1,3,5-Trimethylbenzene | 108-67-8 | Cc1cc(C)cc(c1)C | 3.42 | -0.44 |
| 18 | 1,3-Dichlorobenzene | 541-73-1 | Clc1cccc(c1)Cl | 3.53 | -0.96 |
| 19 | 1,4-Difluorobenzene | 540-36-3 | Fc1ccc(cc1)F | 2.13 | **-0.35** |
| 20 | 1-Bromo-2-chloroethane | 107-04-0 | ClCCBr | **1.96** | -1.42 |
| 21 | 1-Butanol | 71-36-3 | CCCCO | 0.88 | -3.44 |
| 22 | 1-Chlorobutane | 109-69-3 | CCCCCl | 2.64 | -0.16 |
| 23 | 1-Chloropentane | 543-59-9 | CCCCCCl | 2.73 | 0.00 |
| 24 | 1-Chloropropane | 540-54-5 | CCCCl | 2.04 | -0.26 |
| 25 | 1-Hexanol | 111-27-3 | CCCCCCO | 2.03 | -3.15 |
| 26 | 1-Methoxy-2-propanol | 107-98-2 | COCC(O)C | **-0.26** | -4.42 |
| 27 | 1-Nitropropane | 108-03-2 | CCCN(=O)=O | 0.87 | -2.44 |
| 28 | 1-Pentanol | 71-41-0 | CCCCCO | 1.51 | -3.27 |
| 29 | 1-Propanol | 71-23-8 | CCCO | 0.25 | -3.51 |
| 30 | 2,2,4-Trimethylpentane | 540-84-1 | CC(CC(C)(C)C)C | **4.65** | 2.10 |
| 31 | 2,2-Dichloro-1,1,1-trifluoroethane | 306-83-2 | ClC(C(F)(F)F)Cl | **2.15** | 0.03 |
| 32 | 2,2-Dimethylbutane | 75-83-2 | CCC(C)(C)C | 3.82 | 1.80 |
| 33 | 2,3,4-Trimethylpentane | 565-75-3 | CC(C(C)C)C(C)C | **4.82** | 1.87 |
| 34 | 2-Butoxyethanol | 111-76-2 | CCCCOCCO | 0.83 | -4.18 |
| 35 | 2-Chloropropane | 75-29-6 | CC(Cl)C | 1.9 | -0.14 |
| 36 | 2-Ethoxyethanol | 110-80-5 | OCCOCC | -0.32 | -4.71 |
| 37 | 2-Fluoropropane | 420-26-8 | CC(F)C | **1.33** | **0.33** |
| 38 | 2-Heptanone | 110-43-0 | CCCCCC(=O)C | 1.98 | -2.15 |
| 39 | 2-Hexanone | 591-78-6 | CCCCC(=O)C | 1.38 | -2.41 |
| 40 | 2-Isopropoxyethanol | 109-59-1 | OCCOC(C)C | 0.05 | -4.42 |
| 41 | 2-Methoxyethanol | 109-86-4 | COCCO | -0.77 | -4.86 |
| 42 | 2-Methyl-1-propanol | 78-83-1 | OCC(C)C | 0.76 | -3.39 |
| 43 | 2-Methyl-2-propanol | 75-65-0 | CC(O)(C)C | 0.35 | -3.42 |
| 44 | 2-Methylpentane | 107-83-5 | CCCC(C)C | **3.72** | 1.85 |
| 45 | 2-Nitropropane | 79-46-9 | CC(N(=O)=O)C | 0.93 | -2.30 |
| 46 | 2-Pentanone | 107-87-9 | CCCC(=O)C | 0.91 | -2.46 |
| 47 | 2-Propanol | 67-63-0 | CC(O)C | 0.05 | -3.47 |
| 48 | 3-Methyl-1-butanol | 137-32-6 | CCC(CO)C | 1.29 | -3.23 |
| 49 | 3-Methylhexane | 589-34-4 | CCCC(CC)C | **4.29** | 1.83 |
| 50 | 3-Methylpentane | 96-14-0 | CCC(CC)C | 3.6 | 1.85 |
| 51 | 3-Pentanone | 96-22-0 | CCC(=O)CC | 0.99 | -2.68 |
| 52 | 4-Methyl-2-pentanone | 108-10-1 | CC(CC(=O)C)C | 1.31 | -2.24 |
| 53 | Acetone | 67-64-1 | CC(=O)C | -0.24 | -2.84 |
| 54 | Allylbenzene | 300-57-2 | C=CCc1ccccc1 | 3.23 | **-0.91** |
| 55 | Benzene | 71-43-2 | c1ccccc1 | 2.13 | -0.64 |
| 56 | Bromochloromethane | 74-97-5 | ClCBr | 1.41 | -1.22 |
| 57 | 2-Butanone | 78-93-3 | CCC(=O)C | 0.29 | -2.63 |
| 58 | Butane | 106-97-8 | CCCC | 2.89 | 1.60 |
| 59 | Butyl acetate | 123-86-4 | CCCCOC(=O)C | 1.78 | -1.93 |
| 60 | Carbon tetrachloride | 56-23-5 | ClC(Cl)(Cl)Cl | 2.83 | 0.06 |
| 61 | 1,2-Dichlorotetrafluoroethane | 76-14-2 | FC(C(Cl)(F)F)(Cl)F | 2.82 | 2.07 |
| 62 | 1,1,2,2,3,3,4,4-Octafluorobutane | 377-36-6 | FC(C(C(C(F)F)(F)F)(F)F)F | **2.27** | **0.52** |
| 63 | 1,1,2,2,3,3-Hexafluoropropane | 680-00-2 | FC(C(C(F)F)(F)F)F | **1.48** | **0.45** |
| 64 | 1,1,2,2-Tetrafluoroethane | 359-35-3 | FC(C(F)F)F | **0.85** | **0.13** |
| 65 | 1,1-Difluoroethane | 75-37-6 | CC(F)F | 0.75 | -0.07 |
| 66 | Halothane | 151-67-7 | ClC(C(F)(F)F)Br | 2.3 | -0.07 |
| 67 | 1,1,1,2-Tetrafluoroethane | 811-97-2 | FCC(F)(F)F | **1.20** | 0.32 |
| 68 | Fluroxene | 406-90-6 | C=COCC(F)(F)F | **1.46** | **-0.22** |
| 69 | Carbon tetrafluoride | 75-73-0 | C(F)(F)(F)F | 1.18 | 2.33 |
| 70 | 1,3-Difluoropropane | 462-39-5 | FCCCF | **0.76** | **-1.21** |
| 71 | Enflurane | 13838-16-9 | FC(OC(C(Cl)F)(F)F)F | 2.1 | **0.03** |
| 72 | Isoflurane | 26675-46-7 | FC(OC(C(F)(F)F)Cl)F | 2.06 | 0.08 |
| 73 | Desflurane | 57041-67-5 | FC(OC(C(F)(F)F)F)F | **1.76** | **0.73** |
| 74 | Sevoflurane | 28523-86-6 | FCOC(C(F)(F)F)C(F)(F)F | **1.91** | **0.40** |
| 75 | Chlorobenzene | 108-90-7 | Clc1ccccc1 | 2.84 | -0.89 |
| 76 | Chlorodibromomethane | 124-48-1 | ClC(Br)Br | 2.16 | -1.49 |
| 77 | Chloroethane | 75-00-3 | CCCl | 1.43 | -0.33 |
| 78 | Chloroform | 67-66-3 | ClC(Cl)Cl | 1.97 | -0.82 |
| 79 | cis-1,2-Dichloroethene | 156-59-2 | Cl/C=C\Cl | 2 | -0.77 |
| 80 | Cycloheptane | 291-64-5 | C1CCCCCC1 | 4 | 0.59 |
| 81 | Cyclohexane | 110-82-7 | C1CCCCC1 | 3.44 | 0.80 |
| 82 | Cyclopentane | 287-92-3 | C1CCCC1 | 3 | 0.80 |
| 83 | Cyclopropane | 75-19-4 | C1CC1 | 1.72 | 1.52 |
| 84 | Decane | 124-18-5 | CCCCCCCCCC | 5.01 | 2.33 |
| 85 | Dibromethane | 74-95-3 | BrCBr | 1.7 | -1.47 |
| 86 | Dichloromethane | 75-09-2 | ClCCl | 1.25 | -0.87 |
| 87 | Diethyl ether | 60-29-7 | CCOCC | 0.89 | -1.29 |
| 88 | Difluoromethane | 75-10-5 | FCF | 0.2 | **-0.18** |
| 89 | Divinyl ether | 109-93-3 | C=COC=C | **1.65** | -0.47 |
| 90 | Ethane | 74-84-0 | CC | 1.81 | 1.32 |
| 91 | Ethanol | 64-17-5 | CCO | -0.31 | -3.68 |
| 92 | Ethene | 74-85-1 | C=C | 1.13 | 0.98 |
| 93 | Ethyl acetate | 141-78-6 | CCOC(=O)C | 0.73 | -2.25 |
| 94 | Ethyl tert-butyl ether | 637-92-3 | CCOC(C)(C)C | **1.72** | -1.17 |
| 95 | Ethyl tert-pentyl ether | 919-94-8 | CCOC(CC)(C)C | **2.19** | -0.10 |
| 96 | Ethylbenzene | 100-41-4 | CCc1ccccc1 | 3.15 | -0.48 |
| 97 | Fluorobenzene | 462-06-6 | Fc1ccccc1 | 2.27 | -0.58 |
| 98 | Fluoroethane | 353-36-6 | CCF | **0.73** | -0.03 |
| 99 | Fluorochloromethane | 593-70-4 | FCCl | 0.51 | -0.56 |
| 100 | Heptane | 142-82-5 | CCCCCCC | 4.66 | 1.92 |
| 101 | Hexafluoro benzene | 392-56-3 | Fc1c(F)c(F)c(c(c1F)F)F | 2.55 | **0.35** |
| 102 | Hexane | 110-54-3 | CCCCCC | 3.9 | 1.88 |
| 103 | Isobutyl acetate | 110-19-0 | CC(COC(=O)C)C | 1.78 | -1.72 |
| 104 | Isopentyl acetate | 123-92-2 | CC(CCOC(=O)C)C | 2.25 | -1.61 |
| 105 | Isopropyl acetate | 108-21-4 | CC(OC(=O)C)C | **1.15** | -1.94 |
| 106 | Isopropylbenzene | 98-82-8 | CC(c1ccccc1)C | 3.66 | -0.32 |
| 107 | Methoxyflurane | 76-38-0 | COC(C(Cl)Cl)(F)F | 2.21 | -0.81 |
| 108 | Methane | 74-82-8 | C | 1.09 | 1.44 |
| 109 | Methanol | 67-56-1 | CO | -0.77 | -3.72 |
| 110 | Methyl acetate | 79-20-9 | COC(=O)C | 0.18 | -2.32 |
| 111 | Methyl chloride | 74-87-3 | CCl | 0.91 | -0.43 |
| 112 | Methylcyclopentane | 96-37-7 | CC1CCCC1 | 3.37 | 1.18 |
| 113 | Methylpentafluorobenzene | 771-56-2 | Fc1c(C)c(F)c(c(c1F)F)F | **3.15** | **0.17** |
| 114 | m-Methylstyrene | 100-80-1 | C=Cc1cccc(c1)C | **3.33** | **-0.94** |
| 115 | m-Xylene | 108-38-3 | Cc1cccc(c1)C | 3.2 | -0.52 |
| 116 | Nonane | 111-84-2 | CCCCCCCCC | 5.65 | 2.15 |
| 117 | o-Xylene | 95-47-6 | Cc1ccccc1C | 3.16 | -0.67 |
| 118 | p-Xylene | 106-42-3 | Cc1ccc(cc1)C | 3.15 | -0.54 |
| 119 | Pentachloroethane | 76-01-7 | ClC(C(Cl)(Cl)Cl)Cl | 3.22 | -1.09 |
| 120 | Pentafluorobenzene | 363-72-4 | Fc1cc(F)c(c(c1F)F)F | 2.53 | **-0.10** |
| 121 | Pentane | 109-66-0 | CCCCC | 3.39 | 1.72 |
| 122 | Pentyl acetate | 628-63-7 | CCCCCOC(=O)C | 2.3 | -1.79 |
| 123 | p-Methylstyrene | 622-97-9 | C=Cc1ccc(cc1)C | **3.34** | -0.88 |
| 124 | Propane | 74-98-6 | CCC | 2.36 | 1.47 |
| 125 | Propyl acetate | 109-60-4 | CCCOC(=O)C | 1.24 | -2.04 |
| 126 | Propylbenzene | 103-65-1 | CCCc1ccccc1 | 3.69 | -0.36 |
| 127 | Tetrachloroethene | 127-18-4 | ClC(=C(Cl)Cl)Cl | 3.4 | -0.13 |
| 128 | Toluene | 108-88-3 | Cc1ccccc1 | 2.73 | -0.56 |
| 129 | trans-1,2-Dichloroethene | 156-60-5 | Cl/C=C/Cl | 2 | -0.77 |
| 130 | Trichloroethene | 79-01-6 | ClC=C(Cl)Cl | 2.42 | -0.39 |
| 131 | tridecane | 629-50-5 | CCCCCCCCCCCCC | **7.68** | 2.08 |
| 132 | triethylamine | 121-44-8 | CCN(CC)CC | 1.45 | -2.21 |
| 133 | undecane | 1120-21-4 | CCCCCCCCCCC | **6.57** | 1.91 |
| 134 | alpha-pinene | 80-56-8 | CC1=CCC2CC1C2(C)C | 4.44 | 1.09 |
| 135 | 1,2-dimethoxyethane | 110-71-4 | COCCOC | -0.21 | **-3.37** |
| 136 | 1,4-dioxane | 123-91-1 | O1CCOCC1 | -0.27 | -3.70 |
| 137 | 1-heptanol | 111-70-6 | CCCCCCCO | 2.62 | -3.11 |
| 138 | 2-methylpyridine | 109-06-8 | Cc1ccccn1 | 1.11 | -3.38 |
| 139 | 3-methylpyridine | 108-99-6 | Cc1cccnc1 | 1.2 | -3.49 |
| 140 | 4-methylpyridine | 108-89-4 | Cc1ccncc1 | 1.22 | -3.60 |
| 141 | benzyl alcohol | 100-51-6 | OCc1ccccc1 | 1.1 | -4.85 |
| 142 | bromobenzene | 108-86-1 | Brc1ccccc1 | 2.99 | -0.99 |
| 143 | butyl formate | 592-84-7 | CCCCOC=O | **1.55** | -1.67 |
| 144 | butyl propanoate | 590-01-2 | CCCCOC(=O)CC | **2.40** | -1.68 |
| 145 | butylbenzene | 104-51-8 | CCCCc1ccccc1 | 4.38 | -0.18 |
| 146 | 1,1-difluoro-2-chloroethene | 359-10-4 | ClC=C(F)F | **1.45** | **0.40** |
| 147 | 1-chloro-2,2,2-trifluoroethane | 75-88-7 | ClCC(F)(F)F | **1.62** | **0.09** |
| 148 | bis-(2,2,2-trifluoroethyl)ether | 333-36-8 | FC(COCC(F)(F)F)(F)F | **1.69** | **-0.09** |
| 149 | cyclohexene | 110-83-8 | C1CCC=CC1 | 2.86 | 0.28 |
| 150 | cyclopentanone | 120-92-3 | O=C1CCCC1 | 0.38 | -3.38 |
| 151 | difluorochloromethane | 75-45-6 | FC(Cl)F | 1.08 | 0.23 |
| 152 | diisopropyl ether | 108-20-3 | CC(OC(C)C)C | 1.52 | -0.97 |
| 153 | dimethoxymethane | 109-87-5 | COCOC | 0 | -2.14 |
| 154 | dimethyl ether | 115-10-6 | COC | 0.1 | -1.38 |
| 155 | dimethylacetamide | 127-19-5 | CC(=O)N(C)C | -0.77 | -6.26 |
| 156 | dimethylformamide | 68-12-2 | O=CN(C)C | -1.01 | -5.51 |
| 157 | di-n-butyl ether | 142-96-1 | CCCCOCCCC | 3.21 | -0.60 |
| 158 | dodecane | 112-40-3 | CCCCCCCCCCCC | 6.1 | 2.53 |
| 159 | ethyl formate | 109-94-4 | CCOC=O | 0.23 | -1.79 |
| 160 | ethyl propanoate | 105-37-3 | CCOC(=O)CC | 1.21 | -1.98 |
| 161 | iodoethane | 75-03-6 | CCI | 2 | -0.54 |
| 162 | fluorotrichloromethane | 75-69-4 | FC(Cl)(Cl)Cl | 2.53 | 0.61 |
| 163 | methyl formate | 107-31-3 | COC=O | 0.03 | -2.03 |
| 164 | methylcyclohexane | 108-87-2 | CC1CCCCC1 | 3.61 | 1.25 |
| 165 | nitroethane | 79-24-3 | CCN(=O)=O | 0.18 | -2.70 |
| 166 | nitromethane | 75-52-5 | CN(=O)=O | -0.35 | -2.92 |
| 167 | N, N-dimethylaniline | 121-69-7 | CN(c1ccccc1)C | 2.31 | -2.63 |
| 168 | pentadecane | 629-62-9 | CCCCCCCCCCCCCCC | **8.80** | 2.72 |
| 169 | piperidine | 110-89-4 | C1CCCNC1 | 0.84 | -3.73 |
| 170 | propyl bromide | 106-94-5 | CCCBr | 2.1 | -0.52 |
| 171 | propyl formate | 110-74-7 | CCCOC=O | 0.83 | -1.74 |
| 172 | pyridine | 110-86-1 | c1cccnc1 | 0.65 | -3.34 |
| 173 | tetradecane | 629-59-4 | CCCCCCCCCCCCCC | 7.2 | 2.58 |
| 174 | tetrahydrofuran | 109-99-9 | C1CCCO1 | 0.46 | -2.53 |
| 175 | halo propane | 679-84-5 | BrCC(C(F)F)(F)F | **2.65** | **0.28** |
| 176 | Cyclooctane | 292-64-8 | C1CCCCCCC1 | 4.45 | **1.18** |
| 177 | Octan-1-ol | 111-87-5 | CCCCCCCCO | 3 | -2.99 |
| 178 | Nonan-1-ol | 143-08-8 | CCCCCCCCCO | 3.77 | -2.89 |
| 179 | Hexanal | 66-25-1 | CCCCCC=O | 1.78 | -2.05 |
| 180 | Heptanal | 111-71-7 | CCCCCCC=O | **2.21** | -1.95 |
| 181 | Octanal | 124-13-0 | CCCCCCCC=O | **2.76** | -1.67 |
| 182 | Nonanal / n-Nonyl Aldehyde | 124-19-6 | CCCCCCCCC=O | **3.31** | -1.51 |
| 183 | 1-Chloroheptane | 629-06-1 | CCCCCCCCl | 4.15 | **0.32** |
| 184 | 1-Chlorooctane | 111-85-3 | CCCCCCCCCl | **4.76** | 0.20 |
| 185 | 1-Hexene | 592-41-6 | CCCCC=C | 3.39 | 1.23 |
| 186 | 1-Heptene | 592-76-7 | CCCCCC=C | 3.99 | 1.24 |
| 187 | 1-Octene | 111-66-0 | CCCCCCC=C | 4.57 | 1.42 |
| 188 | 1-Nonene | 124-11-8 | CCCCCCCC=C | 5.15 | 1.52 |
| 189 | 1-Decene | 872-05-9 | CCCCCCCCC=C | 5.7 | 1.35 |
| 190 | 1,2,4-Trichlorobenzene | 120-82-1 | Clc1ccc(c(c1)Cl)Cl | 4.02 | -1.23 |
| 191 | Di-n-propyl ether | 111-43-3 | CCCOCCC | 2.03 | -1.04 |
| 192 | Dipentyl ether | 693-65-2 | CCCCCOCCCCC | **4.33** | **-0.44** |
| 193 | 2-octanone | 111-13-7 | CCCCCCC(=O)C | 2.37 | -2.11 |
| 194 | 2-nonanone | 821-55-6 | CCCCCCCC(=O)C | 3.14 | -1.82 |
| 195 | 1-Nitrobutane | 627-05-4 | CCCCN(=O)=O | 1.47 | -2.30 |
| 196 | 1-Nitrohexane | 646-14-0 | CCCCCCN(=O)=O | 2.7 | **-2.03** |
| 197 | 4-Ethylpyridine | 536-75-4 | CCc1ccncc1 | 1.65 | -3.45 |
| 198 | 1-Chloro-4-nitrobenzene | 100-00-5 | Clc1ccc(cc1)N(=O)=O | 2.39 | -3.69 |
| 199 | Nitrobenzene | 98-95-3 | O=N(=O)c1ccccc1 | 1.85 | -3.00 |
| 200 | 2-Nitrotoluene | 88-72-2 | O=N(=O)c1ccccc1C | 2.3 | -3.28 |
| 201 | 2,6-Dinitrotoluene | 606-20-2 | Cc1c(cccc1N(=O)=O)N(=O)=O | 2.1 | -4.51 |
| 202 | 4-Nitroanisole | 100-17-4 | COc1ccc(cc1)N(=O)=O | 2.03 | **-4.67** |
| 203 | 1,4-Dimethoxybenzene | 150-78-7 | COc1ccc(cc1)OC | 2.04 | **-3.49** |
| 204 | 4-Chlorophenol | 106-48-9 | Oc1ccc(cc1)Cl | 2.39 | -4.58 |
| 205 | Ethyl benzoate | 93-89-0 | CCOC(=O)c1ccccc1 | 2.64 | -2.52 |
| 206 | Indole | 120-72-9 | c1ccc2c(c1)[nH]cc2 | 2.14 | -4.66 |
| 207 | 2-Ethyl-1-hexanol | 104-76-7 | CCCCC(CO)CC | **3.09** | -2.96 |
| 208 | 3-Ethyl-3-hexanol | 597-76-2 | CCCC(CC)(CC)O | **2.76** | **-2.85** |
| 209 | 4-Ethyl-3-hexanol | 19780-44-0 | CCC(C(CC)O)CC | **2.72** | **-2.85** |
| 210 | 3-Ethyl-3-pentanol | 597-49-9 | CCC(CC)(CC)O | **2.08** | **-3.19** |
| 211 | 2,4-Dinitrotoluene | 121-14-2 | O=N(=O)c1ccc(c(c1)N(=O)=O)C | 1.98 | -5.65 |
| 212 | 1-fluropropane | 460-13-9 | CCCF | **1.20** | **0.10** |
| 213 | Hexachloroethane | 67-72-1 | ClC(C(Cl)(Cl)Cl)(Cl)Cl | 4.14 | -0.79 |
| 214 | Biphenyl | 92-52-4 | c1ccc(cc1)c1ccccc1 | 4.01 | -1.89 |
| 215 | hexadecane | 544-76-3 | CCCCCCCCCCCCCCCC | **9.35** | 1.29 |
| 216 | isopropyl bromide | 75-26-3 | CC(Br)C | 2.14 | -0.34 |
| 217 | beta-pinene | 127-91-3 | C=C1CCC2CC1C2(C)C | 4.16 | **0.27** |
| 218 | limonene | 138-86-3 | CC1=CCC(CC1)C(=C)C | 4.38 | 0.12 |
| 219 | Fluoromethane | 593-53-3 | CF | 0.51 | -0.15 |
| 220 | Tricyclo[5.2.1.0(2,6)]decane | 2825-83-4 | C1CC2C(C1)C1CC2CC1 | **4.45** | **0.08** |
| 221 | Methyl tert-butyl ether | 1634-04-4 | COC(C)(C)C | 0.94 | -1.61 |
| 222 | vinyl chloride | 75-01-4 | ClC=C | **1.37** | 0.06 |
| 223 | dimethyl sulfoxide | 67-68-5 | CS(=O)C | -1.35 | -7.20 |
| 224 | formic acid | 64-18-6 | OC=O | -0.54 | -5.16 |
| 225 | 3-carene | 13466-78-9 | CC1=CCC2C(C1)C2(C)C | 4.38 | **0.62** |
| 226 | vinyl bromide | 593-60-2 | BrC=C | 1.57 | **-0.34** |
| 227 | 4-Chloroaniline | 106-47-8 | Nc1ccc(cc1)Cl | 1.83 | -4.32 |
| 228 | allyl chloride | 107-05-1 | ClCC=C | **1.76** | -0.34 |
| 229 | Styrene | 100-42-5 | C=Cc1ccccc1 | 2.95 | -0.94 |
| 230 | Octane | 111-65-9 | CCCCCCCC | 5.18 | 2.13 |
| 231 | 3-Chlorophenol | 108-43-0 | Oc1cccc(c1)Cl | 2.5 | -4.84 |
| 232 | Benzyl acetate | 140-11-4 | CC(=O)OCc1ccccc1 | 1.96 | -3.33 |
| 233 | 1-Naphthol | 90-15-3 | Oc1cccc2c1cccc2 | 2.85 | -5.60 |
| 234 | 4-bromophenol | 106-41-2 | Oc1ccc(cc1)Br | 2.59 | -5.20 |
| 235 | 4-Iodoaniline | 540-37-4 | Nc1ccc(cc1)I | 2.34 | **-5.14** |
| 236 | N, N-Diethyl aniline | 91-66-7 | CCN(c1ccccc1)CC | 3.31 | -2.10 |
| 237 | 4-n-Propylphenol | 645-56-7 | CCCc1ccc(cc1)O | 3.2 | -4.32 |
| 238 | 4-iodophenol | 540-38-5 | Oc1ccc(cc1)I | 2.91 | **-5.41** |
| 239 | 1,3-Dinitrobenzene | 99-65-0 | O=N(=O)c1cccc(c1)N(=O)=O | 1.49 | -5.69 |
| 240 | Anthracene | 120-12-7 | c1ccc2c(c1)cc1c(c2)cccc1 | 4.45 | -2.64 |
| 241 | Phenanthrene | 85-01-8 | c1ccc2c(c1)c1ccccc1cc2 | 4.46 | -2.75 |
| 242 | Fluoranthene | 206-44-0 | c1ccc2c(c1)c1cccc3c1c2ccc3 | 5.16 | -3.43 |
| 243 | Pyrene | 129-00-0 | c1cc2ccc3c4c2c(c1)ccc4ccc3 | 4.88 | -3.30 |
| 244 | Fluorene | 86-73-7 | c1ccc2c(c1)Cc1c2cccc1 | 4.18 | -2.40 |
| 245 | Acenaphthene | 83-32-9 | c1cc2cccc3c2c(c1)CC3 | 3.92 | -2.12 |
| 246 | phenol | 108-95-2 | Oc1ccccc1 | 1.46 | -4.86 |
| 247 | carbon disulfide | 75-15-0 | S=C=S | 1.94 | -0.22 |
| 248 | acetylene | 74-86-2 | C#C | 0.37 | -0.04 |
| 249 | methanal | 50-00-0 | C=O | 0.35 | -4.85 |
| 250 | 1-Propanethiol | 107-03-9 | CCCS | 1.81 | -0.77 |
| 251 | 1-Butanethiol | 109-79-5 | CCCCS | 2.28 | -0.72 |
| 252 | 1-Pentanethiol | 110-66-7 | CCCCCS | **2.92** | -0.30 |
| 253 | 1-Hexanethiol | 111-31-9 | CCCCCCS | **3.49** | **-0.44** |
| 254 | 2-Pentanol | 6032-29-7 | CCCC(O)C | 1.19 | -3.21 |
| 255 | 2-Hexanol | 626-93-7 | CCCCC(O)C | 1.76 | -2.99 |
| 256 | 3-Hexanol | 623-37-0 | CCCC(CC)O | 1.65 | -2.78 |
| 257 | 2-Heptanol | 543-49-7 | CCCCCC(O)C | 2.31 | -2.64 |
| 258 | 4-Heptanol | 589-55-9 | CCCC(CCC)O | 2.22 | **-3.00** |
| 259 | 4-Octanol | 589-62-8 | CCCCC(CCC)O | 2.68 | **-2.86** |
| 260 | 2,2,2-Trifluoroethanol | 75-89-8 | OCC(F)(F)F | 0.41 | -3.14 |
| 261 | 1,1,1-Trifluoro-2-propanol | 374-01-6 | CC(C(F)(F)F)O | 0.71 | -3.04 |
| 262 | 2,2,3,3-Tetrafluoro-1-propanol | 76-37-9 | OCC(C(F)F)(F)F | **1.39** | -3.58 |
| 263 | 2,2,3,3,3-Pentafluoro-1-propanol | 422-05-9 | OCC(C(F)(F)F)(F)F | 1.23 | -3.03 |
| 264 | 1,1,1,3,3,3-Hexafluoro-2-propanol | 920-66-1 | OC(C(F)(F)F)C(F)(F)F | 1.66 | -2.75 |
| 265 | 1,1,1,3,3,3-Hexafluoro-2-methyl-2-propanol | 1515-14-6 | CC(C(F)(F)F)(C(F)(F)F)O | **2.16** | **-1.63** |
| 266 | 2,2,3,4,4,4-Hexafluoro-1-butanol | 382-31-0 | OCC(C(C(F)(F)F)F)(F)F | **1.99** | **-2.80** |
| 267 | 2,2,3,3,4,4,4-Heptafluoro-1-butanol | 375-01-9 | OCC(C(C(F)(F)F)(F)F)(F)F | 1.94 | **-1.60** |
| 268 | 2-Butanol | 78-92-2 | CCC(O)C | 0.61 | -3.42 |
| 269 | Triethyl phosphate | 78-40-0 | CCOP(=O)(OCC)OCC | 0.8 | -5.82 |
| 270 | 1,2-Dihydroxybenzene / Catechol | 120-80-9 | Oc1ccccc1O | 0.88 | -7.30 |
| 271 | PCB 28 | 7012-37-5 | Clc1ccc(cc1)c1ccc(cc1Cl)Cl | 5.62 | -2.08 |
| 272 | PCB 31 | 16606-02-3 | Clc1ccc(cc1)c1cc(Cl)ccc1Cl | 5.69 | -2.10 |
| 273 | PCB 44 | 41464-39-5 | Clc1ccc(c(c1)c1cccc(c1Cl)Cl)Cl | 5.81 | -2.23 |
| 274 | PCB 49 | 41464-40-8 | Clc1ccc(c(c1)Cl)c1cc(Cl)ccc1Cl | 6.22 | -2.06 |
| 275 | PCB 52 | 35693-99-3 | Clc1ccc(cc1c1cc(Cl)ccc1Cl)Cl | 6.09 | -2.08 |
| 276 | PCB 99 | 38380-01-7 | Clc1ccc(c(c1)Cl)c1cc(Cl)c(cc1Cl)Cl | 7.21 | -2.49 |
| 277 | PCB 101 | 37680-73-2 | Clc1ccc(c(c1)c1cc(Cl)c(cc1Cl)Cl)Cl | 6.8 | -2.43 |
| 278 | PCB 105 | 32598-14-4 | Clc1ccc(cc1Cl)c1ccc(c(c1Cl)Cl)Cl | 6.79 | -1.93 |
| 279 | PCB 110 | 38380-03-9 | Clc1cc(ccc1Cl)c1c(Cl)ccc(c1Cl)Cl | 6.22 | **-2.84** |
| 280 | PCB 118 | 31508-00-6 | Clc1cc(Cl)c(cc1c1ccc(c(c1)Cl)Cl)Cl | 7.12 | -1.92 |
| 281 | PCB 128 | 38380-07-3 | Clc1c(ccc(c1Cl)Cl)c1ccc(c(c1Cl)Cl)Cl | 7.31 | -3.27 |
| 282 | PCB 138 | 35065-28-2 | Clc1cc(Cl)c(cc1c1ccc(c(c1Cl)Cl)Cl)Cl | 7.44 | -3.06 |
| 283 | PCB 149 | 38380-04-0 | Clc1cc(Cl)c(cc1c1c(Cl)ccc(c1Cl)Cl)Cl | 7.28 | -1.24 |
| 284 | PCB 151 | 52663-63-5 | Clc1ccc(c(c1)c1c(Cl)c(Cl)cc(c1Cl)Cl)Cl | **6.40** | -2.61 |
| 285 | PCB 153 | 35065-27-1 | Clc1cc(Cl)c(cc1c1cc(Cl)c(cc1Cl)Cl)Cl | 7.75 | -3.02 |
| 286 | PCB 156 | 38380-08-4 | Clc1ccc(cc1Cl)c1cc(Cl)c(c(c1Cl)Cl)Cl | 7.6 | -2.22 |
| 287 | PCB 170 | 35065-30-6 | Clc1c(Cl)ccc(c1Cl)c1cc(Cl)c(c(c1Cl)Cl)Cl | **7.20** | -3.43 |
| 288 | PCB 180 | 35065-29-3 | Clc1cc(Cl)c(cc1c1cc(Cl)c(c(c1Cl)Cl)Cl)Cl | **7.13** | -3.38 |
| 289 | PCB 187 | 52663-68-0 | Clc1cc(Cl)c(cc1c1c(Cl)c(Cl)cc(c1Cl)Cl)Cl | **6.89** | **-2.89** |
| 290 | PCB 188 | 74487-85-7 | Clc1cc(Cl)c(c(c1)Cl)c1c(Cl)c(Cl)cc(c1Cl)Cl | **6.70** | **-2.68** |
| 291 | PCB 194 | 35694-08-7 | Clc1cc(c(c(c1Cl)Cl)Cl)c1cc(Cl)c(c(c1Cl)Cl)Cl | 8.68 | -3.38 |
| 292 | PCB 209 | 2051-24-3 | Clc1c(c2c(Cl)c(Cl)c(c(c2Cl)Cl)Cl)c(Cl)c(c(c1Cl)Cl)Cl | 8.27 | -0.41 |
| 293 | Naphthalene | 91-20-3 | c1ccc2c(c1)cccc2 | 3.3 | -1.74 |
| 294 | Benz[a]anthracene | 56-55-3 | c1ccc2c(c1)cc1c(c2)ccc2c1cccc2 | 5.76 | -3.30 |
| 295 | Chrysene | 218-01-9 | c1ccc2c(c1)c1ccc3c(c1cc2)cccc3 | 5.81 | -3.66 |
| 296 | Benzo[a]pyrene | 50-32-8 | c1ccc2c(c1)c1ccc3c4c1c(c2)ccc4ccc3 | 6.13 | -4.72 |
| 297 | Benzo[k]fluoranthene | 207-08-9 | c1ccc2c(c1)cc1c(c2)c2c3c1cccc3ccc2 | **6.21** | **-5.56** |
| 298 | Teflurane | 124-72-1 | FC(C(F)(F)F)Br | **2.13** | 0.38 |
| 299 | 2-[Chloro(fluoro)methoxy]-1,1,1,2-tetrafluoroethane | 56885-28-0 | FC(OC(C(F)(F)F)F)Cl | **1.99** | **0.17** |
| 300 | 1,1,1,2,3,4,4,4-Octafluorobutane | 75995-72-1 | FC(C(F)(F)F)C(C(F)(F)F)F | **2.29** | **1.00** |
| 301 | 1,1,1,2,2,3,3,4,4-nonafluorobutane | 375-17-7 | FC(C(C(C(F)(F)F)(F)F)(F)F)F | **2.90** | **2.63** |
| 302 | Sulfur hexafluoride | 2551-62-4 | FS(F)(F)(F)(F)F | 1.68 | 2.27 |
| 303 | 2-(Chlorodifluoromethoxy)-1,1,1-trifluoroethane | 33018-78-9 | FC(OCC(F)(F)F)(Cl)F | **2.58** | **1.39** |
| 304 | 3,4-Dichlorophenol | 95-77-2 | Oc1ccc(c(c1)Cl)Cl | 3.33 | **-4.64** |
| 305 | 1-Nitronaphthalene | 86-57-7 | O=N(=O)c1cccc2c1cccc2 | 3.19 | -4.13 |

## **Table S6**. Showing chemicals with their experimental and estimated values (bold format) of logK_ow_ and logK_aw_ from EPI Suite used to formulate tp-LFER model equation of logK_pw_.

| **S. No** | **Chemicals** | **CAS-RN** | **SMILES** | **logK_ow_** | **logK_aw_** |
| --- | --- | --- | --- | --- | --- |
| 1 | n-hexane | 110-54-3 | CCCCCC | 3.9 | 1.88 |
| 2 | n-heptane | 142-82-5 | CCCCCCC | 4.66 | 1.92 |
| 3 | n-octane | 111-65-9 | CCCCCCCC | 5.18 | 2.13 |
| 4 | 2,2,4-trimethylpentane | 540-84-1 | CC(CC(C)(C)C)C | **4.65** | 2.10 |
| 5 | cyclohexane | 110-82-7 | C1CCCCC1 | 3.44 | 0.80 |
| 6 | tetrachloromethane | 56-23-5 | ClC(Cl)(Cl)Cl | 2.83 | 0.06 |
| 7 | trichloroethene | 79-01-6 | ClC=C(Cl)Cl | 2.42 | -0.39 |
| 8 | tetrachloroethene | 127-18-4 | ClC(=C(Cl)Cl)Cl | 3.4 | -0.13 |
| 9 | tribromomethane | 75-25-2 | BrC(Br)Br | 2.4 | -1.65 |
| 10 | di-n-butyl ether | 142-96-1 | CCCCOCCCC | 3.21 | -0.60 |
| 11 | di-n-pentyl ether | 693-65-2 | CCCCCOCCCCC | **4.33** | **-0.44** |
| 12 | acetone | 67-64-1 | CC(=O)C | -0.24 | -2.84 |
| 13 | 2-octanone | 111-13-7 | CCCCCCC(=O)C | 2.37 | -2.11 |
| 14 | 2-nonanone | 821-55-6 | CCCCCCCC(=O)C | 3.14 | -1.82 |
| 15 | 2-decanone | 693-54-9 | CCCCCCCCC(=O)C | 3.73 | **-1.91** |
| 16 | cyclopentanone | 120-92-3 | O=C1CCCC1 | 0.38 | -3.38 |
| 17 | cyclohexanone | 108-94-1 | O=C1CCCCC1 | 0.81 | -3.43 |
| 18 | ethyl acetate | 141-78-6 | CCOC(=O)C | 0.73 | -2.25 |
| 19 | propyl acetate | 109-60-4 | CCCOC(=O)C | 1.24 | -2.04 |
| 20 | methanol | 67-56-1 | CO | -0.77 | -3.72 |
| 21 | ethanol | 64-17-5 | CCO | -0.31 | -3.68 |
| 22 | 1-propanol | 71-23-8 | CCCO | 0.25 | -3.51 |
| 23 | 2-propanol | 67-63-0 | CC(O)C | 0.05 | -3.47 |
| 24 | 1-butanol | 71-36-3 | CCCCO | 0.88 | -3.44 |
| 25 | tert-butanol | 75-65-0 | CC(O)(C)C | 0.35 | -3.42 |
| 26 | 1-pentanol | 71-41-0 | CCCCCO | 1.51 | -3.27 |
| 27 | 3-pentanol | 584-02-1 | CCC(CC)O | 1.21 | -3.08 |
| 28 | 1-hexanol | 111-27-3 | CCCCCCO | 2.03 | -3.15 |
| 29 | 1-heptanol | 111-70-6 | CCCCCCCO | 2.62 | -3.11 |
| 30 | 4-heptanol | 589-55-9 | CCCC(CCC)O | 2.22 | **-3.00** |
| 31 | 1-octanol | 111-87-5 | CCCCCCCCO | 3 | -2.99 |
| 32 | cyclopentanol | 96-41-3 | OC1CCCC1 | **0.82** | **-4.04** |
| 33 | cyclohexanol | 108-93-0 | C1CCC(CC1)O | 1.23 | -3.74 |
| 34 | cycloheptanol | 502-41-0 | OC1CCCCCC1 | **1.93** | **-3.98** |
| 35 | ethylene glycol | 107-21-1 | OCCO | -1.36 | -5.60 |
| 36 | 2-butoxyethanol | 111-76-2 | CCCCOCCO | 0.83 | -4.18 |
| 37 | benzyl alcohol | 100-51-6 | OCc1ccccc1 | 1.1 | -4.85 |
| 38 | p-xylene | 106-42-3 | Cc1ccc(cc1)C | 3.15 | -0.54 |
| 39 | chlorobenzene | 108-90-7 | Clc1ccccc1 | 2.84 | -0.89 |
| 40 | 1,2-dichlorobenzene | 95-50-1 | Clc1ccccc1Cl | 3.43 | -1.10 |
| 41 | 1,3-dichlorobenzene | 541-73-1 | Clc1cccc(c1)Cl | 3.53 | -0.96 |
| 42 | 1,4-dichlorobenzene | 106-46-7 | Clc1ccc(cc1)Cl | 3.44 | -1.00 |
| 43 | 1,2,3-trichlorobenzene | 87-61-6 | Clc1c(Cl)cccc1Cl | 4.05 | -1.28 |
| 44 | 1,2,4-trichlorobenzene | 120-82-1 | Clc1ccc(c(c1)Cl)Cl | 4.02 | -1.23 |
| 45 | 1,3,5-trichlorobenzene | 108-70-3 | Clc1cc(Cl)cc(c1)Cl | 4.19 | -1.10 |
| 46 | 1,2,3,5-tetrachlorobenzene | 634-90-2 | Clc1cc(Cl)c(c(c1)Cl)Cl | 4.56 | -1.18 |
| 47 | 1,2,4,5-tetrachlorobenzene | 95-94-3 | Clc1cc(Cl)c(cc1Cl)Cl | 4.64 | -1.38 |
| 48 | pentachloro benzene | 608-93-5 | Clc1cc(Cl)c(c(c1Cl)Cl)Cl | 5.17 | -1.53 |
| 49 | hexachlorobenzene | 118-74-1 | Clc1c(Cl)c(Cl)c(c(c1Cl)Cl)Cl | 5.73 | -1.15 |
| 50 | 2,4,5-trichlorotoluene | 6639-30-1 | Clc1cc(Cl)c(cc1C)Cl | 4.56 | -1.20 |
| 51 | 1,4-dibromobenzene | 106-37-6 | Brc1ccc(cc1)Br | 3.79 | -1.43 |
| 52 | 2 ,2',4,6-tetrachlorobiphenyl (PCB 50) | 62796-65-0 | Clc1cc(Cl)c(c(c1)Cl)c1ccccc1Cl | **5.48** | **-2.35** |
| 53 | 2 ,2',5,5'-tetrachlorobiphenyl (PCB 52) | 35693-99-3 | Clc1ccc(cc1c1cc(Cl)ccc1Cl)Cl | 6.09 | -2.08 |
| 54 | 3 ,3',4,5-tetrachlorobiphenyl (PCB 78) | 70362-49-1 | Clc1cccc(c1)c1cc(Cl)c(c(c1)Cl)Cl | **6.19** | **-2.64** |
| 55 | 2 ,2',4,5',6-pentachlorobiphenyl (PCB 103) | 60145-21-3 | Clc1ccc(c(c1)c1c(Cl)cc(cc1Cl)Cl)Cl | **5.91** | **-2.48** |
| 56 | 2 ,2',4,6,6'-pentachlorobiphenyl (PCB 104) | 56558-16-8 | Clc1cc(Cl)c(c(c1)Cl)c1c(Cl)cccc1Cl | **5.83** | **-2.40** |
| 57 | 2 ,2',3,3',6,6'-hexachlorobiphenyl (PCB 136) | 38411-22-2 | Clc1ccc(c(c1c1c(Cl)ccc(c1Cl)Cl)Cl)Cl | 7.12 | -2.44 |
| 58 | 2 ,2',3,4,4',5,6'-heptachlorobiphenyl (PCB 182) | 60145-23-5 | Clc1cc(Cl)c(c(c1)Cl)c1cc(Cl)c(c(c1Cl)Cl)Cl | **6.93** | **-2.94** |
| 59 | phenanthrene | 85-01-8 | c1ccc2c(c1)c1ccccc1cc2 | 4.46 | -2.75 |
| 60 | anthracene | 120-12-7 | c1ccc2c(c1)cc1c(c2)cccc1 | 4.45 | -2.64 |
| 61 | fluoranthene | 206-44-0 | c1ccc2c(c1)c1cccc3c1c2ccc3 | 5.16 | -3.43 |
| 62 | pyrene | 129-00-0 | c1cc2ccc3c4c2c(c1)ccc4ccc3 | 4.88 | -3.30 |
| 63 | benzo[a]anthracene | 56-55-3 | c1ccc2c(c1)cc1c(c2)ccc2c1cccc2 | 5.76 | -3.30 |
| 64 | chrysene | 218-01-9 | c1ccc2c(c1)c1ccc3c(c1cc2)cccc3 | 5.81 | -3.66 |
| 65 | benzo[b]fluoranthene | 205-99-2 | c1ccc2c(c1)c1cc3ccccc3c3c1c2ccc3 | 5.78 | -4.56 |
| 66 | benzo[k]fluoranthene | 207-08-9 | c1ccc2c(c1)cc1c(c2)c2c3c1cccc3ccc2 | **6.21** | **-5.56** |
| 67 | benzo[a]pyrene | 50-32-8 | c1ccc2c(c1)c1ccc3c4c1c(c2)ccc4ccc3 | 6.13 | -4.72 |
| 68 | benzo[ghi]perylene | 191-24-2 | c1cc2ccc3c4c2c(c1)c1cccc2c1c4c(cc3)cc2 | **6.83** | **-6.59** |
| 69 | dibenzo[a,h]anthracene | 53-70-3 | c1ccc2c(c1)c1cc3ccc4c(c3cc1cc2)cccc4 | 6.54 | -5.23 |
| 70 | dibenzo[a,c]anthracene | 215-58-7 | c1ccc2c(c1)cc1c(c2)c2ccccc2c2c1cccc2 | 6.41 | **-6.20** |
| 71 | indenol[1,2,3-cd] pyrene | 193-39-5 | c1ccc2c(c1)c1cc3cccc4c3c3c1c2ccc3cc4 | **6.65** | -4.84 |
| 72 | nitrobenzene | 98-95-3 | O=N(=O)c1ccccc1 | 1.85 | -3.00 |
| 73 | 2-nitrotoluene | 88-72-2 | O=N(=O)c1ccccc1C | 2.3 | -3.28 |
| 74 | quinoline | 91-22-5 | c1ccc2c(c1)nccc2 | 2.03 | -4.16 |
| 75 | diethyl phthalate | 84-66-2 | CCOC(=O)c1ccccc1C(=O)OCC | 2.42 | -4.59 |
| 76 | dibutyl phthalate | 84-74-2 | CCCCOC(=O)c1ccccc1C(=O)OCCCC | 4.5 | -4.12 |
| 77 | phenol | 108-95-2 | c1ccc(cc1)O | 1.46 | -4.86 |
| 78 | 2-methylphenol | 95-48-7 | Cc1ccccc1O | 1.95 | -4.30 |
| 79 | 3-methylphenol | 108-39-4 | Cc1cccc(c1)O | 1.96 | -4.45 |
| 80 | 4-methylphenol | 106-44-5 | Cc1ccc(cc1)O | 1.94 | -4.38 |
| 81 | 2-ethylphenol | 90-00-6 | CCc1ccccc1O | 2.47 | -3.72 |
| 82 | 4-ethylphenol | 123-07-9 | CCc1ccc(cc1)O | 2.58 | -4.49 |
| 83 | 2,6-dimethylphenol | 576-26-1 | Cc1cccc(c1O)C | 2.36 | -3.56 |
| 84 | 2-n-propylphenol | 644-35-9 | CCCc1ccccc1O | 2.93 | **-4.22** |
| 85 | 4-n-propylphenol | 645-56-7 | CCCc1ccc(cc1)O | 3.2 | -4.32 |
| 86 | 4-isopropylphenol | 99-89-8 | CC(c1ccc(cc1)O)C | 2.9 | **-4.46** |
| 87 | 3,4,5-trimethylphenol | 527-54-8 | Oc1cc(C)c(c(c1)C)C | **2.76** | **-5.01** |
| 88 | 4-n-butylphenol | 1638-22-8 | CCCCc1ccc(cc1)O | 3.65 | **-4.35** |
| 89 | 2-sec-butylphenol | 89-72-5 | CCC(c1ccccc1O)C | 3.27 | **-4.21** |
| 90 | 2-tert-butylphenol | 88-18-6 | Oc1ccccc1C(C)(C)C | 3.31 | -2.98 |
| 91 | 4-tert-butylphenol | 98-54-4 | CC(c1ccc(cc1)O)(C)C | 3.31 | -4.30 |
| 92 | 4-tert-amylphenol | 80-46-6 | CCC(c1ccc(cc1)O)(C)C | **3.62** | **-4.30** |
| 93 | 2-phenylphenol | 90-43-7 | Oc1ccccc1c1ccccc1 | 3.09 | -4.36 |
| 94 | 4-phenylphenol | 92-69-3 | Oc1ccc(cc1)c1ccccc1 | 3.2 | **-6.54** |
| 95 | bisphenol A | 80-05-7 | CC(c1ccc(cc1)O)(c1ccc(cc1)O)C | 3.32 | **-10.56** |
| 96 | 2-chlorophenol | 95-57-8 | Oc1ccccc1Cl | 2.15 | -3.33 |
| 97 | 3-chlorophenol | 108-43-0 | Oc1cccc(c1)Cl | 2.5 | -4.84 |
| 98 | 4-chlorophenol | 106-48-9 | Oc1ccc(cc1)Cl | 2.39 | -4.58 |
| 99 | 4-chloro-3-methylphenol | 59-50-7 | Oc1ccc(c(c1)C)Cl | 3.1 | -3.99 |
| 100 | 2,4-dichlorophenol | 120-83-2 | Clc1ccc(c(c1)Cl)O | 3.06 | -3.75 |
| 101 | 2,6-dichlorophenol | 87-65-0 | Clc1cccc(c1O)Cl | 2.75 | -3.95 |
| 102 | 3,4-dichlorophenol | 95-77-2 | Oc1ccc(c(c1)Cl)Cl | 3.33 | **-4.75** |
| 103 | 2,4,5-trichlorophenol | 95-95-4 | Clc1cc(Cl)c(cc1O)Cl | 3.72 | -4.17 |
| 104 | 2,4,6-trichlorophenol | 88-06-2 | Clc1cc(Cl)c(c(c1)Cl)O | 3.69 | -3.97 |
| 105 | 3,4,5-trichlorophenol | 609-19-8 | Oc1cc(Cl)c(c(c1)Cl)Cl | 4.01 | **-5.08** |
| 106 | 2,3,4,5-tetrachlorophenol | 4901-51-3 | Clc1c(O)cc(c(c1Cl)Cl)Cl | 4.21 | **-4.26** |
| 107 | pentachlorophenol | 87-86-5 | Clc1c(O)c(Cl)c(c(c1Cl)Cl)Cl | 5.12 | -5.99 |
| 108 | 4-fluorophenol | 371-41-5 | Oc1ccc(cc1)F | 1.77 | -4.53 |
| 109 | 4-bromophenol | 106-41-2 | Oc1ccc(cc1)Br | 2.59 | -5.20 |
| 110 | 4-iodophenol | 540-38-5 | Oc1ccc(cc1)I | 2.91 | **-5.41** |
| 111 | 2-nitrophenol | 88-75-5 | O=N(=O)c1ccccc1O | 1.79 | -3.27 |
| 112 | 3-nitrophenol | 554-84-7 | Oc1cccc(c1)N(=O)=O | 2 | -7.08 |
| 113 | 4-nitrophenol | 100-02-7 | Oc1ccc(cc1)N(=O)=O | 1.91 | -7.76 |
| 114 | 2,4-dinitrophenol | 51-28-5 | O=N(=O)c1ccc(c(c1)N(=O)=O)O | 1.67 | -5.45 |
| 115 | 2,6-dinitrophenol | 573-56-8 | Oc1c(cccc1N(=O)=O)N(=O)=O | 1.37 | **-6.70** |
| 116 | 3,4-dinitrophenol | 577-71-9 | Oc1ccc(c(c1)N(=O)=O)N(=O)=O | **1.68** | **-9.02** |
| 117 | 2-sec-butyl-4,6-dinitrophenol | 88-85-7 | CCC(c1cc(cc(c1O)N(=O)=O)N(=O)=O)C | 3.56 | -4.72 |
| 118 | 4-cyanophenol | 767-00-0 | N#Cc1ccc(cc1)O | 1.6 | **-7.38** |
| 119 | aniline | 62-53-3 | Nc1ccccc1 | 0.9 | -4.07 |
| 120 | 3,4-dimethylaniline | 95-64-7 | Nc1ccc(c(c1)C)C | 1.84 | -4.11 |
| 121 | 3-nitroaniline | 99-09-2 | Nc1cccc(c1)N(=O)=O | 1.37 | -6.48 |
| 122 | N, N-dimethylaniline | 121-69-7 | CN(c1ccccc1)C | 2.31 | -2.63 |
| 123 | estrone | 53-16-7 | Oc1ccc2c(c1)CC[C@@H]1[C@@H]2CC[C@]2([C@H]1CCC2=O)C | 3.13 | **-10.42** |
| 124 | -estradiol | 50-28-2 | Oc1ccc2c(c1)CC[C@@H]1[C@@H]2CC[C@]2([C@H]1CC[C@@H]2O)C | 4.01 | **-11.31** |
| 125 | estriol | 50-27-1 | Oc1ccc2c(c1)CC[C@@H]1[C@@H]2CC[C@]2([C@H]1C[C@H]([C@@H]2O)O)C | 2.45 | **-17.17** |
| 126 | progesterone | 57-83-0 | O=C1CC[C@]2(C(=C1)CC[C@@H]1[C@@H]2CC[C@]2([C@H]1CC[C@@H]2C(=O)C)C)C | 3.87 | **-10.87** |
| 127 | diazepam | 439-14-5 | Clc1ccc2c(c1)C(=NCC(=O)N2C)c1ccccc1 | 2.82 | **-8.59** |
| 128 | lidocaine | 137-58-6 | CCN(CC(=O)Nc1c(C)cccc1C)CC | 2.44 | **-7.52** |
| 129 | diclofenac | 15307-86-5 | OC(=O)Cc1ccccc1Nc1c(Cl)cccc1Cl | 4.51 | **-9.14** |
| 130 | ibuprofen | 15687-27-1 | CC(Cc1ccc(cc1)C(C(=O)O)C)C | 3.97 | **-5.68** |
| 131 | salicylic acid | 69-72-7 | OC(=O)c1ccccc1O | 2.26 | -6.51 |

## **Table S7**. Showing chemicals with their estimated values of logK_ow_ and logK_aw_ from EPI Suite and estimated logK_lw_ from tp-LFER model equation in comparison to experimental logK_lw_.

| **S. No** | **Chemicals** | **logK_ow_ (EPI Suite)** | **logK_aw_ (EPI Suite)** | **logK_lw_ (tp-LFER)** | **logK_lw_ (experimental)** |
| --- | --- | --- | --- | --- | --- |
| 1 | 1,1,1,2-Tetrachloroethane | 2.93 | -1.20 | 2.91 | 2.85 |
| 2 | 1,1,1-Trichloroethane | 2.68 | -0.75 | 2.67 | 2.66 |
| 3 | 1,1,2,2-Tetrachloroethane | 2.19 | -1.20 | 2.10 | 2.46 |
| 4 | 1,1,2-Trichloroethane | 2.01 | -0.75 | 1.93 | 2.09 |
| 5 | 1,1-Dichloro-1-fluoroethane | 2.37 | 0.00 | 2.38 | 1.34 |
| 6 | 1,1-Dichloroethane | 1.76 | -0.30 | 1.68 | 1.74 |
| 7 | 1,1-Dichloroethene | 2.12 | 0.12 | 2.11 | 2.19 |
| 8 | 1,2,3-Trimethylbenzene | 3.63 | -0.52 | 3.73 | 3.32 |
| 9 | 1,2,4-Trifluorobenzene | 2.59 | -0.45 | 2.59 | 2.67 |
| 10 | 1,2,4-Trimethylbenzene | 3.63 | -0.52 | 3.73 | 3.43 |
| 11 | 1,2-Dibromoethane | 2.01 | -1.27 | 1.89 | 1.67 |
| 12 | 1,2-Dichlorobenzene | 3.28 | -0.91 | 3.32 | 3.71 |
| 13 | 1,2-Dichloroethane | 1.83 | -0.30 | 1.76 | 1.60 |
| 14 | 1,2-Dichloropropane | 2.25 | -0.17 | 2.23 | 1.99 |
| 15 | 1,2-Difluorobenzene | 2.39 | -0.51 | 2.36 | 2.62 |
| 16 | 1,3,5-Trifluorobenzene | 2.59 | -0.45 | 2.59 | 2.93 |
| 17 | 1,3,5-Trimethylbenzene | 3.63 | -0.52 | 3.73 | 3.49 |
| 18 | 1,3-Dichlorobenzene | 3.28 | -0.91 | 3.32 | 3.84 |
| 19 | 1,4-Difluorobenzene | 2.39 | -0.51 | 2.36 | 2.58 |
| 20 | 1-Bromo-2-chloroethane | 1.92 | -0.78 | 1.83 | 2.07 |
| 21 | 1-Butanol | 0.84 | -3.38 | 0.46 | 0.02 |
| 22 | 1-Chlorobutane | 2.56 | -0.10 | 2.58 | 2.78 |
| 23 | 1-Chloropentane | 3.05 | 0.03 | 3.13 | 3.48 |
| 24 | 1-Chloropropane | 2.07 | -0.22 | 2.03 | 2.22 |
| 25 | 1-Hexanol | 1.82 | -3.13 | 1.56 | 1.36 |
| 26 | 1-Methoxy-2-propanol | -0.49 | -5.64 | -1.16 | -1.53 |
| 27 | 1-Nitropropane | 0.95 | -2.51 | 0.64 | 0.97 |
| 28 | 1-Pentanol | 1.33 | -3.26 | 1.01 | 0.54 |
| 29 | 1-Propanol | 0.35 | -3.50 | -0.09 | -0.48 |
| 30 | 2,2,4-Trimethylpentane | 4.09 | 2.10 | 4.41 | 4.64 |
| 31 | 2,2-Dichloro-1,1,1-trifluoroethane | 2.17 | 0.60 | 2.20 | 1.81 |
| 32 | 2,2-Dimethylbutane | 3.18 | 1.85 | 3.40 | 3.79 |
| 33 | 2,3,4-Trimethylpentane | 4.05 | 2.10 | 4.37 | 5.05 |
| 34 | 2-Butoxyethanol | 0.57 | -5.39 | 0.03 | -0.21 |
| 35 | 2-Chloropropane | 2 | -0.22 | 1.95 | 2.03 |
| 36 | 2-Ethoxyethanol | -0.42 | -5.64 | -1.08 | -1.27 |
| 37 | 2-Fluoropropane | 1.68 | 0.08 | 1.62 | 1.38 |
| 38 | 2-Heptanone | 1.73 | -2.19 | 1.52 | 1.81 |
| 39 | 2-Hexanone | 1.24 | -2.32 | 0.97 | 1.19 |
| 40 | 2-Isopropoxyethanol | 0 | -5.51 | -0.61 | -1.16 |
| 41 | 2-Methoxyethanol | -0.91 | -5.76 | -1.63 | -1.67 |
| 42 | 2-Methyl-1-propanol | 0.77 | -3.38 | 0.38 | -0.06 |
| 43 | 2-Methyl-2-propanol | 0.73 | -3.38 | 0.34 | -0.73 |
| 44 | 2-Methylpentane | 3.21 | 1.85 | 3.43 | 3.99 |
| 45 | 2-Nitropropane | 0.87 | -2.51 | 0.55 | 0.61 |
| 46 | 2-Pentanone | 0.75 | -2.44 | 0.43 | 0.65 |
| 47 | 2-Propanol | 0.28 | -3.50 | -0.16 | -0.82 |
| 48 | 3-Methyl-1-butanol | 1.26 | -3.26 | 0.93 | 0.41 |
| 49 | 3-Methylhexane | 3.71 | 1.98 | 3.99 | 4.61 |
| 50 | 3-Methylpentane | 3.21 | 1.85 | 3.43 | 4.07 |
| 51 | 3-Pentanone | 0.75 | -2.44 | 0.43 | 0.56 |
| 52 | 4-Methyl-2-pentanone | 1.16 | -2.32 | 0.89 | 1.05 |
| 53 | Acetone | -0.24 | -2.68 | -0.68 | -0.32 |
| 54 | Allylbenzene | 3.39 | -0.49 | 3.47 | 2.96 |
| 55 | Benzene | 1.99 | -0.65 | 1.91 | 2.12 |
| 56 | Bromochloromethane | 1.43 | -0.90 | 1.28 | 1.49 |
| 57 | 2-Butanone | 0.26 | -2.56 | -0.12 | 0.18 |
| 58 | Butane | 2.31 | 1.61 | 2.42 | 3.03 |
| 59 | Butyl acetate | 1.85 | -1.77 | 1.68 | 1.59 |
| 60 | Carbon tetrachloride | 2.44 | 0.02 | 2.46 | 3.18 |
| 61 | 1,2-Dichlorotetrafluoroethane | 2.78 | 1.80 | 2.95 | 3.00 |
| 62 | 1,1,2,2,3,3,4,4-Octafluorobutane | 2.28 | 3.25 | 2.50 | 2.50 |
| 63 | 1,1,2,2,3,3-Hexafluoropropane | 1.61 | 2.53 | 1.71 | 1.49 |
| 64 | 1,1,2,2-Tetrafluoroethane | 0.94 | 1.80 | 0.92 | 0.92 |
| 65 | 1,1-Difluoroethane | 1.13 | 1.21 | 1.09 | 0.91 |
| 66 | Halothane | 2.26 | 0.12 | 2.26 | 2.28 |
| 67 | 1,1,1,2-Tetrafluoroethane | 1.68 | 1.80 | 1.74 | 1.03 |
| 68 | Fluroxene | 1.33 | 0.40 | 1.26 | 1.26 |
| 69 | Carbon tetrafluoride | 1.19 | 2.28 | 1.23 | 1.12 |
| 70 | 1,3-Difluoropropane | 1.7 | 1.33 | 1.73 | 0.66 |
| 71 | Enflurane | 2.06 | -0.28 | 2.02 | 2.03 |
| 72 | Isoflurane | 1.51 | -0.28 | 1.41 | 1.99 |
| 73 | Desflurane | 1.2 | 0.47 | 1.12 | 1.88 |
| 74 | Sevoflurane | 1.75 | 0.89 | 1.75 | 1.87 |
| 75 | Chlorobenzene | 2.64 | -0.78 | 2.62 | 2.73 |
| 76 | Chlorodibromomethane | 1.7 | -1.84 | 1.51 | 2.12 |
| 77 | Chloroethane | 1.58 | -0.34 | 1.48 | 1.58 |
| 78 | Chloroform | 1.52 | -0.87 | 1.38 | 2.02 |
| 79 | cis-1,2-Dichloroethene | 1.98 | 0.12 | 1.96 | 1.59 |
| 80 | Cycloheptane | 3.67 | 1.15 | 3.89 | 4.82 |
| 81 | Cyclohexane | 3.18 | 1.03 | 3.34 | 4.05 |
| 82 | Cyclopentane | 2.68 | 0.90 | 2.78 | 3.50 |
| 83 | Cyclopropane | 1.7 | 0.66 | 1.68 | 1.73 |
| 84 | Decane | 5.25 | 2.34 | 5.71 | 6.39 |
| 85 | Dibromethane | 1.52 | -1.39 | 1.35 | 1.76 |
| 86 | Dichloromethane | 1.34 | -0.42 | 1.21 | 1.28 |
| 87 | Diethyl ether | 1.05 | -1.20 | 0.84 | 0.79 |
| 88 | Difluoromethane | 0.71 | 1.09 | 0.62 | 0.53 |
| 89 | Divinyl ether | 1.68 | 0.20 | 1.63 | 1.69 |
| 90 | Ethane | 1.32 | 1.36 | 1.31 | 1.73 |
| 91 | Ethanol | -0.14 | -3.63 | -0.64 | -1.14 |
| 92 | Ethene | 1.27 | 0.61 | 1.21 | 0.84 |
| 93 | Ethyl acetate | 0.86 | -2.01 | 0.58 | 0.46 |
| 94 | Ethyl tert-butyl ether | 1.92 | -0.95 | 1.82 | 1.37 |
| 95 | Ethyl tert-pentyl ether | 2.41 | -0.83 | 2.36 | 1.93 |
| 96 | Ethylbenzene | 3.03 | -0.48 | 3.07 | 3.14 |
| 97 | Fluorobenzene | 2.19 | -0.58 | 2.14 | 2.41 |
| 98 | Fluoroethane | 1.26 | -0.04 | 1.15 | 0.62 |
| 99 | Fluorochloromethane | 1.03 | 0.33 | 0.92 | 0.77 |
| 100 | Heptane | 3.78 | 1.98 | 4.06 | 4.71 |
| 101 | Hexafluoro benzene | 3.2 | -0.25 | 3.27 | 2.45 |
| 102 | Hexane | 3.29 | 1.85 | 3.52 | 4.11 |
| 103 | Isobutyl acetate | 1.77 | -1.77 | 1.60 | 1.60 |
| 104 | Isopentyl acetate | 2.26 | -1.64 | 2.14 | 2.11 |
| 105 | Isopropyl acetate | 1.28 | -1.89 | 1.05 | 1.02 |
| 106 | Isopropylbenzene | 3.45 | -0.36 | 3.54 | 3.52 |
| 107 | Methoxyflurane | 2.02 | -1.63 | 1.88 | 2.11 |
| 108 | Methane | 0.78 | 1.24 | 0.71 | 0.75 |
| 109 | Methanol | -0.63 | -3.75 | -1.18 | -1.95 |
| 110 | Methyl acetate | 0.37 | -2.14 | 0.03 | -0.02 |
| 111 | Methyl chloride | 1.09 | -0.47 | 0.93 | 0.80 |
| 112 | Methylcyclopentane | 3.1 | 1.03 | 3.25 | 4.00 |
| 113 | Methylpentafluorobenzene | 3.54 | -0.27 | 3.65 | 3.27 |
| 114 | m-Methylstyrene | 3.44 | -0.90 | 3.49 | 3.23 |
| 115 | m-Xylene | 3.09 | -0.56 | 3.13 | 3.16 |
| 116 | Nonane | 4.76 | 2.22 | 5.16 | 5.82 |
| 117 | o-Xylene | 3.09 | -0.56 | 3.13 | 3.12 |
| 118 | p-Xylene | 3.09 | -0.56 | 3.13 | 3.16 |
| 119 | Pentachloroethane | 3.11 | -1.66 | 3.08 | 2.93 |
| 120 | Pentafluorobenzene | 2.99 | -0.31 | 3.04 | 2.31 |
| 121 | Pentane | 2.8 | 1.73 | 2.97 | 3.49 |
| 122 | Pentyl acetate | 2.34 | -1.64 | 2.23 | 2.11 |
| 123 | p-Methylstyrene | 3.44 | -0.90 | 3.49 | 3.21 |
| 124 | Propane | 1.81 | 1.48 | 1.86 | 2.35 |
| 125 | Propyl acetate | 1.36 | -1.89 | 1.14 | 1.02 |
| 126 | Propylbenzene | 3.52 | -0.36 | 3.62 | 3.65 |
| 127 | Tetrachloroethene | 2.97 | -0.16 | 3.03 | 3.57 |
| 128 | Toluene | 2.54 | -0.61 | 2.52 | 2.67 |
| 129 | trans-1,2-Dichloroethene | 1.98 | 0.12 | 1.96 | 2.06 |
| 130 | Trichloroethene | 2.47 | -0.02 | 2.49 | 2.80 |
| 131 | tridecane | 6.73 | 2.71 | 7.37 | 8.16 |
| 132 | triethylamine | 1.51 | -2.45 | 1.26 | 1.04 |
| 133 | undecane | 5.74 | 2.47 | 6.26 | 7.03 |
| 134 | alpha-pinene | 4.27 | 0.65 | 4.51 | 4.58 |
| 135 | 1,2-dimethoxyethane | -0.21 | -3.25 | -0.69 | -0.33 |
| 136 | 1,4-dioxane | -0.32 | -3.61 | -0.83 | -0.24 |
| 137 | 1-heptanol | 2.31 | -3.01 | 2.11 | 1.84 |
| 138 | 2-methylpyridine | 1.35 | -3.49 | 1.02 | 0.61 |
| 139 | 3-methylpyridine | 1.35 | -3.49 | 1.02 | 0.84 |
| 140 | 4-methylpyridine | 1.35 | -3.49 | 1.02 | 0.82 |
| 141 | benzyl alcohol | 1.08 | -5.04 | 0.61 | 0.12 |
| 142 | bromobenzene | 2.88 | -1.05 | 2.87 | 3.27 |
| 143 | butyl formate | 1.3 | -1.63 | 1.09 | 1.46 |
| 144 | butyl propanoate | 2.34 | -1.64 | 2.23 | 2.35 |
| 145 | butylbenzene | 4.01 | -0.24 | 4.17 | 4.13 |
| 146 | 1,1-difluoro-2-chloroethene | 1.6 | 0.83 | 1.58 | 1.63 |
| 147 | 1-chloro-2,2,2-trifluoroethane | 1.99 | 1.05 | 2.03 | 1.34 |
| 148 | bis-(2,2,2-trifluoroethyl)ether | 1.88 | 0.59 | 1.88 | 1.92 |
| 149 | cyclohexene | 2.96 | 0.44 | 3.06 | 3.33 |
| 150 | cyclopentanone | 0.63 | -2.79 | 0.27 | 0.39 |
| 151 | difluorochloromethane | 0.89 | 0.63 | 0.79 | 0.72 |
| 152 | diisopropyl ether | 1.88 | -0.95 | 1.77 | 1.29 |
| 153 | dimethoxymethane | -0.19 | -2.68 | -0.63 | 0.18 |
| 154 | dimethyl ether | 0.07 | -1.44 | -0.26 | 0.13 |
| 155 | dimethylacetamide | -0.49 | -5.65 | -1.16 | -1.25 |
| 156 | dimethylformamide | -0.93 | -5.51 | -1.63 | -1.57 |
| 157 | di-n-butyl ether | 3.01 | -0.71 | 3.03 | 3.14 |
| 158 | dodecane | 6.23 | 2.59 | 6.81 | 7.59 |
| 159 | ethyl formate | 0.32 | -1.88 | -0.01 | 0.16 |
| 160 | ethyl propanoate | 1.36 | -1.89 | 1.14 | 1.13 |
| 161 | iodoethane | 2.08 | -0.52 | 2.02 | 1.85 |
| 162 | fluorotrichloromethane | 2.13 | 0.33 | 2.13 | 2.28 |
| 163 | methyl formate | -0.17 | -2.00 | -0.56 | -0.48 |
| 164 | methylcyclohexane | 3.59 | 1.15 | 3.80 | 4.54 |
| 165 | nitroethane | 0.45 | -2.63 | 0.08 | 0.45 |
| 166 | nitromethane | -0.04 | -2.76 | -0.47 | -0.02 |
| 167 | N, N-dimethylaniline | 2.17 | -2.45 | 1.99 | 2.29 |
| 168 | pentadecane | 7.71 | 2.96 | 8.46 | 9.31 |
| 169 | piperidine | 1.19 | -3.14 | 0.86 | 1.01 |
| 170 | propyl bromide | 2.16 | -0.20 | 2.13 | 2.47 |
| 171 | propyl formate | 0.81 | -1.75 | 0.54 | 0.87 |
| 172 | pyridine | 0.8 | -3.53 | 0.41 | 0.15 |
| 173 | tetradecane | 7.22 | 2.84 | 7.91 | 8.74 |
| 174 | tetrahydrofuran | 0.94 | -2.45 | 0.63 | 0.62 |
| 175 | halo propane | 2.44 | 0.99 | 2.52 | 2.91 |
| 176 | Cyclooctane | 4.16 | 1.27 | 4.44 | 5.34 |
| 177 | Octan-1-ol | 2.81 | -2.89 | 2.67 | 2.46 |
| 178 | Nonan-1-ol | 3.3 | -2.77 | 3.21 | 3.01 |
| 179 | Hexanal | 1.8 | -2.06 | 1.61 | 1.92 |
| 180 | Heptanal | 2.29 | -1.93 | 2.16 | 2.46 |
| 181 | Octanal | 2.78 | -1.81 | 2.71 | 3.01 |
| 182 | Nonanal / n-Nonyl Aldehyde | 3.27 | -1.69 | 3.25 | 3.42 |
| 183 | 1-Chloroheptane | 4.03 | 0.27 | 4.22 | 4.37 |
| 184 | 1-Chlorooctane | 4.52 | 0.40 | 4.77 | 4.95 |
| 185 | 1-Hexene | 3.15 | 1.17 | 3.32 | 3.51 |
| 186 | 1-Heptene | 3.64 | 1.30 | 3.86 | 4.04 |
| 187 | 1-Octene | 4.13 | 1.42 | 4.41 | 4.60 |
| 188 | 1-Nonene | 4.62 | 1.54 | 4.96 | 5.15 |
| 189 | 1-Decene | 5.12 | 1.67 | 5.52 | 5.64 |
| 190 | 1,2,4-Trichlorobenzene | 3.93 | -1.04 | 4.03 | 4.17 |
| 191 | Di-n-propyl ether | 2.03 | -0.95 | 1.94 | 2.06 |
| 192 | Dipentyl ether | 4 | -0.46 | 4.14 | 4.16 |
| 193 | 2-octanone | 2.22 | -2.07 | 2.07 | 2.31 |
| 194 | 2-nonanone | 2.71 | -1.95 | 2.62 | 2.79 |
| 195 | 1-Nitrobutane | 1.44 | -2.39 | 1.19 | 1.53 |
| 196 | 1-Nitrohexane | 2.42 | -2.14 | 2.29 | 2.58 |
| 197 | 4-Ethylpyridine | 1.84 | -3.37 | 1.56 | 1.14 |
| 198 | 1-Chloro-4-nitrobenzene | 2.46 | -3.18 | 2.26 | 2.38 |
| 199 | Nitrobenzene | 1.81 | -3.05 | 1.55 | 1.92 |
| 200 | 2-Nitrotoluene | 2.36 | -3.01 | 2.16 | 2.40 |
| 201 | 2,6-Dinitrotoluene | 2.18 | -5.41 | 1.80 | 1.94 |
| 202 | 4-Nitroanisole | 1.89 | -4.28 | 1.56 | 2.32 |
| 203 | 1,4-Dimethoxybenzene | 2.15 | -3.10 | 1.92 | 2.12 |
| 204 | 4-Chlorophenol | 2.16 | -4.76 | 1.82 | 1.51 |
| 205 | Ethyl benzoate | 2.32 | -2.72 | 2.14 | 2.59 |
| 206 | Indole | 2.05 | -4.43 | 1.72 | 1.99 |
| 207 | 2-Ethyl-1-hexanol | 2.73 | -2.89 | 2.58 | 2.04 |
| 208 | 3-Ethyl-3-hexanol | 2.69 | -2.89 | 2.53 | 1.70 |
| 209 | 4-Ethyl-3-hexanol | 2.66 | -2.89 | 2.50 | 1.92 |
| 210 | 3-Ethyl-3-pentanol | 2.2 | -3.01 | 1.98 | 1.13 |
| 211 | 2,4-Dinitrotoluene | 2.18 | -5.41 | 1.80 | 2.34 |
| 212 | 1-fluropropane | 1.76 | 0.08 | 1.71 | 0.98 |
| 213 | Hexachloroethane | 4.03 | -0.76 | 4.15 | 3.71 |
| 214 | Biphenyl | 3.76 | -1.76 | 3.79 | 4.14 |
| 215 | hexadecane | 8.2 | 3.08 | 9.01 | 9.88 |
| 216 | isopropyl bromide | 2.08 | -0.20 | 2.04 | 2.28 |
| 217 | beta-pinene | 4.35 | 0.83 | 4.61 | 4.11 |
| 218 | limonene | 4.83 | 1.20 | 5.17 | 4.17 |
| 219 | Fluoromethane | 0.77 | -0.17 | 0.60 | 0.00 |
| 220 | Tricyclo[5.2.1.0(2,6)]decane | 3.59 | 0.81 | 3.78 | 4.62 |
| 221 | Methyl tert-butyl ether | 1.43 | -1.07 | 1.27 | 0.89 |
| 222 | vinyl chloride | 1.62 | 0.27 | 1.57 | 1.58 |
| 223 | dimethyl sulfoxide | -1.22 | -5.68 | -1.97 | -2.66 |
| 224 | formic acid | -0.46 | -4.51 | -1.05 | -1.69 |
| 225 | 3-carene | 4.61 | 0.65 | 4.89 | 4.71 |
| 226 | vinyl bromide | 1.52 | -0.29 | 1.42 | 1.34 |
| 227 | 4-Chloroaniline | 1.72 | -4.23 | 1.37 | 1.62 |
| 228 | allyl chloride | 1.93 | 0.15 | 1.90 | 1.84 |
| 229 | Styrene | 2.89 | -0.94 | 2.89 | 2.68 |
| 230 | Octane | 4.27 | 2.10 | 4.61 | 5.27 |
| 231 | 3-Chlorophenol | 2.16 | -4.76 | 1.82 | 1.66 |
| 232 | Benzyl acetate | 2.08 | -3.23 | 1.84 | 1.70 |
| 233 | 1-Naphthol | 2.69 | -5.64 | 2.35 | 2.19 |
| 234 | 4-bromophenol | 2.4 | -5.03 | 2.07 | 1.61 |
| 235 | 4-Iodoaniline | 2.24 | -4.74 | 1.91 | 2.19 |
| 236 | N, N-Diethyl aniline | 3.15 | -2.20 | 3.09 | 3.17 |
| 237 | 4-n-Propylphenol | 3.04 | -4.34 | 2.82 | 2.19 |
| 238 | 4-iodophenol | 2.68 | -5.27 | 2.36 | 2.29 |
| 239 | 1,3-Dinitrobenzene | 1.63 | -5.46 | 1.19 | 1.42 |
| 240 | Anthracene | 4.35 | -2.67 | 4.38 | 4.83 |
| 241 | Phenanthrene | 4.35 | -2.67 | 4.38 | 4.80 |
| 242 | Fluoranthene | 4.93 | -3.46 | 4.96 | 5.18 |
| 243 | Pyrene | 4.93 | -3.46 | 4.96 | 5.26 |
| 244 | Fluorene | 4.02 | -2.16 | 4.05 | 4.39 |
| 245 | Acenaphthene | 4.15 | -1.93 | 4.21 | 3.97 |
| 246 | phenol | 1.51 | -4.63 | 1.11 | -0.16 |
| 247 | carbon disulfide | 1.94 | 0.10 | 1.91 | 2.46 |
| 248 | acetylene | 0.5 | 0.00 | 0.32 | -0.63 |
| 249 | methanal | 0.35 | -2.41 | -0.01 | -0.41 |
| 250 | 1-Propanethiol | 1.76 | -0.72 | 1.66 | 1.93 |
| 251 | 1-Butanethiol | 2.25 | -0.59 | 2.20 | 2.53 |
| 252 | 1-Pentanethiol | 2.74 | -0.47 | 2.75 | 3.28 |
| 253 | 1-Hexanethiol | 3.23 | -0.35 | 3.30 | 4.02 |
| 254 | 2-Pentanol | 1.26 | -3.26 | 0.93 | 0.30 |
| 255 | 2-Hexanol | 1.75 | -3.13 | 1.48 | 0.91 |
| 256 | 3-Hexanol | 1.75 | -3.13 | 1.48 | 0.48 |
| 257 | 2-Heptanol | 2.24 | -3.01 | 2.03 | 1.52 |
| 258 | 4-Heptanol | 2.24 | -3.01 | 2.03 | 1.67 |
| 259 | 4-Octanol | 2.73 | -2.89 | 2.58 | 2.42 |
| 260 | 2,2,2-Trifluoroethanol | 0.27 | -2.93 | -0.14 | -0.55 |
| 261 | 1,1,1-Trifluoro-2-propanol | 0.69 | -2.81 | 0.33 | -0.05 |
| 262 | 2,2,3,3-Tetrafluoro-1-propanol | 0.63 | -2.51 | 0.29 | -0.10 |
| 263 | 2,2,3,3,3-Pentafluoro-1-propanol | 1.24 | -2.21 | 0.98 | 0.34 |
| 264 | 1,1,1,3,3,3-Hexafluoro-2-propanol | 1.11 | -1.91 | 0.86 | 0.76 |
| 265 | 1,1,1,3,3,3-Hexafluoro-2-methyl-2-propanol | 1.56 | -1.79 | 1.36 | 1.10 |
| 266 | 2,2,3,4,4,4-Hexafluoro-1-butanol | 1.6 | -1.79 | 1.41 | 0.66 |
| 267 | 2,2,3,3,4,4,4-Heptafluoro-1-butanol | 1.91 | -1.49 | 1.77 | 1.17 |
| 268 | 2-Butanol | 0.77 | -3.38 | 0.38 | -0.28 |
| 269 | Triethyl phosphate | 0.87 | -4.61 | 0.41 | 0.22 |
| 270 | 1,2-Dihydroxybenzene / Catechol | 1.03 | -8.61 | 0.31 | 0.79 |
| 271 | PCB 28 | 5.69 | -2.15 | 5.89 | 5.83 |
| 272 | PCB 31 | 5.69 | -2.15 | 5.89 | 5.83 |
| 273 | PCB 44 | 6.34 | -2.28 | 6.60 | 6.01 |
| 274 | PCB 49 | 6.34 | -2.28 | 6.60 | 6.03 |
| 275 | PCB 52 | 6.34 | -2.28 | 6.60 | 6.04 |
| 276 | PCB 99 | 6.98 | -2.41 | 7.29 | 6.52 |
| 277 | PCB 101 | 6.98 | -2.41 | 7.29 | 6.52 |
| 278 | PCB 105 | 6.98 | -2.41 | 7.29 | 6.82 |
| 279 | PCB 110 | 6.98 | -2.41 | 7.29 | 6.53 |
| 280 | PCB 118 | 6.98 | -2.41 | 7.29 | 6.81 |
| 281 | PCB 128 | 7.62 | -2.54 | 7.99 | 6.97 |
| 282 | PCB 138 | 7.62 | -2.54 | 7.99 | 7.00 |
| 283 | PCB 149 | 7.62 | -2.54 | 7.99 | 6.83 |
| 284 | PCB 151 | 7.62 | -2.54 | 7.99 | 6.77 |
| 285 | PCB 153 | 7.62 | -2.54 | 7.99 | 7.03 |
| 286 | PCB 156 | 7.62 | -2.54 | 7.99 | 7.34 |
| 287 | PCB 170 | 8.27 | -2.68 | 8.70 | 7.45 |
| 288 | PCB 180 | 8.27 | -2.68 | 8.70 | 7.49 |
| 289 | PCB 187 | 8.27 | -2.68 | 8.70 | 7.27 |
| 290 | PCB 188 | 8.27 | -2.68 | 8.70 | 7.03 |
| 291 | PCB 194 | 8.91 | -2.81 | 9.39 | 8.01 |
| 292 | PCB 209 | 10.2 | -3.07 | 10.80 | 8.26 |
| 293 | Naphthalene | 3.17 | -1.66 | 3.15 | 3.61 |
| 294 | Benz[a]anthracene | 5.52 | -3.68 | 5.60 | 6.26 |
| 295 | Chrysene | 5.52 | -3.68 | 5.60 | 6.25 |
| 296 | Benzo[a]pyrene | 6.11 | -4.47 | 6.19 | 6.79 |
| 297 | Benzo[k]fluoranthene | 6.11 | -4.47 | 6.19 | 6.84 |
| 298 | Teflurane | 1.95 | 0.87 | 1.97 | 1.80 |
| 299 | 2-[Chloro(fluoro)methoxy]-1,1,1,2-tetrafluoroethane | 0.96 | -0.28 | 0.80 | 2.15 |
| 300 | 1,1,1,2,3,4,4,4-Octafluorobutane | 2.87 | 3.25 | 3.15 | 2.44 |
| 301 | 1,1,1,2,2,3,3,4,4-nonafluorobutane | 2.89 | 3.55 | 3.19 | 2.82 |
| 302 | Sulfur hexafluoride | 1.64 | 2.11 | 1.72 | 1.85 |
| 303 | 2-(Chlorodifluoromethoxy)-1,1,1-trifluoroethane | 2.25 | -0.28 | 2.23 | 2.73 |
| 304 | 3,4-Dichlorophenol | 2.8 | -4.89 | 2.52 | 2.42 |
| 305 | 1-Nitronaphthalene | 2.99 | -4.06 | 2.78 | 3.45 |

## **Table S8**. Showing chemicals with their estimated values of logK_ow_ and logK_aw_ from EPI Suite and estimated logK_pw_ from tp-LFER model equation in comparison to experimental logK_pw_.

| **S. No** | **Chemicals** | **logK_ow_ (EPI Suite)** | **logK_aw_ (EPI Suite)** | **logK_pw_ (tp-LFER)** | **logK_pw_ (experimental)** |
| --- | --- | --- | --- | --- | --- |
| 1 | n-hexane | 3.29 | 1.74 | 3.17 | 3.91 |
| 2 | n-heptane | 3.78 | 1.86 | 3.69 | 4.55 |
| 3 | n-octane | 4.27 | 1.98 | 4.21 | 4.67 |
| 4 | 2,2,4-trimethylpentane | 4.09 | 2.25 | 4.01 | 4.61 |
| 5 | cyclohexane | 3.18 | 1.12 | 3.10 | 3.27 |
| 6 | tetrachloromethane | 2.44 | 0.33 | 2.36 | 2.61 |
| 7 | trichloroethene | 2.47 | -0.23 | 2.40 | 2.43 |
| 8 | tetrachloroethene | 2.97 | 0.09 | 2.94 | 3.08 |
| 9 | tribromomethane | 1.79 | -1.50 | 1.80 | 2.33 |
| 10 | di-n-butyl ether | 3.01 | -0.70 | 3.01 | 2.78 |
| 11 | di-n-pentyl ether | 4.00 | -0.44 | 4.06 | 3.77 |
| 12 | acetone | -0.24 | -2.81 | -0.36 | 0.06 |
| 13 | 2-octanone | 2.22 | -2.15 | 2.24 | 2.42 |
| 14 | 2-nonanone | 2.71 | -2.02 | 2.76 | 2.83 |
| 15 | 2-decanone | 3.20 | -1.91 | 3.28 | 3.16 |
| 16 | cyclopentanone | 0.63 | -3.43 | 0.58 | 0.30 |
| 17 | cyclohexanone | 1.13 | -3.54 | 1.11 | 0.54 |
| 18 | ethyl acetate | 0.86 | -2.09 | 0.78 | 0.46 |
| 19 | propyl acetate | 1.36 | -1.93 | 1.31 | 1.01 |
| 20 | methanol | -0.63 | -3.87 | -0.71 | -0.53 |
| 21 | ethanol | -0.14 | -3.54 | -0.20 | -0.26 |
| 22 | 1-propanol | 0.35 | -3.45 | 0.32 | 0.17 |
| 23 | 2-propanol | 0.28 | -3.44 | 0.25 | -0.04 |
| 24 | 1-butanol | 0.84 | -3.36 | 0.84 | 0.51 |
| 25 | tert-butanol | 0.73 | -3.17 | 0.72 | 0.16 |
| 26 | 1-pentanol | 1.33 | -3.24 | 1.36 | 1.08 |
| 27 | 3-pentanol | 1.26 | -3.24 | 1.28 | 1.00 |
| 28 | 1-hexanol | 1.82 | -3.13 | 1.87 | 1.88 |
| 29 | 1-heptanol | 2.31 | -3.01 | 2.39 | 2.38 |
| 30 | 4-heptanol | 2.24 | -3.00 | 2.32 | 1.70 |
| 31 | 1-octanol | 2.81 | -2.89 | 2.92 | 2.66 |
| 32 | cyclopentanol | 1.15 | -4.04 | 1.19 | 0.52 |
| 33 | cyclohexanol | 1.64 | -3.98 | 1.71 | 1.01 |
| 34 | cycloheptanol | 2.13 | -3.98 | 2.23 | 1.51 |
| 35 | ethylene glycol | -1.20 | -7.18 | -1.24 | -0.79 |
| 36 | 2-butoxyethanol | 0.57 | -4.68 | 0.66 | 0.60 |
| 37 | benzyl alcohol | 1.08 | -4.84 | 1.19 | 1.14 |
| 38 | p-xylene | 3.09 | -0.56 | 3.09 | 2.98 |
| 39 | chlorobenzene | 2.64 | -0.71 | 2.62 | 2.91 |
| 40 | 1,2-dichlorobenzene | 3.28 | -0.94 | 3.31 | 3.64 |
| 41 | 1,3-dichlorobenzene | 3.28 | -0.68 | 3.31 | 3.71 |
| 42 | 1,4-dichlorobenzene | 3.28 | -0.74 | 3.31 | 3.57 |
| 43 | 1,2,3-trichlorobenzene | 3.93 | -1.03 | 4.02 | 4.19 |
| 44 | 1,2,4-trichlorobenzene | 3.93 | -0.84 | 4.02 | 4.20 |
| 45 | 1,3,5-trichlorobenzene | 3.93 | -0.58 | 4.02 | 4.16 |
| 46 | 1,2,3,5-tetrachlorobenzene | 4.57 | -0.94 | 4.71 | 4.77 |
| 47 | 1,2,4,5-tetrachlorobenzene | 4.57 | -0.96 | 4.71 | 4.73 |
| 48 | pentachloro benzene | 5.22 | -1.33 | 5.41 | 5.18 |
| 49 | hexachlorobenzene | 5.86 | -1.31 | 6.10 | 5.64 |
| 50 | 2,4,5-trichlorotoluene | 4.47 | -0.83 | 4.59 | 4.72 |
| 51 | 1,4-dibromobenzene | 3.77 | -1.22 | 3.87 | 4.30 |
| 52 | 2 ,2',4,6-tetrachlorobiphenyl (PCB 50) | 6.34 | -2.35 | 6.66 | 5.92 |
| 53 | 2 ,2',5,5'-tetrachlorobiphenyl (PCB 52) | 6.34 | -2.49 | 6.66 | 5.94 |
| 54 | 3 ,3',4,5-tetrachlorobiphenyl (PCB 78) | 6.34 | -2.64 | 6.66 | 6.53 |
| 55 | 2 ,2',4,5',6-pentachlorobiphenyl (PCB 103) | 6.98 | -2.48 | 7.36 | 6.32 |
| 56 | 2 ,2',4,6,6'-pentachlorobiphenyl (PCB 104) | 6.98 | -2.40 | 7.36 | 6.13 |
| 57 | 2 ,2',3,3',6,6'-hexachlorobiphenyl (PCB 136) | 7.62 | -2.67 | 8.05 | 6.50 |
| 58 | 2 ,2',3,4,4',5,6'-heptachlorobiphenyl (PCB 182) | 8.27 | -2.94 | 8.75 | 6.83 |
| 59 | phenanthrene | 4.35 | -3.30 | 4.56 | 4.95 |
| 60 | anthracene | 4.35 | -3.47 | 4.56 | 5.21 |
| 61 | fluoranthene | 4.93 | -3.98 | 5.22 | 5.58 |
| 62 | pyrene | 4.93 | -4.51 | 5.22 | 5.71 |
| 63 | benzo[a]anthracene | 5.52 | -4.83 | 5.86 | 6.44 |
| 64 | chrysene | 5.52 | -4.91 | 5.86 | 6.40 |
| 65 | benzo[b]fluoranthene | 6.11 | -5.73 | 6.54 | 7.11 |
| 66 | benzo[k]fluoranthene | 6.11 | -5.56 | 6.54 | 7.13 |
| 67 | benzo[a]pyrene | 6.11 | -6.30 | 6.54 | 7.19 |
| 68 | benzo[ghi]perylene | 6.70 | -6.59 | 7.21 | 7.78 |
| 69 | dibenzo[a,h]anthracene | 6.70 | -6.41 | 7.18 | 7.72 |
| 70 | dibenzo[a,c]anthracene | 6.70 | -6.20 | 7.18 | 7.49 |
| 71 | indenol[1,2,3-cd] pyrene | 6.70 | -6.23 | 7.21 | 7.86 |
| 72 | nitrobenzene | 1.81 | -2.99 | 1.86 | 2.01 |
| 73 | 2-nitrotoluene | 2.36 | -2.78 | 2.44 | 2.41 |
| 74 | quinoline | 2.14 | -4.00 | 2.29 | 1.67 |
| 75 | diethyl phthalate | 2.65 | -5.49 | 2.85 | 1.77 |
| 76 | dibutyl phthalate | 4.61 | -4.83 | 4.92 | 3.87 |
| 77 | phenol | 1.51 | -4.75 | 1.62 | 1.96 |
| 78 | 2-methylphenol | 2.06 | -4.30 | 2.21 | 2.45 |
| 79 | 3-methylphenol | 2.06 | -4.71 | 2.21 | 2.34 |
| 80 | 4-methylphenol | 2.06 | -4.60 | 2.21 | 2.35 |
| 81 | 2-ethylphenol | 2.55 | -4.38 | 2.73 | 2.81 |
| 82 | 4-ethylphenol | 2.55 | -4.62 | 2.73 | 2.78 |
| 83 | 2,6-dimethylphenol | 2.61 | -3.93 | 2.80 | 2.47 |
| 84 | 2-n-propylphenol | 3.04 | -4.22 | 3.25 | 3.13 |
| 85 | 4-n-propylphenol | 3.04 | -4.49 | 3.25 | 2.92 |
| 86 | 4-isopropylphenol | 2.97 | -4.46 | 3.17 | 3.25 |
| 87 | 3,4,5-trimethylphenol | 3.15 | -5.01 | 3.37 | 2.66 |
| 88 | 4-n-butylphenol | 3.53 | -4.35 | 3.76 | 3.13 |
| 89 | 2-sec-butylphenol | 3.46 | -4.21 | 3.69 | 3.47 |
| 90 | 2-tert-butylphenol | 3.42 | -4.17 | 3.65 | 3.51 |
| 91 | 4-tert-butylphenol | 3.42 | -4.32 | 3.65 | 3.48 |
| 92 | 4-tert-amylphenol | 3.91 | -4.30 | 4.16 | 3.54 |
| 93 | 2-phenylphenol | 3.28 | -6.69 | 3.58 | 3.43 |
| 94 | 4-phenylphenol | 3.28 | -6.54 | 3.58 | 3.52 |
| 95 | bisphenol A | 3.64 | -10.56 | 4.17 | 3.92 |
| 96 | 2-chlorophenol | 2.16 | -3.63 | 2.33 | 2.76 |
| 97 | 3-chlorophenol | 2.16 | -4.87 | 2.33 | 2.78 |
| 98 | 4-chlorophenol | 2.16 | -5.13 | 2.33 | 2.73 |
| 99 | 4-chloro-3-methylphenol | 2.70 | -4.92 | 2.90 | 3.32 |
| 100 | 2,4-dichlorophenol | 2.80 | -3.67 | 3.02 | 3.57 |
| 101 | 2,6-dichlorophenol | 2.80 | -3.68 | 3.02 | 2.86 |
| 102 | 3,4-dichlorophenol | 2.80 | -4.75 | 3.02 | 3.76 |
| 103 | 2,4,5-trichlorophenol | 3.45 | -4.32 | 3.72 | 4.46 |
| 104 | 2,4,6-trichlorophenol | 3.45 | -4.10 | 3.72 | 3.80 |
| 105 | 3,4,5-trichlorophenol | 3.45 | -5.08 | 3.72 | 4.71 |
| 106 | 2,3,4,5-tetrachlorophenol | 4.09 | -4.26 | 4.41 | 4.76 |
| 107 | pentachlorophenol | 4.74 | -3.92 | 5.12 | 5.10 |
| 108 | 4-fluorophenol | 1.71 | -4.67 | 1.83 | 2.19 |
| 109 | 4-bromophenol | 2.40 | -5.31 | 2.60 | 2.40 |
| 110 | 4-iodophenol | 2.68 | -5.41 | 2.91 | 2.55 |
| 111 | 2-nitrophenol | 1.91 | -3.43 | 1.99 | 1.89 |
| 112 | 3-nitrophenol | 1.91 | -6.99 | 2.18 | 2.56 |
| 113 | 4-nitrophenol | 1.91 | -7.65 | 2.18 | 2.72 |
| 114 | 2,4-dinitrophenol | 1.73 | -5.56 | 1.93 | 2.67 |
| 115 | 2,6-dinitrophenol | 1.73 | -6.70 | 1.93 | 2.03 |
| 116 | 3,4-dinitrophenol | 1.73 | -9.02 | 2.12 | 3.17 |
| 117 | 2-sec-butyl-4,6-dinitrophenol | 3.67 | -5.32 | 3.98 | 3.73 |
| 118 | 4-cyanophenol | 1.61 | -7.38 | 1.84 | 2.11 |
| 119 | aniline | 1.08 | -4.16 | 1.14 | 1.63 |
| 120 | 3,4-dimethylaniline | 2.17 | -4.18 | 2.30 | 2.11 |
| 121 | 3-nitroaniline | 1.47 | -6.42 | 1.68 | 2.17 |
| 122 | N, N-dimethylaniline | 2.17 | -2.65 | 2.21 | 2.33 |
| 123 | estrone | 3.43 | -10.42 | 3.85 | 3.59 |
| 124 | -estradiol | 3.94 | -11.31 | 4.45 | 3.33 |
| 125 | estriol | 2.81 | -17.17 | 3.32 | 1.96 |
| 126 | progesterone | 3.67 | -10.87 | 3.99 | 3.28 |
| 127 | diazepam | 2.70 | -8.59 | 3.02 | 2.99 |
| 128 | lidocaine | 1.66 | -7.52 | 1.98 | 2.15 |
| 129 | diclofenac | 4.02 | -9.14 | 4.59 | 4.45 |
| 130 | ibuprofen | 3.79 | -5.68 | 4.10 | 3.80 |
| 131 | salicylic acid | 2.24 | -5.40 | 2.49 | 2.55 |

## **Table S9.** Showing the summary of results of existing models with the new developed models of current study for the estimation of logK_lw_ and logK_pw_.

| **logK_lw_** | | | | **logK_pw_** | | | **Reference** |
| --- | --- | --- | --- | --- | --- | --- | --- |
| Model | n | R^2^ | rmse | n | R^2^ | rmse |  |
| tp-LFER (current study) | 305 | 0.9709 | 0.375 | 131 | 0.952 | 0.413 |  |
| QSPR model | 302 | 0.955 | 0.468 |  |  |  | ^1^ |
| COSMOtherm | 304 | 0.963 | 0.498 | 207 |  | 1.01 | ^1–3^ |
| SPARC | 302 | 0.950 | 0.540 | 207 |  | 1.07 | ^1–3^ |
| ABSOLV | 304 | 0.932 | 0.610 |  |  |  | ^1–3^ |
| KOWWIN | 305 | 0.931 | 0.600 |  |  |  | ^1–3^ |
| COSMOmic |  |  |  | 207 |  | 0.79 | ^3^ |
| ASM | 247 | 0.977-0.988 | 0.20-0.29 | 131 | 0.976-0.979 | 0.275-0.294 | ^2,3^ |

## **Table S10**. Training set for logK_lw_

| **S. No** | **Chemicals** | **CAS No** | **SMILES** | **logK_lw_** | **logK_ow_** | **logK_aw_** |
| --- | --- | --- | --- | --- | --- | --- |
| 1 | 1,1,1,2-Tetrachloroethane | 630-20-6 | ClCC(Cl)(Cl)Cl | 2.85 | 2.84 | -0.97 |
| 2 | 1,1,1-Trichloroethane | 71-55-6 | CC(Cl)(Cl)Cl | 2.66 | 2.45 | -0.08 |
| 3 | 1,1,2,2-Tetrachloroethane | 79-34-5 | ClC(C(Cl)Cl)Cl | 2.46 | 2.58 | -1.74 |
| 4 | 1,1-Dichloroethane | 75-34-3 | CC(Cl)Cl | 1.74 | 1.81 | -0.77 |
| 5 | 1,2,4-Trifluorobenzene | 367-23-7 | Fc1ccc(c(c1)F)F | 2.67 | 2.31 | -0.38 |
| 6 | 1,2,4-Trimethylbenzene | 95-63-6 | Cc1ccc(c(c1)C)C | 3.43 | 3.55 | -0.72 |
| 7 | 1,2-Dibromoethane | 106-93-4 | BrCCBr | 1.67 | 1.9 | -1.91 |
| 8 | 1,2-Dichlorobenzene | 95-50-1 | Clc1ccccc1Cl | 3.71 | 3.36 | -0.94 |
| 9 | 1,2-Dichloroethane | 107-06-2 | ClCCCl | 1.6 | 1.68 | -1.25 |
| 10 | 1,2-Dichloropropane | 78-87-5 | ClCC(Cl)C | 1.99 | 1.96 | -0.92 |
| 11 | 1,2-Difluorobenzene | 367-11-3 | Fc1ccccc1F | 2.62 | 2.28 | -0.45 |
| 12 | 1,3,5-Trifluorobenzene | 372-38-3 | Fc1cc(F)cc(c1)F | 2.93 | 2.53 | 0.13 |
| 13 | 1,3,5-Trimethylbenzene | 108-67-8 | Cc1cc(C)cc(c1)C | 3.49 | 3.56 | -0.59 |
| 14 | 1,3-Dichlorobenzene | 541-73-1 | Clc1cccc(c1)Cl | 3.84 | 3.45 | -0.68 |
| 15 | 1,4-Difluorobenzene | 540-36-3 | Fc1ccc(cc1)F | 2.58 | 2.29 | -0.35 |
| 16 | 1-Bromo-2-chloroethane | 107-04-0 | ClCCBr | 2.07 | 1.96 | -1.34 |
| 17 | 1-Butanol | 71-36-3 | CCCCO | 0.02 | 0.91 | -3.36 |
| 18 | 1-Chloropentane | 543-59-9 | CCCCCCl | 3.48 | 3.07 | 0.11 |
| 19 | 1-Chloropropane | 540-54-5 | CCCCl | 2.22 | 1.95 | -0.12 |
| 20 | 1-Hexanol | 111-27-3 | CCCCCCO | 1.36 | 2.02 | -3.13 |
| 21 | 1-Propanol | 71-23-8 | CCCO | -0.48 | 0.32 | -3.45 |
| 22 | 2,2-Dimethylbutane | 75-83-2 | CCC(C)(C)C | 3.79 | 3.65 | 1.89 |
| 23 | 2,3,4-Trimethylpentane | 565-75-3 | CC(C(C)C)C(C)C | 5.05 | 4.82 | 2.07 |
| 24 | 2-Butoxyethanol | 111-76-2 | CCCCOCCO | -0.21 | 0.94 | -4.68 |
| 25 | 2-Chloropropane | 75-29-6 | CC(Cl)C | 2.03 | 1.86 | 0 |
| 26 | 2-Ethoxyethanol | 110-80-5 | OCCOCC | -1.27 | -0.17 | -4.93 |
| 27 | 2-Fluoropropane | 420-26-8 | CC(F)C | 1.38 | 1.33 | 0.33 |
| 28 | 2-Heptanone | 110-43-0 | CCCCCC(=O)C | 1.81 | 1.92 | -2.27 |
| 29 | 2-Hexanone | 591-78-6 | CCCCC(=O)C | 1.19 | 1.37 | -2.41 |
| 30 | 2-Isopropoxyethanol | 109-59-1 | OCCOC(C)C | -1.16 | 0.11 | -4.78 |
| 31 | 2-Methoxyethanol | 109-86-4 | COCCO | -1.67 | -0.68 | -5.18 |
| 32 | 2-Methyl-1-propanol | 78-83-1 | OCC(C)C | -0.06 | 0.87 | -3.21 |
| 33 | 2-Methyl-2-propanol | 75-65-0 | CC(O)(C)C | -0.73 | 0.4 | -3.17 |
| 34 | 2-Methylpentane | 107-83-5 | CCCC(C)C | 3.99 | 3.72 | 1.82 |
| 35 | 2-Nitropropane | 79-46-9 | CC(N(=O)=O)C | 0.61 | 0.74 | -2.3 |
| 36 | 2-Pentanone | 107-87-9 | CCCC(=O)C | 0.65 | 0.8 | -2.51 |
| 37 | 2-Propanol | 67-63-0 | CC(O)C | -0.82 | 0.02 | -3.44 |
| 38 | 3-Methyl-1-butanol | 137-32-6 | CCC(CO)C | 0.41 | 1.46 | -3.13 |
| 39 | 3-Methylhexane | 589-34-4 | CCCC(CC)C | 4.61 | 4.29 | 1.92 |
| 40 | 3-Methylpentane | 96-14-0 | CCC(CC)C | 4.07 | 3.75 | 1.78 |
| 41 | 3-Pentanone | 96-22-0 | CCC(=O)CC | 0.56 | 0.86 | -2.5 |
| 42 | 4-Methyl-2-pentanone | 108-10-1 | CC(CC(=O)C)C | 1.05 | 1.33 | -2.25 |
| 43 | Acetone | 67-64-1 | CC(=O)C | -0.32 | -0.3 | -2.81 |
| 44 | Benzene | 71-43-2 | c1ccccc1 | 2.12 | 2.05 | -0.68 |
| 45 | Bromochloromethane | 74-97-5 | ClCBr | 1.49 | 1.37 | -1.17 |
| 46 | 2-Butanone | 78-93-3 | CCC(=O)C | 0.18 | 0.23 | -2.69 |
| 47 | Butane | 106-97-8 | CCCC | 3.03 | 2.65 | 1.53 |
| 48 | Butyl acetate | 123-86-4 | CCCCOC(=O)C | 1.59 | 1.86 | -1.83 |
| 49 | Carbon tetrachloride | 56-23-5 | ClC(Cl)(Cl)Cl | 3.18 | 2.8 | 0.33 |
| 50 | 1,2-Dichlorotetrafluoroethane | 76-14-2 | FC(C(Cl)(F)F)(Cl)F | 3 | 2.58 | 1.6 |
| 51 | 1,1,2,2,3,3,4,4-Octafluorobutane | 377-36-6 | FC(C(C(C(F)F)(F)F)(F)F)F | 2.5 | 2.27 | 0.52 |
| 52 | 1,1,2,2,3,3-Hexafluoropropane | 680-00-2 | FC(C(C(F)F)(F)F)F | 1.49 | 1.48 | 0.45 |
| 53 | 1,1,2,2-Tetrafluoroethane | 359-35-3 | FC(C(F)F)F | 0.92 | 0.85 | 0.13 |
| 54 | 1,1-Difluoroethane | 75-37-6 | CC(F)F | 0.91 | 0.7 | -0.06 |
| 55 | Halothane | 151-67-7 | ClC(C(F)(F)F)Br | 2.28 | 2.4 | -0.05 |
| 56 | 1,3-Difluoropropane | 462-39-5 | FCCCF | 0.66 | 0.76 | -1.21 |
| 57 | Enflurane | 13838-16-9 | FC(OC(C(Cl)F)(F)F)F | 2.03 | 2.03 | 0.03 |
| 58 | Isoflurane | 26675-46-7 | FC(OC(C(F)(F)F)Cl)F | 1.99 | 1.88 | -0.01 |
| 59 | Desflurane | 57041-67-5 | FC(OC(C(F)(F)F)F)F | 1.88 | 1.76 | 0.73 |
| 60 | Sevoflurane | 28523-86-6 | FCOC(C(F)(F)F)C(F)(F)F | 1.87 | 1.91 | 0.4 |
| 61 | Chlorobenzene | 108-90-7 | Clc1ccccc1 | 2.73 | 2.78 | -0.71 |
| 62 | Chlorodibromomethane | 124-48-1 | ClC(Br)Br | 2.12 | 2.18 | -1.49 |
| 63 | Chloroethane | 75-00-3 | CCCl | 1.58 | 1.39 | -0.22 |
| 64 | Chloroform | 67-66-3 | ClC(Cl)Cl | 2.02 | 2.11 | -0.69 |
| 65 | cis-1,2-Dichloroethene | 156-59-2 | Cl/C=C\Cl | 1.59 | 1.76 | -0.98 |
| 66 | Cycloheptane | 291-64-5 | C1CCCCCC1 | 4.82 | 4.17 | 1.12 |
| 67 | Cyclohexane | 110-82-7 | C1CCCCC1 | 4.05 | 3.51 | 1.12 |
| 68 | Cyclopentane | 287-92-3 | C1CCCC1 | 3.5 | 2.96 | 0.99 |
| 69 | Dibromethane | 74-95-3 | BrCBr | 1.76 | 1.72 | -1.51 |
| 70 | Dichloromethane | 75-09-2 | ClCCl | 1.28 | 1.41 | -0.91 |
| 71 | Difluoromethane | 75-10-5 | FCF | 0.53 | 0.17 | -0.18 |
| 72 | Divinyl ether | 109-93-3 | C=COC=C | 1.69 | 1.65 | -0.05 |
| 73 | Ethane | 74-84-0 | CC | 1.73 | 1.49 | 1.35 |
| 74 | Ethanol | 64-17-5 | CCO | -1.14 | -0.25 | -3.54 |
| 75 | Ethene | 74-85-1 | C=C | 0.84 | 0.92 | 0.79 |
| 76 | Ethyl acetate | 141-78-6 | CCOC(=O)C | 0.46 | 0.71 | -2.09 |
| 77 | Ethyl tert-butyl ether | 637-92-3 | CCOC(C)(C)C | 1.37 | 1.72 | -1.16 |
| 78 | Ethyl tert-pentyl ether | 919-94-8 | CCOC(CC)(C)C | 1.93 | 2.19 | -1.33 |
| 79 | Ethylbenzene | 100-41-4 | CCc1ccccc1 | 3.14 | 3.13 | -0.46 |
| 80 | Fluorobenzene | 462-06-6 | Fc1ccccc1 | 2.41 | 2.16 | -0.54 |
| 81 | Fluoroethane | 353-36-6 | CCF | 0.62 | 0.73 | 0.14 |
| 82 | Fluorochloromethane | 593-70-4 | FCCl | 0.77 | 0.69 | -0.6 |
| 83 | Hexafluorobenzene | 392-56-3 | Fc1c(F)c(F)c(c(c1F)F)F | 2.45 | 2.51 | 0.35 |
| 84 | Hexane | 110-54-3 | CCCCCC | 4.11 | 3.79 | 1.74 |
| 85 | Isobutyl acetate | 110-19-0 | CC(COC(=O)C)C | 1.6 | 1.75 | -1.77 |
| 86 | Isopropyl acetate | 108-21-4 | CC(OC(=O)C)C | 1.02 | 1.15 | -1.84 |
| 87 | Isopropylbenzene | 98-82-8 | CC(c1ccccc1)C | 3.52 | 3.6 | -0.26 |
| 88 | Methoxyflurane | 76-38-0 | COC(C(Cl)Cl)(F)F | 2.11 | 2.52 | -0.82 |
| 89 | Methane | 74-82-8 | C | 0.75 | 0.8 | 1.38 |
| 90 | Methyl acetate | 79-20-9 | COC(=O)C | -0.02 | 0.17 | -2.3 |
| 91 | Methyl chloride | 74-87-3 | CCl | 0.8 | 0.85 | -0.3 |
| 92 | Methylcyclopentane | 96-37-7 | CC1CCCC1 | 4 | 3.45 | 1.19 |
| 93 | m-Xylene | 108-38-3 | Cc1cccc(c1)C | 3.16 | 3.11 | -0.56 |
| 94 | o-Xylene | 95-47-6 | Cc1ccccc1C | 3.12 | 3.1 | -0.69 |
| 95 | Pentachloroethane | 76-01-7 | ClC(C(Cl)(Cl)Cl)Cl | 2.93 | 3.42 | -1.18 |
| 96 | Pentafluorobenzene | 363-72-4 | Fc1cc(F)c(c(c1F)F)F | 2.31 | 2.36 | -0.1 |
| 97 | Pentane | 109-66-0 | CCCCC | 3.49 | 3.23 | 1.63 |
| 98 | p-Methylstyrene | 622-97-9 | C=Cc1ccc(cc1)C | 3.21 | 3.34 | -0.95 |
| 99 | Propane | 74-98-6 | CCC | 2.35 | 2.07 | 1.44 |
| 100 | Propyl acetate | 109-60-4 | CCCOC(=O)C | 1.02 | 1.29 | -1.93 |
| 101 | Propylbenzene | 103-65-1 | CCCc1ccccc1 | 3.65 | 3.68 | -0.3 |
| 102 | Tetrachloroethene | 127-18-4 | ClC(=C(Cl)Cl)Cl | 3.57 | 3.28 | 0.09 |
| 103 | tridecane | 629-50-5 | CCCCCCCCCCCCC | 8.16 | 7.68 | 2.56 |
| 104 | triethylamine | 121-44-8 | CCN(CC)CC | 1.04 | 1.25 | -2.34 |
| 105 | alpha-pinene | 80-56-8 | CC1=CCC2CC1C2(C)C | 4.58 | 4.61 | 0.85 |
| 106 | 1,2-dimethoxyethane | 110-71-4 | COCCOC | -0.33 | 0.09 | -3.37 |
| 107 | 1,4-dioxane | 123-91-1 | O1CCOCC1 | -0.24 | -0.04 | -3.73 |
| 108 | 1-heptanol | 111-70-6 | CCCCCCCO | 1.84 | 2.57 | -3.01 |
| 109 | 3-methylpyridine | 108-99-6 | Cc1cccnc1 | 0.84 | 0.86 | -3.38 |
| 110 | 4-methylpyridine | 108-89-4 | Cc1ccncc1 | 0.82 | 0.82 | -3.45 |
| 111 | benzyl alcohol | 100-51-6 | OCc1ccccc1 | 0.12 | 1.14 | -4.84 |
| 112 | bromobenzene | 108-86-1 | Brc1ccccc1 | 3.27 | 2.89 | -1.03 |
| 113 | butyl propanoate | 590-01-2 | CCCCOC(=O)CC | 2.35 | 2.4 | -1.72 |
| 114 | butylbenzene | 104-51-8 | CCCCc1ccccc1 | 4.13 | 4.22 | -0.2 |
| 115 | 1,1-difluoro-2-chloroethene | 359-10-4 | ClC=C(F)F | 1.63 | 1.45 | 0.4 |
| 116 | bis-(2,2,2-trifluoroethyl)ether | 333-36-8 | FC(COCC(F)(F)F)(F)F | 1.92 | 1.69 | -0.09 |
| 117 | cyclopentanone | 120-92-3 | O=C1CCCC1 | 0.39 | 0.45 | -3.43 |
| 118 | difluorochloromethane | 75-45-6 | FC(Cl)F | 0.72 | 0.9 | 0.12 |
| 119 | diisopropyl ether | 108-20-3 | CC(OC(C)C)C | 1.29 | 1.72 | -1.08 |
| 120 | dimethylacetamide | 127-19-5 | CC(=O)N(C)C | -1.25 | -0.76 | -5.69 |
| 121 | dimethylformamide | 68-12-2 | O=CN(C)C | -1.57 | -1.1 | -5.55 |
| 122 | di-n-butyl ether | 142-96-1 | CCCCOCCCC | 3.14 | 3.24 | -0.7 |
| 123 | ethyl formate | 109-94-4 | CCOC=O | 0.16 | 0.35 | -1.97 |
| 124 | ethyl propanoate | 105-37-3 | CCOC(=O)CC | 1.13 | 1.32 | -1.89 |
| 125 | fluorotrichloromethane | 75-69-4 | FC(Cl)(Cl)Cl | 2.28 | 2.13 | 0.43 |
| 126 | methyl formate | 107-31-3 | COC=O | -0.48 | -0.26 | -2.1 |
| 127 | methylcyclohexane | 108-87-2 | CC1CCCCC1 | 4.54 | 4 | 1.3 |
| 128 | nitroethane | 79-24-3 | CCN(=O)=O | 0.45 | 0.26 | -2.78 |
| 129 | nitromethane | 75-52-5 | CN(=O)=O | -0.02 | -0.28 | -2.98 |
| 130 | N,N-dimethylaniline | 121-69-7 | CN(c1ccccc1)C | 2.29 | 2.37 | -2.65 |
| 131 | pentadecane | 629-62-9 | CCCCCCCCCCCCCCC | 9.31 | 8.8 | 2.8 |
| 132 | piperidine | 110-89-4 | C1CCCNC1 | 1.01 | 0.67 | -3.87 |
| 133 | propyl bromide | 106-94-5 | CCCBr | 2.47 | 2.19 | -0.28 |
| 134 | propyl formate | 110-74-7 | CCCOC=O | 0.87 | 0.99 | -1.83 |
| 135 | pyridine | 110-86-1 | c1cccnc1 | 0.15 | 0.29 | -3.41 |
| 136 | tetradecane | 629-59-4 | CCCCCCCCCCCCCC | 8.74 | 8.24 | 2.68 |
| 137 | tetrahydrofuran | 109-99-9 | C1CCCO1 | 0.62 | 0.58 | -2.5 |
| 138 | halopropane | 679-84-5 | BrCC(C(F)F)(F)F | 2.91 | 2.65 | 0.28 |
| 139 | Cyclooctane | 292-64-8 | C1CCCCCCC1 | 5.34 | 4.78 | 1.18 |
| 140 | Octan-1-ol | 111-87-5 | CCCCCCCCO | 2.46 | 3.13 | -2.89 |
| 141 | Nonan-1-ol | 143-08-8 | CCCCCCCCCO | 3.01 | 3.69 | -2.77 |
| 142 | Hexanal | 66-25-1 | CCCCCC=O | 1.92 | 1.65 | -2.09 |
| 143 | Heptanal | 111-71-7 | CCCCCCC=O | 2.46 | 2.21 | -1.97 |
| 144 | Nonanal / n-Nonyl Aldehyde | 124-19-6 | CCCCCCCCC=O | 3.42 | 3.31 | -1.73 |
| 145 | 1-Chloroheptane | 629-06-1 | CCCCCCCCl | 4.37 | 4.21 | 0.32 |
| 146 | 1-Hexene | 592-41-6 | CCCCC=C | 3.51 | 3.29 | 1.17 |
| 147 | 1-Heptene | 592-76-7 | CCCCCC=C | 4.04 | 3.84 | 1.3 |
| 148 | 1-Octene | 111-66-0 | CCCCCCC=C | 4.6 | 4.39 | 1.41 |
| 149 | 1-Nonene | 124-11-8 | CCCCCCCC=C | 5.15 | 4.95 | 1.53 |
| 150 | 1-Decene | 872-05-9 | CCCCCCCCC=C | 5.64 | 5.49 | 1.67 |
| 151 | 1,2,4-Trichlorobenzene | 120-82-1 | Clc1ccc(c(c1)Cl)Cl | 4.17 | 4.07 | -0.84 |
| 152 | Dipentyl ether | 693-65-2 | CCCCCOCCCCC | 4.16 | 4.330133 | -0.43944 |
| 153 | 2-octanone | 111-13-7 | CCCCCCC(=O)C | 2.31 | 2.47 | -2.15 |
| 154 | 2-nonanone | 821-55-6 | CCCCCCCC(=O)C | 2.79 | 3.01 | -2.02 |
| 155 | 1-Nitrobutane | 627-05-4 | CCCCN(=O)=O | 1.53 | 1.51 | -2.27 |
| 156 | 1-Nitrohexane | 646-14-0 | CCCCCCN(=O)=O | 2.58 | 2.617842 | -2.03157 |
| 157 | 4-Ethylpyridine | 536-75-4 | CCc1ccncc1 | 1.14 | 1.33 | -3.38 |
| 158 | 1-Chloro-4-nitrobenzene | 100-00-5 | Clc1ccc(cc1)N(=O)=O | 2.38 | 2.53 | -2.94 |
| 159 | Nitrobenzene | 98-95-3 | O=N(=O)c1ccccc1 | 1.92 | 1.91 | -2.99 |
| 160 | 2-Nitrotoluene | 88-72-2 | O=N(=O)c1ccccc1C | 2.4 | 2.39 | -2.78 |
| 161 | 2,6-Dinitrotoluene | 606-20-2 | Cc1c(cccc1N(=O)=O)N(=O)=O | 1.94 | 2.157397 | -4.69323 |
| 162 | 1,4-Dimethoxybenzene | 150-78-7 | COc1ccc(cc1)OC | 2.12 | 2.062516 | -3.49134 |
| 163 | 4-Chlorophenol | 106-48-9 | Oc1ccc(cc1)Cl | 1.51 | 2.19 | -5.13 |
| 164 | Ethyl benzoate | 93-89-0 | CCOC(=O)c1ccccc1 | 2.59 | 2.66 | -2.75 |
| 165 | Indole | 120-72-9 | c1ccc2c(c1)[nH]cc2 | 1.99 | 2.486724 | -4.55008 |
| 166 | 2-Ethyl-1-hexanol | 104-76-7 | CCCCC(CO)CC | 2.04 | 3.093435 | -2.73967 |
| 167 | 3-Ethyl-3-hexanol | 597-76-2 | CCCC(CC)(CC)O | 1.7 | 2.76 | -2.85 |
| 168 | 4-Ethyl-3-hexanol | 19780-44-0 | CCC(C(CC)O)CC | 1.92 | 2.72 | -2.85 |
| 169 | 3-Ethyl-3-pentanol | 597-49-9 | CCC(CC)(CC)O | 1.13 | 2.083716 | -3.18606 |
| 170 | 2,4-Dinitrotoluene | 121-14-2 | O=N(=O)c1ccc(c(c1)N(=O)=O)C | 2.34 | 1.42 | -5.88 |
| 171 | Hexachloroethane | 67-72-1 | ClC(C(Cl)(Cl)Cl)(Cl)Cl | 3.71 | 4.12 | -0.21 |
| 172 | hexadecane | 544-76-3 | CCCCCCCCCCCCCCCC | 9.88 | 9.35 | 2.91 |
| 173 | isopropyl bromide | 75-26-3 | CC(Br)C | 2.28 | 2.09 | -0.16 |
| 174 | beta-pinene | 127-91-3 | C=C1CCC2CC1C2(C)C | 4.11 | 4.27 | 0.27 |
| 175 | vinyl chloride | 75-01-4 | ClC=C | 1.58 | 1.37 | 0.08 |
| 176 | formic acid | 64-18-6 | OC=O | -1.69 | -0.55 | -5.27 |
| 177 | 3-carene | 13466-78-9 | CC1=CCC2C(C1)C2(C)C | 4.71 | 4.71 | 0.62 |
| 178 | vinyl bromide | 593-60-2 | BrC=C | 1.34 | 1.45 | -0.34 |
| 179 | 4-Chloroaniline | 106-47-8 | Nc1ccc(cc1)Cl | 1.62 | 1.99 | -4.31 |
| 180 | allyl chloride | 107-05-1 | ClCC=C | 1.84 | 1.76 | -0.27 |
| 181 | Styrene | 100-42-5 | C=Cc1ccccc1 | 2.68 | 2.83 | -0.95 |
| 182 | Octane | 111-65-9 | CCCCCCCC | 5.27 | 4.9 | 1.98 |
| 183 | 3-Chlorophenol | 108-43-0 | Oc1cccc(c1)Cl | 1.66 | 2.42 | -4.87 |
| 184 | Benzyl acetate | 140-11-4 | CC(=O)OCc1ccccc1 | 1.7 | 1.690335 | -4.08968 |
| 185 | 4-bromophenol | 106-41-2 | Oc1ccc(cc1)Br | 1.61 | 2.38 | -5.31 |
| 186 | 4-Iodoaniline | 540-37-4 | Nc1ccc(cc1)I | 2.19 | 2.137554 | -5.13918 |
| 187 | N,N-Diethylaniline | 91-66-7 | CCN(c1ccccc1)CC | 3.17 | 3.396228 | -2.08197 |
| 188 | 4-iodophenol | 540-38-5 | Oc1ccc(cc1)I | 2.29 | 2.66 | -5.41 |
| 189 | 1,3-Dinitrobenzene | 99-65-0 | O=N(=O)c1cccc(c1)N(=O)=O | 1.42 | 1.57 | -5.13 |
| 190 | Anthracene | 120-12-7 | c1ccc2c(c1)cc1c(c2)cccc1 | 4.83 | 4.24 | -3.47 |
| 191 | Pyrene | 129-00-0 | c1cc2ccc3c4c2c(c1)ccc4ccc3 | 5.26 | 4.58 | -4.51 |
| 192 | Acenaphthene | 83-32-9 | c1cc2cccc3c2c(c1)CC3 | 3.97 | 3.92 | -2.55 |
| 193 | phenol | 108-95-2 | Oc1ccccc1 | -0.16 | 1.43 | -4.75 |
| 194 | carbon disulfide | 75-15-0 | S=C=S | 2.46 | 2.07 | 0.02 |
| 195 | acetylene | 74-86-2 | C#C | -0.63 | 0.25 | -0.32 |
| 196 | methanal | 50-00-0 | C=O | -0.41 | -0.83 | -2.14 |
| 197 | 1-Propanethiol | 107-03-9 | CCCS | 1.93 | 1.85 | -0.82 |
| 198 | 1-Butanethiol | 109-79-5 | CCCCS | 2.53 | 2.37 | -0.67 |
| 199 | 1-Hexanethiol | 111-31-9 | CCCCCCS | 4.02 | 3.49 | -0.44 |
| 200 | 2-Hexanol | 626-93-7 | CCCCC(O)C | 0.91 | 1.72 | -3.11 |
| 201 | 3-Hexanol | 623-37-0 | CCCC(CC)O | 0.48 | 1.72 | -3.12 |
| 202 | 4-Heptanol | 589-55-9 | CCCC(CCC)O | 1.67 | 2.28 | -3 |
| 203 | 4-Octanol | 589-62-8 | CCCCC(CCC)O | 2.42 | 2.81 | -2.86 |
| 204 | 2,2,2-Trifluoroethanol | 75-89-8 | OCC(F)(F)F | -0.55 | 0.27 | -3.27 |
| 205 | 1,1,1-Trifluoro-2-propanol | 374-01-6 | CC(C(F)(F)F)O | -0.05 | 0.76 | -2.8 |
| 206 | 2,2,3,3-Tetrafluoro-1-propanol | 76-37-9 | OCC(C(F)F)(F)F | -0.1 | 1.39 | -3.27 |
| 207 | 2,2,3,3,3-Pentafluoro-1-propanol | 422-05-9 | OCC(C(F)(F)F)(F)F | 0.34 | 1.5 | -2.23 |
| 208 | 1,1,1,3,3,3-Hexafluoro-2-propanol | 920-66-1 | OC(C(F)(F)F)C(F)(F)F | 0.76 | 1.36 | -2.75 |
| 209 | 1,1,1,3,3,3-Hexafluoro-2-methyl-2-propanol | 1515-14-6 | CC(C(F)(F)F)(C(F)(F)F)O | 1.1 | 2.16 | -1.63 |
| 210 | 2,2,3,4,4,4-Hexafluoro-1-butanol | 382-31-0 | OCC(C(C(F)(F)F)F)(F)F | 0.66 | 1.99 | -2.8 |
| 211 | 2-Butanol | 78-92-2 | CCC(O)C | -0.28 | 0.61 | -3.35 |
| 212 | Triethyl phosphate | 78-40-0 | CCOP(=O)(OCC)OCC | 0.22 | 0.67 | -5.37 |
| 213 | PCB 28 | 7012-37-5 | Clc1ccc(cc1)c1ccc(cc1Cl)Cl | 5.83 | 5.42 | -2.37 |
| 214 | PCB 31 | 16606-02-3 | Clc1ccc(cc1)c1cc(Cl)ccc1Cl | 5.83 | 5.4 | -2.35 |
| 215 | PCB 44 | 41464-39-5 | Clc1ccc(c(c1)c1cccc(c1Cl)Cl)Cl | 6.01 | 5.68 | -2.57 |
| 216 | PCB 49 | 41464-40-8 | Clc1ccc(c(c1)Cl)c1cc(Cl)ccc1Cl | 6.03 | 5.63 | -2.51 |
| 217 | PCB 99 | 38380-01-7 | Clc1ccc(c(c1)Cl)c1cc(Cl)c(cc1Cl)Cl | 6.52 | 6.12 | -2.72 |
| 218 | PCB 101 | 37680-73-2 | Clc1ccc(c(c1)c1cc(Cl)c(cc1Cl)Cl)Cl | 6.52 | 6.1 | -2.7 |
| 219 | PCB 105 | 32598-14-4 | Clc1ccc(cc1Cl)c1ccc(c(c1Cl)Cl)Cl | 6.82 | 6.51 | -2.9 |
| 220 | PCB 110 | 38380-03-9 | Clc1cc(ccc1Cl)c1c(Cl)ccc(c1Cl)Cl | 6.53 | 6.23 | -2.84 |
| 221 | PCB 118 | 31508-00-6 | Clc1cc(Cl)c(cc1c1ccc(c(c1)Cl)Cl)Cl | 6.81 | 6.43 | -2.81 |
| 222 | PCB 128 | 38380-07-3 | Clc1c(ccc(c1Cl)Cl)c1ccc(c(c1Cl)Cl)Cl | 6.97 | 6.75 | -3.08 |
| 223 | PCB 138 | 35065-28-2 | Clc1cc(Cl)c(cc1c1ccc(c(c1Cl)Cl)Cl)Cl | 7 | 6.67 | -2.99 |
| 224 | PCB 149 | 38380-04-0 | Clc1cc(Cl)c(cc1c1c(Cl)ccc(c1Cl)Cl)Cl | 6.83 | 6.49 | -2.79 |
| 225 | PCB 151 | 52663-63-5 | Clc1ccc(c(c1)c1c(Cl)c(Cl)cc(c1Cl)Cl)Cl | 6.77 | 6.4 | -2.69 |
| 226 | PCB 153 | 35065-27-1 | Clc1cc(Cl)c(cc1c1cc(Cl)c(cc1Cl)Cl)Cl | 7.03 | 6.59 | -2.9 |
| 227 | PCB 156 | 38380-08-4 | Clc1ccc(cc1Cl)c1cc(Cl)c(c(c1Cl)Cl)Cl | 7.34 | 6.95 | -3.06 |
| 228 | PCB 170 | 35065-30-6 | Clc1c(Cl)ccc(c1Cl)c1cc(Cl)c(c(c1Cl)Cl)Cl | 7.45 | 7.2 | -3.23 |
| 229 | PCB 187 | 52663-68-0 | Clc1cc(Cl)c(cc1c1c(Cl)c(Cl)cc(c1Cl)Cl)Cl | 7.27 | 6.89 | -2.89 |
| 230 | PCB 188 | 74487-85-7 | Clc1cc(Cl)c(c(c1)Cl)c1c(Cl)c(Cl)cc(c1Cl)Cl | 7.03 | 6.7 | -2.68 |
| 231 | PCB 194 | 35694-08-7 | Clc1cc(c(c(c1Cl)Cl)Cl)c1cc(Cl)c(c(c1Cl)Cl)Cl | 8.01 | 7.67 | -3.34 |
| 232 | PCB 209 | 2051-24-3 | Clc1c(c2c(Cl)c(Cl)c(c(c2Cl)Cl)Cl)c(Cl)c(c(c1Cl)Cl)Cl | 8.26 | 8.26 | -3.31 |
| 233 | Naphthalene | 91-20-3 | c1ccc2c(c1)cccc2 | 3.61 | 3.19 | -2 |
| 234 | Benz[a]anthracene | 56-55-3 | c1ccc2c(c1)cc1c(c2)ccc2c1cccc2 | 6.26 | 5.624024 | -4.82611 |
| 235 | Chrysene | 218-01-9 | c1ccc2c(c1)c1ccc3c(c1cc2)cccc3 | 6.25 | 5.6 | -4.91 |
| 236 | Benzo[a]pyrene | 50-32-8 | c1ccc2c(c1)c1ccc3c4c1c(c2)ccc4ccc3 | 6.79 | 5.78 | -6.3 |
| 237 | Benzo[k]fluoranthene | 207-08-9 | c1ccc2c(c1)cc1c(c2)c2c3c1cccc3ccc2 | 6.84 | 6.21 | -5.56 |
| 238 | Teflurane | 124-72-1 | FC(C(F)(F)F)Br | 1.8 | 2.13 | 0.39 |
| 239 | 2-[Chloro(fluoro)methoxy]-1,1,1,2-tetrafluoroethane | 56885-28-0 | FC(OC(C(F)(F)F)F)Cl | 2.15 | 1.99 | 0.17 |
| 240 | 1,1,1,2,3,4,4,4-Octafluorobutane | 75995-72-1 | FC(C(F)(F)F)C(C(F)(F)F)F | 2.44 | 2.29 | 1 |
| 241 | 1,1,1,2,2,3,3,4,4-nonafluorobutane | 375-17-7 | FC(C(C(C(F)(F)F)(F)F)(F)F)F | 2.82 | 2.9 | 2.63 |
| 242 | Sulfur hexafluoride | 2551-62-4 | FS(F)(F)(F)(F)F | 1.85 | 1.69 | 2.25 |
| 243 | 2-(Chlorodifluoromethoxy)-1,1,1-trifluoroethane | 33018-78-9 | FC(OCC(F)(F)F)(Cl)F | 2.73 | 2.58 | 1.39 |
| 244 | 3,4-Dichlorophenol | 95-77-2 | Oc1ccc(c(c1)Cl)Cl | 2.42 | 3.329159 | -4.63946 |
| 245 | 1-Nitronaphthalene | 86-57-7 | O=N(=O)c1cccc2c1cccc2 | 3.45 | 3.17 | -4.29 |

## **Table S11**. Validation set for logK_lw_

| **S. No** | **Chemicals** | **CAS No** | **SMILES** | **logK_lw_** | **logK_ow_** | **logK_aw_** |
| --- | --- | --- | --- | --- | --- | --- |
| 1 | 1,1,2-Trichloroethane | 79-00-5 | ClCC(Cl)Cl | 2.09 | 2.32 | -1.33 |
| 2 | 1,1-Dichloro-1-fluoroethane | 1717-00-6 | CC(Cl)(Cl)F | 1.34 | 1.96 | 0.16 |
| 3 | 1,1-Dichloroethene | 75-35-4 | ClC(=C)Cl | 2.19 | 2.02 | 0.14 |
| 4 | 1,2,3-Trimethylbenzene | 526-73-8 | Cc1c(C)cccc1C | 3.32 | 3.53 | -0.88 |
| 5 | 1-Chlorobutane | 109-69-3 | CCCCCl | 2.78 | 2.52 | -0.01 |
| 6 | 1-Methoxy-2-propanol | 107-98-2 | COCC(O)C | -1.53 | -0.26 | -4.92 |
| 7 | 1-Nitropropane | 108-03-2 | CCCN(=O)=O | 0.97 | 0.88 | -2.48 |
| 8 | 1-Pentanol | 71-41-0 | CCCCCO | 0.54 | 1.46 | -3.24 |
| 9 | 2,2,4-Trimethylpentane | 540-84-1 | CC(CC(C)(C)C)C | 4.64 | 4.65 | 2.25 |
| 10 | 2,2-Dichloro-1,1,1-trifluoroethane | 306-83-2 | ClC(C(F)(F)F)Cl | 1.81 | 2.15 | -0.13 |
| 11 | Allylbenzene | 300-57-2 | C=CCc1ccccc1 | 2.96 | 3.16 | -0.91 |
| 12 | 1,1,1,2-Tetrafluoroethane | 811-97-2 | FCC(F)(F)F | 1.03 | 1.2 | 0.41 |
| 13 | Fluroxene | 406-90-6 | C=COCC(F)(F)F | 1.26 | 1.46 | -0.22 |
| 14 | Carbon tetrafluoride | 56-23-5 | ClC(Cl)(Cl)Cl | 1.12 | 2.8 | 0.33 |
| 15 | Cyclopropane | 75-19-4 | C1CC1 | 1.73 | 1.6 | 0.56 |
| 16 | Decane | 124-18-5 | CCCCCCCCCC | 6.39 | 6.01 | 2.21 |
| 17 | Diethyl ether | 60-29-7 | CCOCC | 0.79 | 1.08 | -1.19 |
| 18 | Heptane | 142-82-5 | CCCCCCC | 4.71 | 4.34 | 1.86 |
| 19 | Isopentyl acetate | 123-92-2 | CC(CCOC(=O)C)C | 2.11 | 2.34 | -1.69 |
| 20 | Methanol | 67-56-1 | CO | -1.95 | -0.82 | -3.87 |
| 21 | Methylpentafluorobenzene | 771-56-2 | Fc1c(C)c(F)c(c(c1F)F)F | 3.27 | 3.15 | 0.17 |
| 22 | m-Methylstyrene | 100-80-1 | C=Cc1cccc(c1)C | 3.23 | 3.33 | -0.94 |
| 23 | Nonane | 111-84-2 | CCCCCCCCC | 5.82 | 5.46 | 2.09 |
| 24 | p-Xylene | 106-42-3 | Cc1ccc(cc1)C | 3.16 | 3.11 | -0.56 |
| 25 | Pentyl acetate | 628-63-7 | CCCCCOC(=O)C | 2.11 | 2.41 | -1.71 |
| 26 | Toluene | 108-88-3 | Cc1ccccc1 | 2.67 | 2.62 | -0.58 |
| 27 | trans-1,2-Dichloroethene | 156-60-5 | Cl/C=C/Cl | 2.06 | 1.98 | -0.42 |
| 28 | Trichloroethene | 79-01-6 | ClC=C(Cl)Cl | 2.8 | 2.71 | -0.23 |
| 29 | undecane | 1120-21-4 | CCCCCCCCCCC | 7.03 | 6.57 | 2.33 |
| 30 | 2-methylpyridine | 109-06-8 | Cc1ccccn1 | 0.61 | 0.75 | -3.3 |
| 31 | butyl formate | 592-84-7 | CCCCOC=O | 1.46 | 1.55 | -1.72 |
| 32 | 1-chloro-2,2,2-trifluoroethane | 75-88-7 | ClCC(F)(F)F | 1.34 | 1.62 | 0.09 |
| 33 | cyclohexene | 110-83-8 | C1CCC=CC1 | 3.33 | 2.95 | 0.29 |
| 34 | dimethoxymethane | 109-87-5 | COCOC | 0.18 | 0.28 | -2.15 |
| 35 | dimethyl ether | 115-10-6 | COC | 0.13 | 0.18 | -1.44 |
| 36 | dodecane | 112-40-3 | CCCCCCCCCCCC | 7.59 | 7.13 | 2.44 |
| 37 | iodoethane | 75-03-6 | CCI | 1.85 | 1.93 | -0.55 |
| 38 | Octanal | 124-13-0 | CCCCCCCC=O | 3.01 | 2.76 | -1.85 |
| 39 | 1-Chlorooctane | 111-85-3 | CCCCCCCCCl | 4.95 | 4.76 | 0.53 |
| 40 | Di-n-propyl ether | 111-43-3 | CCCOCCC | 2.06 | 2.16 | -0.77 |
| 41 | 4-Nitroanisole | 100-17-4 | COc1ccc(cc1)N(=O)=O | 2.32 | 1.77 | -4.67 |
| 42 | 1-fluropropane | 460-13-9 | CCCF | 0.98 | 1.2 | 0.1 |
| 43 | Biphenyl | 92-52-4 | c1ccc(cc1)c1ccccc1 | 4.14 | 3.82 | -2.24 |
| 44 | limonene | 138-86-3 | CC1=CCC(CC1)C(=C)C | 4.17 | 4.31367 | -0.04839 |
| 45 | Fluoromethane | 593-53-3 | CF | 0 | 0.2 | 0.08 |
| 46 | Tricyclo[5.2.1.0(2,6)]decane | 2825-83-4 | C1CC2C(C1)C1CC2CC1 | 4.62 | 4.45 | 0.08 |
| 47 | Methyl tert-butyl ether | 1634-04-4 | COC(C)(C)C | 0.89 | 1.16 | -1.49 |
| 48 | dimethyl sulfoxide | 67-68-5 | CS(=O)C | -2.66 | -2.19 | -7.4 |
| 49 | 1-Naphthol | 90-15-3 | Oc1cccc2c1cccc2 | 2.19 | 2.87 | -5.65 |
| 50 | 4-n-Propylphenol | 645-56-7 | CCCc1ccc(cc1)O | 2.19 | 2.84 | -4.49 |
| 51 | Phenanthrene | 85-01-8 | c1ccc2c(c1)c1ccccc1cc2 | 4.8 | 4.41 | -3.3 |
| 52 | Fluoranthene | 206-44-0 | c1ccc2c(c1)c1cccc3c1c2ccc3 | 5.18 | 4.94 | -3.98 |
| 53 | Fluorene | 86-73-7 | c1ccc2c(c1)Cc1c2cccc1 | 4.39 | 4.23 | -2.69 |
| 54 | 1-Pentanethiol | 110-66-7 | CCCCCS | 3.28 | 2.92 | -0.57 |
| 55 | 2-Pentanol | 6032-29-7 | CCCC(O)C | 0.3 | 1.16 | -3.23 |
| 56 | 2-Heptanol | 543-49-7 | CCCCCC(O)C | 1.52 | 2.27 | -2.99 |
| 57 | 2,2,3,3,4,4,4-Heptafluoro-1-butanol | 375-01-9 | OCC(C(C(F)(F)F)(F)F)(F)F | 1.17 | 2.48 | -1.6 |
| 58 | 1,2-Dihydroxybenzene / Catechol | 120-80-9 | Oc1ccccc1O | 0.79 | 0.807258 | -7.28661 |
| 59 | PCB 52 | 35693-99-3 | Clc1ccc(cc1c1cc(Cl)ccc1Cl)Cl | 6.04 | 5.61 | -2.49 |
| 60 | PCB 180 | 35065-29-3 | Clc1cc(Cl)c(cc1c1cc(Cl)c(c(c1Cl)Cl)Cl)Cl | 7.49 | 7.13 | -3.16 |

## **Table S12**. Training set for logK_pw_

| **S. No** | **Chemicals** | **CAS No** | **SMILES** | **logK_pw_** | **logK_ow_** | **logK_aw_** |
| --- | --- | --- | --- | --- | --- | --- |
| 1 | n-hexane | 110-54-3 | CCCCCC | 3.91 | 3.79 | 1.74 |
| 2 | n-octane | 111-65-9 | CCCCCCCC | 4.67 | 4.90 | 1.98 |
| 3 | 2,2,4-trimethylpentane | 540-84-1 | CC(CC(C)(C)C)C | 4.61 | 4.65 | 2.25 |
| 4 | cyclohexane | 110-82-7 | C1CCCCC1 | 3.27 | 3.51 | 1.12 |
| 5 | tetrachloromethane | 56-23-5 | ClC(Cl)(Cl)Cl | 2.61 | 2.80 | 0.33 |
| 6 | trichloroethene | 79-01-6 | ClC=C(Cl)Cl | 2.43 | 2.71 | -0.23 |
| 7 | di-n-butyl ether | 142-96-1 | CCCCOCCCC | 2.78 | 3.24 | -0.70 |
| 8 | di-n-pentyl ether | 693-65-2 | CCCCCOCCCCC | 3.77 | 4.33 | -0.44 |
| 9 | acetone | 67-64-1 | CC(=O)C | 0.06 | -0.30 | -2.81 |
| 10 | 2-octanone | 111-13-7 | CCCCCCC(=O)C | 2.42 | 2.47 | -2.15 |
| 11 | 2-nonanone | 821-55-6 | CCCCCCCC(=O)C | 2.83 | 3.01 | -2.02 |
| 12 | 2-decanone | 693-54-9 | CCCCCCCCC(=O)C | 3.16 | 3.57 | -1.91 |
| 13 | cyclohexanone | 108-94-1 | O=C1CCCCC1 | 0.54 | 0.90 | -3.54 |
| 14 | ethyl acetate | 141-78-6 | CCOC(=O)C | 0.46 | 0.71 | -2.09 |
| 15 | propyl acetate | 109-60-4 | CCCOC(=O)C | 1.01 | 1.29 | -1.93 |
| 16 | ethanol | 64-17-5 | CCO | -0.26 | -0.25 | -3.54 |
| 17 | 1-propanol | 71-23-8 | CCCO | 0.17 | 0.32 | -3.45 |
| 18 | 2-propanol | 67-63-0 | CC(O)C | -0.04 | 0.02 | -3.44 |
| 19 | 1-butanol | 71-36-3 | CCCCO | 0.51 | 0.91 | -3.36 |
| 20 | tert-butanol | 75-65-0 | CC(O)(C)C | 0.16 | 0.40 | -3.17 |
| 21 | 1-hexanol | 111-27-3 | CCCCCCO | 1.88 | 2.02 | -3.13 |
| 22 | 1-heptanol | 111-70-6 | CCCCCCCO | 2.38 | 2.57 | -3.01 |
| 23 | 4-heptanol | 589-55-9 | CCCC(CCC)O | 1.70 | 2.28 | -3.00 |
| 24 | 1-octanol | 111-87-5 | CCCCCCCCO | 2.66 | 3.13 | -2.89 |
| 25 | cyclopentanol | 96-41-3 | OC1CCCC1 | 0.52 | 0.82 | -4.04 |
| 26 | cycloheptanol | 502-41-0 | OC1CCCCCC1 | 1.51 | 1.93 | -3.98 |
| 27 | ethylene glycol | 107-21-1 | OCCO | -0.79 | -1.36 | -7.18 |
| 28 | 2-butoxyethanol | 111-76-2 | CCCCOCCO | 0.60 | 0.94 | -4.68 |
| 29 | benzyl alcohol | 100-51-6 | OCc1ccccc1 | 1.14 | 1.14 | -4.84 |
| 30 | p-xylene | 106-42-3 | Cc1ccc(cc1)C | 2.98 | 3.11 | -0.56 |
| 31 | chlorobenzene | 108-90-7 | Clc1ccccc1 | 2.91 | 2.78 | -0.71 |
| 32 | 1,2-dichlorobenzene | 95-50-1 | Clc1ccccc1Cl | 3.64 | 3.36 | -0.94 |
| 33 | 1,3-dichlorobenzene | 541-73-1 | Clc1cccc(c1)Cl | 3.71 | 3.45 | -0.68 |
| 34 | 1,4-dichlorobenzene | 106-46-7 | Clc1ccc(cc1)Cl | 3.57 | 3.44 | -0.74 |
| 35 | 1,2,3-trichlorobenzene | 87-61-6 | Clc1c(Cl)cccc1Cl | 4.19 | 4.07 | -1.03 |
| 36 | 1,2,4-trichlorobenzene | 120-82-1 | Clc1ccc(c(c1)Cl)Cl | 4.20 | 4.07 | -0.84 |
| 37 | 1,2,3,5-tetrachlorobenzene | 634-90-2 | Clc1cc(Cl)c(c(c1)Cl)Cl | 4.77 | 4.59 | -0.94 |
| 38 | 1,2,4,5-tetrachlorobenzene | 95-94-3 | Clc1cc(Cl)c(cc1Cl)Cl | 4.73 | 4.58 | -0.96 |
| 39 | pentachlorobenzene | 608-93-5 | Clc1cc(Cl)c(c(c1Cl)Cl)Cl | 5.18 | 5.08 | -1.33 |
| 40 | hexachlorobenzene | 118-74-1 | Clc1c(Cl)c(Cl)c(c(c1Cl)Cl)Cl | 5.64 | 5.62 | -1.31 |
| 41 | 2,4,5-trichlorotoluene | 6639-30-1 | Clc1cc(Cl)c(cc1C)Cl | 4.72 | 4.59 | -0.83 |
| 42 | 1,4-dibromobenzene | 106-37-6 | Brc1ccc(cc1)Br | 4.30 | 3.85 | -1.22 |
| 43 | 2 ,2',5,5'-tetrachlorobiphenyl (PCB 52) | 35693-99-3 | Clc1ccc(cc1c1cc(Cl)ccc1Cl)Cl | 5.94 | 5.61 | -2.49 |
| 44 | 2 ,2',4,5',6-pentachlorobiphenyl (PCB 103) | 60145-21-3 | Clc1ccc(c(c1)c1c(Cl)cc(cc1Cl)Cl)Cl | 6.32 | 5.91 | -2.48 |
| 45 | 2 ,2',4,6,6'-pentachlorobiphenyl (PCB 104) | 56558-16-8 | Clc1cc(Cl)c(c(c1)Cl)c1c(Cl)cccc1Cl | 6.13 | 5.83 | -2.40 |
| 46 | 2 ,2',3,3',6,6'-hexachlorobiphenyl (PCB 136) | 38411-22-2 | Clc1ccc(c(c1c1c(Cl)ccc(c1Cl)Cl)Cl)Cl | 6.50 | 6.39 | -2.67 |
| 47 | 2 ,2',3,4,4',5,6'-heptachlorobiphenyl (PCB 182) | 60145-23-5 | Clc1cc(Cl)c(c(c1)Cl)c1cc(Cl)c(c(c1Cl)Cl)Cl | 6.83 | 6.93 | -2.94 |
| 48 | phenanthrene | 85-01-8 | c1ccc2c(c1)c1ccccc1cc2 | 4.95 | 4.41 | -3.30 |
| 49 | pyrene | 129-00-0 | c1cc2ccc3c4c2c(c1)ccc4ccc3 | 5.71 | 4.58 | -4.51 |
| 50 | benzo[a]anthracene | 56-55-3 | c1ccc2c(c1)cc1c(c2)ccc2c1cccc2 | 6.44 | 5.62 | -4.83 |
| 51 | chrysene | 218-01-9 | c1ccc2c(c1)c1ccc3c(c1cc2)cccc3 | 6.40 | 5.60 | -4.91 |
| 52 | benzo[b]fluoranthene | 205-99-2 | c1ccc2c(c1)c1cc3ccccc3c3c1c2ccc3 | 7.11 | 6.10 | -5.73 |
| 53 | benzo[k]fluoranthene | 207-08-9 | c1ccc2c(c1)cc1c(c2)c2c3c1cccc3ccc2 | 7.13 | 6.21 | -5.56 |
| 54 | benzo[a]pyrene | 50-32-8 | c1ccc2c(c1)c1ccc3c4c1c(c2)ccc4ccc3 | 7.19 | 5.78 | -6.30 |
| 55 | benzo[ghi]perylene | 191-24-2 | c1cc2ccc3c4c2c(c1)c1cccc2c1c4c(cc3)cc2 | 7.78 | 6.83 | -6.59 |
| 56 | dibenz[a,h]anthracene | 53-70-3 | c1ccc2c(c1)c1cc3ccc4c(c3cc1cc2)cccc4 | 7.72 | 6.80 | -6.41 |
| 57 | indeno[1,2,3-cd]pyrene | 193-39-5 | c1ccc2c(c1)c1cc3cccc4c3c3c1c2ccc3cc4 | 7.86 | 6.65 | -6.23 |
| 58 | nitrobenzene | 98-95-3 | O=N(=O)c1ccccc1 | 2.01 | 1.91 | -2.99 |
| 59 | 2-nitrotoluene | 88-72-2 | O=N(=O)c1ccccc1C | 2.41 | 2.39 | -2.78 |
| 60 | quinoline | 91-22-5 | c1ccc2c(c1)nccc2 | 1.67 | 1.97 | -4.00 |
| 61 | diethylphthalate | 84-66-2 | CCOC(=O)c1ccccc1C(=O)OCC | 1.77 | 2.37 | -5.49 |
| 62 | dibutylphthalate | 84-74-2 | CCCCOC(=O)c1ccccc1C(=O)OCCCC | 3.87 | 4.58 | -4.83 |
| 63 | phenol | 108-95-2 | Oc1ccccc1 | 1.96 | 1.43 | -4.75 |
| 64 | 2-methylphenol | 95-48-7 | Cc1ccccc1O | 2.45 | 1.99 | -4.30 |
| 65 | 4-methylphenol | 106-44-5 | Cc1ccc(cc1)O | 2.35 | 1.97 | -4.60 |
| 66 | 4-ethylphenol | 123-07-9 | CCc1ccc(cc1)O | 2.78 | 2.31 | -4.62 |
| 67 | 2-n-propylphenol | 644-35-9 | CCCc1ccccc1O | 3.13 | 2.77 | -4.22 |
| 68 | 4-n-propylphenol | 645-56-7 | CCCc1ccc(cc1)O | 2.92 | 2.84 | -4.49 |
| 69 | 3,4,5-trimethylphenol | 527-54-8 | Oc1cc(C)c(c(c1)C)C | 2.66 | 2.76 | -5.01 |
| 70 | 4-n-butylphenol | 1638-22-8 | CCCCc1ccc(cc1)O | 3.13 | 3.38 | -4.35 |
| 71 | 2-sec-butylphenol | 89-72-5 | CCC(c1ccccc1O)C | 3.47 | 2.95 | -4.21 |
| 72 | 2-tert-butylphenol | 88-18-6 | Oc1ccccc1C(C)(C)C | 3.51 | 2.95 | -4.17 |
| 73 | 4-tert-butylphenol | 98-54-4 | CC(c1ccc(cc1)O)(C)C | 3.48 | 3.13 | -4.32 |
| 74 | 4-tert-amylphenol | 80-46-6 | CCC(c1ccc(cc1)O)(C)C | 3.54 | 3.62 | -4.30 |
| 75 | 2-phenylphenol | 90-43-7 | Oc1ccccc1c1ccccc1 | 3.43 | 3.02 | -6.69 |
| 76 | bisphenol A | 80-05-7 | CC(c1ccc(cc1)O)(c1ccc(cc1)O)C | 3.92 | 3.44 | -10.56 |
| 77 | 2-chlorophenol | 95-57-8 | Oc1ccccc1Cl | 2.76 | 1.93 | -3.63 |
| 78 | 3-chlorophenol | 108-43-0 | Oc1cccc(c1)Cl | 2.78 | 2.42 | -4.87 |
| 79 | 4-chlorophenol | 106-48-9 | Oc1ccc(cc1)Cl | 2.73 | 2.19 | -5.13 |
| 80 | 4-chloro-3-methylphenol | 59-50-7 | Oc1ccc(c(c1)C)Cl | 3.32 | 2.77 | -4.92 |
| 81 | 2,6-dichlorophenol | 87-65-0 | Clc1cccc(c1O)Cl | 2.86 | 2.82 | -3.68 |
| 82 | 2,4,5-trichlorophenol | 95-95-4 | Clc1cc(Cl)c(cc1O)Cl | 4.46 | 3.78 | -4.32 |
| 83 | 2,4,6-trichlorophenol | 88-06-2 | Clc1cc(Cl)c(c(c1)Cl)O | 3.80 | 3.76 | -4.10 |
| 84 | 3,4,5-trichlorophenol | 609-19-8 | Oc1cc(Cl)c(c(c1)Cl)Cl | 4.71 | 4.35 | -5.08 |
| 85 | 2,3,4,5-tetrachlorophenol | 4901-51-3 | Clc1c(O)cc(c(c1Cl)Cl)Cl | 4.76 | 4.30 | -4.26 |
| 86 | pentachlorophenol | 87-86-5 | Clc1c(O)c(Cl)c(c(c1Cl)Cl)Cl | 5.10 | 5.27 | -3.92 |
| 87 | 4-fluorophenol | 371-41-5 | Oc1ccc(cc1)F | 2.19 | 1.63 | -4.67 |
| 88 | 4-iodophenol | 540-38-5 | Oc1ccc(cc1)I | 2.55 | 2.66 | -5.41 |
| 89 | 3-nitrophenol | 554-84-7 | Oc1cccc(c1)N(=O)=O | 2.56 | 1.93 | -6.99 |
| 90 | 4-nitrophenol | 100-02-7 | Oc1ccc(cc1)N(=O)=O | 2.72 | 1.68 | -7.65 |
| 91 | 2,4-dinitrophenol | 51-28-5 | O=N(=O)c1ccc(c(c1)N(=O)=O)O | 2.67 | 1.57 | -5.56 |
| 92 | 2,6-dinitrophenol | 573-56-8 | Oc1c(cccc1N(=O)=O)N(=O)=O | 2.03 | 1.15 | -6.70 |
| 93 | 2-sec-butyl-4,6-dinitrophenol | 88-85-7 | CCC(c1cc(cc(c1O)N(=O)=O)N(=O)=O)C | 3.73 | 3.66 | -5.32 |
| 94 | 4-cyanophenol | 767-00-0 | N#Cc1ccc(cc1)O | 2.11 | 1.44 | -7.38 |
| 95 | aniline | 62-53-3 | Nc1ccccc1 | 1.63 | 1.18 | -4.16 |
| 96 | 3,4-dimethylaniline | 95-64-7 | Nc1ccc(c(c1)C)C | 2.11 | 2.08 | -4.18 |
| 97 | 3-nitroaniline | 99-09-2 | Nc1cccc(c1)N(=O)=O | 2.17 | 1.56 | -6.42 |
| 98 | N,N-dimethylaniline | 121-69-7 | CN(c1ccccc1)C | 2.33 | 2.37 | -2.65 |
| 99 | estrone | 53-16-7 | Oc1ccc2c(c1)CC[C@@H]1[C@@H]2CC[C@]2([C@H]1CCC2=O)C | 3.59 | 3.46 | -10.42 |
| 100 | estradiol | 50-28-2 | Oc1ccc2c(c1)CC[C@@H]1[C@@H]2CC[C@]2([C@H]1CC[C@@H]2O)C | 3.33 | 3.97 | -11.31 |
| 101 | estriol | 50-27-1 | Oc1ccc2c(c1)CC[C@@H]1[C@@H]2CC[C@]2([C@H]1C[C@H]([C@@H]2O)O)C | 1.96 | 1.50 | -17.17 |
| 102 | progesterone | 57-83-0 | O=C1CC[C@]2(C(=C1)CC[C@@H]1[C@@H]2CC[C@]2([C@H]1CC[C@@H]2C(=O)C)C)C | 3.28 | 3.27 | -10.87 |
| 103 | diazepam | 439-14-5 | Clc1ccc2c(c1)C(=NCC(=O)N2C)c1ccccc1 | 2.99 | 3.24 | -8.59 |
| 104 | lidocaine | 137-58-6 | CCN(CC(=O)Nc1c(C)cccc1C)CC | 2.15 | 2.57 | -7.52 |
| 105 | diclofenac | 15307-86-5 | OC(=O)Cc1ccccc1Nc1c(Cl)cccc1Cl | 4.45 | 4.60 | -9.14 |
| 106 | ibuprofen | 15687-27-1 | CC(Cc1ccc(cc1)C(C(=O)O)C)C | 3.80 | 3.90 | -5.68 |
| 107 | salicylic acid | 69-72-7 | OC(=O)c1ccccc1O | 2.55 | 2.16 | -5.40 |

## **Table S13**. Validation set for logK_pw_

| **S. No** | **Chemicals** | **CAS No** | **SMILES** | **logK_pw_** | **logK_ow_** | **logK_aw_** |
| --- | --- | --- | --- | --- | --- | --- |
| 1 | n-heptane | 142-82-5 | CCCCCCC | 4.55 | 4.34 | 1.86 |
| 2 | tetrachloroethene | 127-18-4 | ClC(=C(Cl)Cl)Cl | 3.08 | 3.28 | 0.09 |
| 3 | tribromomethane | 75-25-2 | BrC(Br)Br | 2.33 | 2.64 | -1.50 |
| 4 | cyclopentanone | 120-92-3 | O=C1CCCC1 | 0.30 | 0.45 | -3.43 |
| 5 | methanol | 67-56-1 | CO | -0.53 | -0.82 | -3.87 |
| 6 | 1-pentanol | 71-41-0 | CCCCCO | 1.08 | 1.46 | -3.24 |
| 7 | 3-pentanol | 584-02-1 | CCC(CC)O | 1.00 | 1.17 | -3.24 |
| 8 | cyclohexanol | 108-93-0 | OC1CCCCC1 | 1.01 | 1.35 | -3.98 |
| 9 | 1,3,5-trichlorobenzene | 108-70-3 | Clc1cc(Cl)cc(c1)Cl | 4.16 | 4.09 | -0.58 |
| 10 | 2 ,2',4,6-tetrachlorobiphenyl (PCB 50) | 62796-65-0 | Clc1cc(Cl)c(c(c1)Cl)c1ccccc1Cl | 5.92 | 5.48 | -2.35 |
| 11 | 3 ,3',4,5-tetrachlorobiphenyl (PCB 78) | 70362-49-1 | Clc1cccc(c1)c1cc(Cl)c(c(c1)Cl)Cl | 6.53 | 6.19 | -2.64 |
| 12 | anthracene | 120-12-7 | c1ccc2c(c1)cc1c(c2)cccc1 | 5.21 | 4.24 | -3.47 |
| 13 | fluoranthene | 206-44-0 | c1ccc2c(c1)c1cccc3c1c2ccc3 | 5.58 | 4.94 | -3.98 |
| 14 | dibenz[a,c]anthracene | 215-58-7 | c1ccc2c(c1)cc1c(c2)c2ccccc2c2c1cccc2 | 7.49 | 6.97 | -6.20 |
| 15 | 3-methylphenol | 108-39-4 | Cc1cccc(c1)O | 2.34 | 1.88 | -4.71 |
| 16 | 2-ethylphenol | 90-00-6 | CCc1ccccc1O | 2.81 | 2.32 | -4.38 |
| 17 | 2,6-dimethylphenol | 576-26-1 | Cc1cccc(c1O)C | 2.47 | 2.37 | -3.93 |
| 18 | 4-isopropylphenol | 99-89-8 | CC(c1ccc(cc1)O)C | 3.25 | 2.70 | -4.46 |
| 19 | 4-phenylphenol | 92-69-3 | Oc1ccc(cc1)c1ccccc1 | 3.52 | 3.06 | -6.54 |
| 20 | 2,4-dichlorophenol | 120-83-2 | Clc1ccc(c(c1)Cl)O | 3.57 | 3.06 | -3.67 |
| 21 | 3,4-dichlorophenol | 95-77-2 | Oc1ccc(c(c1)Cl)Cl | 3.76 | 3.43 | -4.75 |
| 22 | 4-bromophenol | 106-41-2 | Oc1ccc(cc1)Br | 2.40 | 2.38 | -5.31 |
| 23 | 2-nitrophenol | 88-75-5 | O=N(=O)c1ccccc1O | 1.89 | 1.91 | -3.43 |
| 24 | 3,4-dinitrophenol | 577-71-9 | Oc1ccc(c(c1)N(=O)=O)N(=O)=O | 3.17 | 1.68 | -9.02 |

## **Table S14**. Test set used to predict logK_lw_ with their experimental values of logK_tw_ and predicted values of logK_lw_ from tp-LFER model and the residuals

| **S. No** | **Chemicals** | **CAS No** | **SMILES** | **logK_tw_** | **tp-LFER predicted** | **Residuals** |
| --- | --- | --- | --- | --- | --- | --- |
| 1 | 1-ethyl-2-methylbenzene | 611-14-3 | CCC1=CC=CC=C1C | 3.83 | 3.63 | -0.20 |
| 2 | 1,2,4,5-tetramethylbenzene | 95-93-2 | Cc1cc(C)c(cc1C)C | 4.34 | 4.16 | -0.18 |
| 3 | Hexamethylbenzene | 87-85-4 | Cc1c(C)c(C)c(c(c1C)C)C | 4.89 | 5.31 | 0.42 |
| 4 | Iodobenzene | 591-50-4 | Ic1ccccc1 | 3.58 | 3.26 | -0.32 |
| 5 | 1,4-dichlorobenzene | 106-46-7 | Clc1ccc(cc1)Cl | 3.61 | 3.50 | -0.11 |
| 6 | 1,2,3-trichlorobenzene | 87-61-6 | Clc1c(Cl)cccc1Cl | 4.18 | 4.15 | -0.03 |
| 7 | 1,2,3,4-tetrachlorobenzene | 634-66-2 | Clc1c(Cl)ccc(c1Cl)Cl | 4.73 | 4.74 | 0.01 |
| 8 | Pentachloro benzene | 608-93-5 | Clc1cc(Cl)c(c(c1Cl)Cl)Cl | 5.32 | 5.37 | 0.05 |
| 9 | Hexachlorobenzene | 118-74-1 | Clc1c(Cl)c(Cl)c(c(c1Cl)Cl)Cl | 5.75 | 6.01 | 0.26 |
| 10 | Trichloroethylene | 79-01-6 | ClC=C(Cl)Cl | 2.71 | 2.43 | -0.28 |
| 11 | Lindane | 58-89-9 | Cl[C@@H]1[C@H](Cl)[C@@H](Cl)[C@@H]([C@@H]([C@H]1Cl)Cl)Cl | 3.91 | 4.07 | 0.16 |
| 12 | Dieldrin | 60-57-1 | ClC1=C(Cl)[C@@]2(C([C@@]1(Cl)[C@H]1[C@@H]2[C@H]2C[C@@H]1[C@H]1[C@@H]2O1)(Cl)Cl)Cl | 5.33 | 5.36 | 0.03 |
| 13 | Chlordane | 5103-74-2 | C1C2C(C(C1Cl)Cl)C3(C(=C(C2(C3(Cl)Cl)Cl)Cl)Cl)Cl | 5.73 | 6.43 | 0.70 |
| 14 | Heptachlor | 76-44-8 | ClC1C=CC2C1C1(Cl)C(=C(C2(C1(Cl)Cl)Cl)Cl)Cl | 6.00 | 5.67 | -0.33 |
| 15 | p,p′ -DDE | 72-55-9 | c1cc(ccc1C(=C(Cl)Cl)c2ccc(cc2)Cl)Cl | 5.99 | 6.75 | 0.76 |
| 16 | Indane | 496-11-7 | C1Cc2c(C1)cccc2 | 3.57 | 3.13 | -0.44 |
| 17 | 2,6-dimethylnaphthalene | 581-42-0 | Cc1ccc2c(c1)ccc(c2)C | 4.52 | 4.42 | -0.10 |
| 18 | 1,5-dimethylnaphthalene | 571-61-9 | Cc1cccc2c1cccc2C | 4.51 | 4.47 | -0.04 |

## **Table S15**. Test set used to predict logK_pw_ with their experimental values of logK_lipw_ and predicted values of logK_pw_ from tp-LFER model and the residuals

| **S. No** | **Compounds** | **CAS-RN** | **SMILES** | **logKlipw** | **tp-LFER predicted** | **Residuals** |
| --- | --- | --- | --- | --- | --- | --- |
| 1 | 2,4,6-trimethylaniline | 88-05-1 | Cc1cc(C)c(c(c1)C)N | 4.38 | 2.86 | -1.52 |
| 2 | 4-phenylbutylamine | 13214-66-9 | NCCCCc1ccccc1 | 2.41 | 2.53 | 0.12 |
| 3 | amlodipine | 88150-42-9 | NCCOCC1=C(C(=O)OCC)C(C(=C(N1)C)C(=O)OC)c1ccccc1Cl | 3.75 | 3.70 | -0.05 |
| 4 | chlorpromazine | 50-53-3 | CN(CCCN1c2ccccc2Sc2c1cc(Cl)cc2)C | 5.10 | 5.92 | 0.82 |
| 5 | hydroxyzine | 68-88-2 | OCCOCCN1CCN(CC1)C(c1ccc(cc1)Cl)c1ccccc1 | 3.40 | 3.01 | -0.39 |
| 6 | p-methylbenzyl-propylamine | 39190-96-0 | CCCNCc1ccc(cc1)C | 3.07 | 3.12 | 0.05 |
| 7 | procaine | 59-46-1 | CCN(CCOC(=O)c1ccc(cc1)N)CC | 2.38 | 2.50 | 0.12 |
| 8 | propranolol | 525-66-6 | OC(COc1cccc2c1cccc2)CNC(C)C | 3.24 | 3.99 | 0.75 |
| 9 | quinine | 130-95-0 | C=C[C@H]1CN2CC[C@H]1C[C@H]2[C@@H](c1ccnc2c1cc(OC)cc2)O | 2.73 | 4.10 | 1.37 |
| 10 | tetracaine | 94-24-6 | CCCCNc1ccc(cc1)C(=O)OCCN(C)C | 3.23 | 3.94 | 0.71 |
| 11 | 2,3,4,6-tetrachlorophenol | 58-90-2 | Oc1c(Cl)cc(c(c1Cl)Cl)Cl | 4.46 | 4.68 | 0.22 |
| 12 | 2,4,5-trichloro-phenol | 95-95-4 | Clc1cc(Cl)c(cc1O)Cl | 4.46 | 3.94 | -0.52 |
| 13 | 2,4-dichloro-phenol | 120-83-2 | Clc1ccc(c(c1)Cl)O | 2.87 | 3.21 | 0.34 |
| 14 | 2,4-dichloro-phenoxyacetic acid | 94-75-7 | OC(=O)COc1ccc(cc1Cl)Cl | 3.60 | 3.05 | -0.55 |
| 15 | 2,6-dichloro-phenol | 87-65-0 | Clc1cccc(c1O)Cl | 2.87 | 2.89 | 0.02 |
| 16 | 2-methyl-4,6- dinitrophenol | 534-52-1 | O=N(=O)c1cc(C)c(c(c1)N(=O)=O)O | 2.76 | 2.25 | -0.51 |
| 17 | 2-s-butyl-4,6- dinitrophenol | 88-85-7 | CCC(c1cc(cc(c1O)N(=O)=O)N(=O)=O)C | 3.96 | 3.80 | -0.16 |
| 18 | 2-tert-butyl-4,6-dinitrophenol | 1420-07-1 | O=N(=O)c1cc(N(=O)=O)c(c(c1)C(C)(C)C)O | 4.10 | 3.92 | -0.18 |
| 19 | 3,5-dibromo-4- hydroxy-benzonitrile | 1689-84-5 | N#Cc1cc(Br)c(c(c1)Br)O | 3.16 | 3.79 | 0.63 |
| 20 | 3,5-dibromo-4-methylphenol | 13979-81-2 | Oc1cc(Br)c(c(c1)Br)C | 4.51 | 4.13 | -0.38 |
| 21 | 3,5-dichlorophenol | 591-35-5 | Oc1cc(Cl)cc(c1)Cl | 3.76 | 3.87 | 0.11 |
| 22 | 4,5,6,7-tetrachloro- 2-(trifluoromethyl)-1H-benzimidazole | 2338-29-6 | Clc1c(Cl)c(Cl)c(c2c1nc([nH]2)C(F)(F)F)Cl | 4.35 | 5.08 | 0.73 |
| 23 | 4-methyl-2,6- dinitrophenol | 609-93-8 | Cc1cc(N(=O)=O)c(c(c1)N(=O)=O)O | 2.34 | 2.47 | 0.13 |
| 24 | 4-tert-butyl-2,6- dinitrophenol | 4097-49-8 | O=N(=O)c1cc(cc(c1O)N(=O)=O)C(C)(C)C | 3.81 | 3.92 | 0.11 |
| 25 | 5,6-dichloro-2- (trifluoromethyl)-benzimidazole | 2338-25-2 | Clc1cc2[nH]c(nc2cc1Cl)C(F)(F)F | 3.05 | 3.69 | 0.64 |
| 26 | 5,7-dibromo-8-hydroxyquinoline | 521-74-4 | Brc1cc(Br)c2c(c1O)nccc2 | 3.94 | 3.89 | -0.05 |
| 27 | 5,7-dichloro-8-hydroxyquinoline | 773-76-2 | Clc1cc(Cl)c2c(c1O)nccc2 | 3.35 | 3.34 | -0.01 |
| 28 | 5-chloro-8-hydroxyquinoline | 130-16-5 | Clc1ccc(c2c1cccn2)O | 3.29 | 3.26 | -0.03 |
| 29 | 5-phenylvaleric acid | 2270-20-4 | OC(=O)CCCCc1ccccc1 | 3.06 | 3.17 | 0.11 |
| 30 | 8-hydroxy-quinoline | 148-24-3 | Oc1cccc2c1nccc2 | 2.17 | 1.97 | -0.20 |
| 31 | carbonyl cyanide m- chlorophenyl-hydrazone | 555-60-2 | N#CC(=NNc1cccc(c1)Cl)C#N | 4.05 | 3.70 | -0.35 |
| 32 | carbonyl cyanide p- methoxyphenylhydrazone | 370-86-5 | N#CC(=NNc1ccc(cc1)OC(F)(F)F)C#N | 4.22 | 4.03 | -0.19 |
| 33 | octanoic acid | 124-07-2 | CCCCCCCC(=O)O | 2.91 | 3.24 | 0.33 |
| 34 | pentachloro-phenol | 87-86-5 | Clc1c(O)c(Cl)c(c(c1Cl)Cl)Cl | 5.10 | 5.52 | 0.42 |
| 35 | tetrachloro-catechol | 1198-55-6 | Oc1c(O)c(Cl)c(c(c1Cl)Cl)Cl | 4.41 | 4.79 | 0.38 |
| 36 | Warfarin | 81-81-2 | CC(=O)C[C@@H](c1c(=O)oc2c(c1O)cccc2)c1ccccc1 | 1.40 | 2.99 | 1.59 |


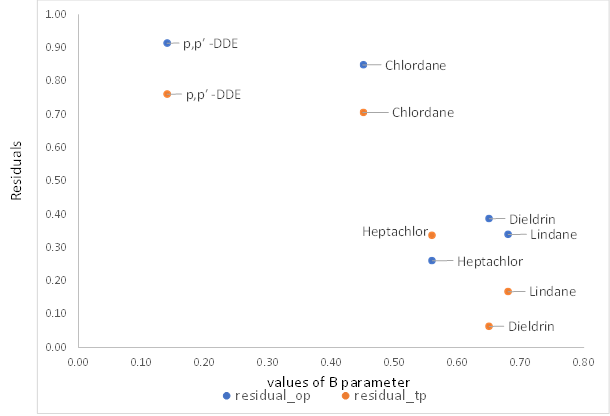


## **Figure S1**. The values of absolute residuals for op-LFER and tp-LFER as a function of Abraham solute parameter B, for organochlorine pesticides.

**Regression diagnostic of all developed models**

There are number of diagnostic elements which primarily highlight the influential values or outliers in the data set of dependent and independent variables these diagnostic helps to make model robust and effective for predictive purpose, so we can remove any existing outlier easily.

##

## **Section 1**. Cross Validation of tp-LFER Models

In this section, we reported the results of four Cross-validation tests such as leave-one-out, k-fold (k=10, repeat=0 and 3), and bootstrapping with 1000 resamples. These tests were performed on training and validation sets to assess the internal validation, robustness, and predictive capability of each model.

## **Table S16.** Cross-Validation Test for tp-LFER model of logK_lw_ (n=305)

| Test | RMSE | R^2^ | MAE |
| --- | --- | --- | --- |
| Leave-One-Out Cross-Validation | 0.377 | 0.970 | 0.282 |
| 10-fold cross-validation | 0.369 | 0.971 | 0.280 |
| 3 × Repeated 10-fold cross-validation | 0.372 | 0.971 | 0.282 |
| Bootstrapped resampling (1000 reps) | 0.378 | 0.970 | 0.284 |

## **Table S17.** Cross-Validation Test for tp-LFER model of logK_pw_ (n=131)

| Test | RMSE | R^2^ | MAE |
| --- | --- | --- | --- |
| Leave-One-Out Cross-Validation | 0.420 | 0.950 | 0.332 |
| 10-fold cross-validation | 0.418 | 0.948 | 0.334 |
| 3 × Repeated 10-fold cross-validation | 0.412 | 0.951 | 0.332 |
| Bootstrapped resampling (1000 reps) | 0.422 | 0.951 | 0.335 |

## **Section 2**. Dimensionality analysis of all models


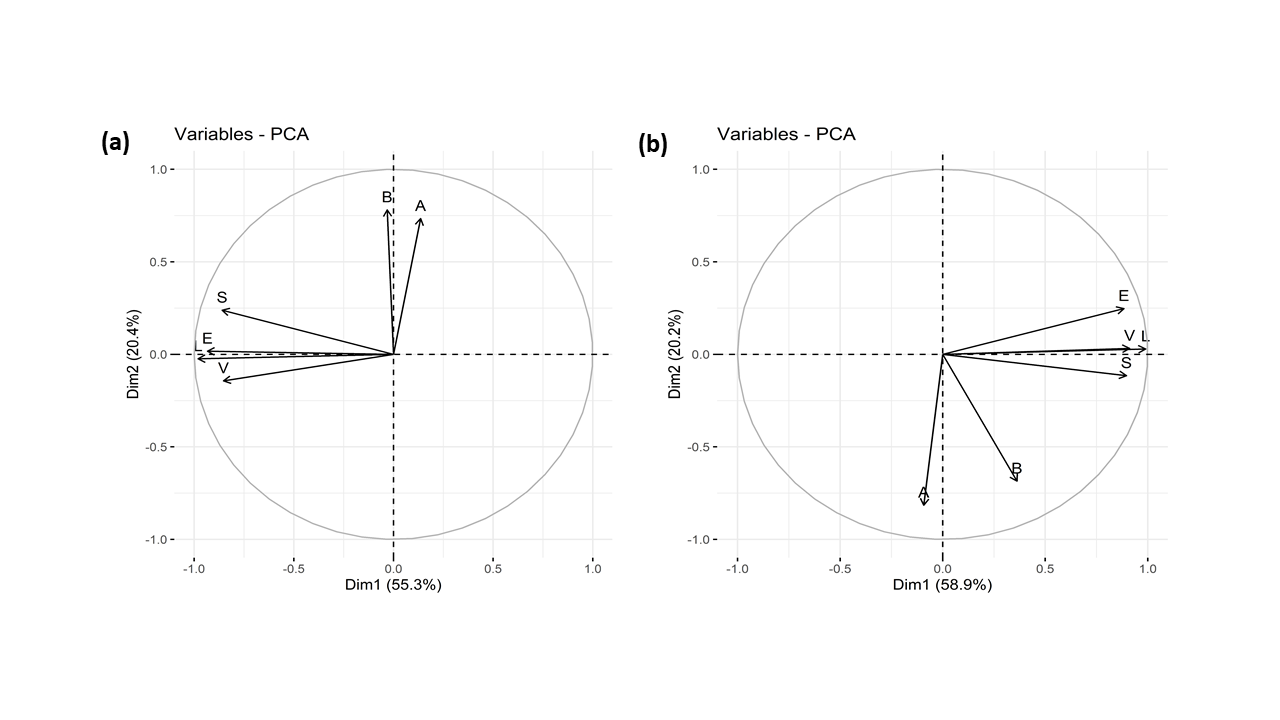


## **Figure S2**. PCA plot on Abraham solute descriptors for (a) logK_lw_ and (b) logK_pw_ tp-LFER models


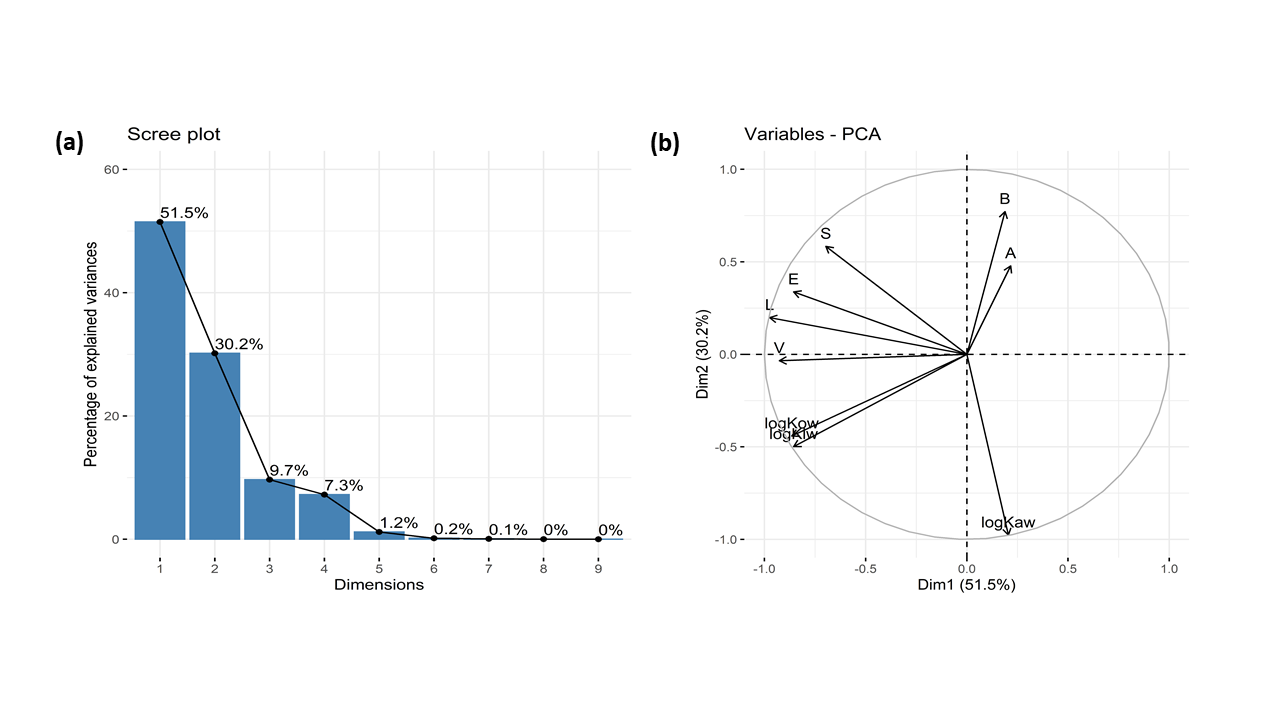


**Figure S3**. Scree plot (a) and correlation circle (b) of logKlw tp-LFER model


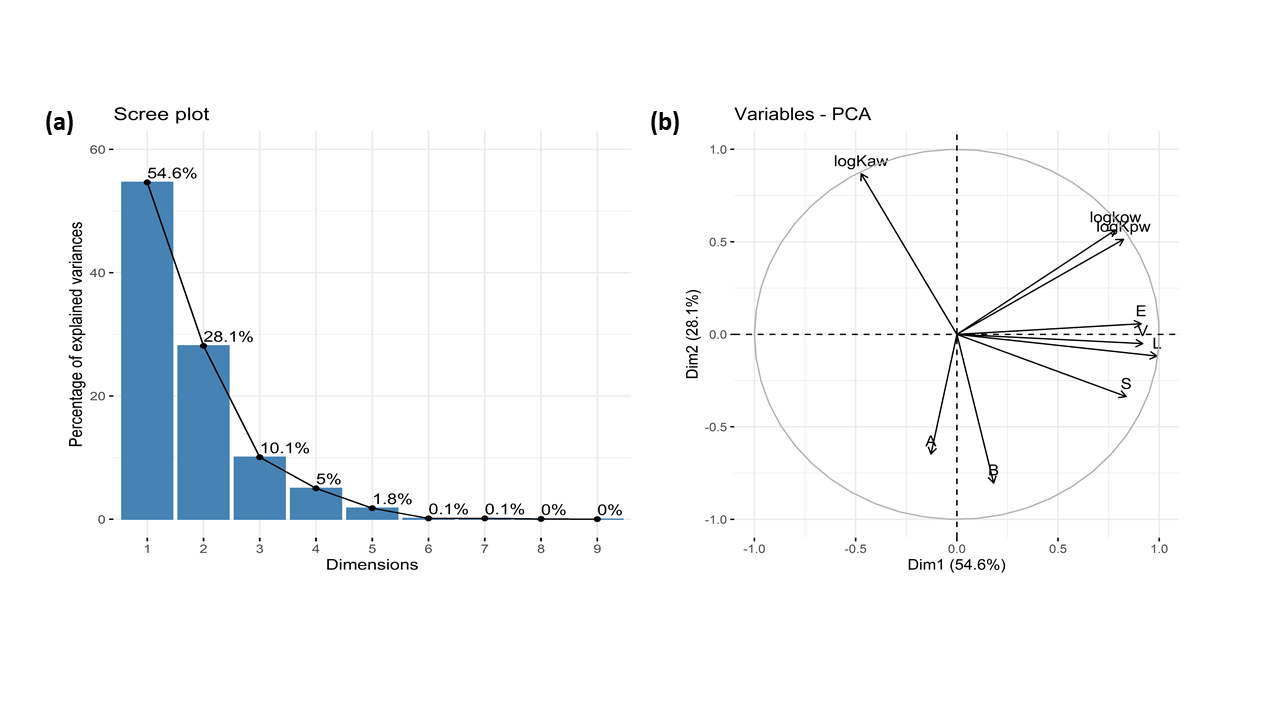


## **Figure S4**. Scree plot (a) and correlation circle (b) of logK_pw_tp-LFER model

## **Section 3**. Application domain of all models (flagged chemicals)

In this section, we will present the influential observations of the developed models.

## **Table S18.** List of flagged chemicals for logK_lw_ tp-LFER model

| **Chemical** | **CAS No** | **StudRes** | **HAT value** | **Cook’D** | **logK_lw_** | **logK_ow_** | **logK_aw_** |
| --- | --- | --- | --- | --- | --- | --- | --- |
| 2,2,3,3,4,4,4-Heptafluoro-1-butanol | 375-01-9 | -3.304 | 0.003 | 0.011 | 1.17 | 2.48 | -1.60 |
| pentadecane | 629-62-9 | -0.871 | 0.042 | 0.011 | 9.31 | 8.80 | 2.80 |
| 2,4-Dinitrotoluene | 121-14-2 | 3.742 | 0.018 | 0.082 | 2.34 | 1.42 | -5.88 |
| hexadecane | 544-76-3 | -0.989 | 0.049 | 0.016 | 9.88 | 9.35 | 2.91 |
| Benzo[a]pyrene | 50-32-8 | 2.873 | 0.038 | 0.108 | 6.79 | 5.78 | -6.30 |

## **Table S19.** List of flagged chemicals for logK_pw_ tp-LFER model

| **Chemical** | **CAS No** | **StudRes** | **HAT value** | **Cook’D** | **logK_pw_** | **logK_ow_** | **logK_aw_** |
| --- | --- | --- | --- | --- | --- | --- | --- |
| 3,4-dinitrophenol | 577-71-9 | 2.828 | 0.0359 | 0.094 | 3.17 | 1.68 | -9.02 |
| -estradiol | 50-28-2 | -3.384 | 0.066 | 0.251 | 3.33 | 3.97 | -11.31 |
| estriol | 50-27-1 | -0.938 | 0.182 | 0.065 | 1.96 | 1.50 | -17.17 |

## **Section 4.** One parameter LFER (op-LFER) equations for all models

1. **Storage lipid-water op-LFER model equation on complete dataset**

$${logK}_{lw}= -0.387+1.120{logK}_{ow}$$

n = 305, R^2^ = 0.967, Adj. R^2^ = 0.966, rmse = 0.399, F statistics = 8879

1. **Storage lipid-water op-LFER model equation for polar chemicals only**

$${logK}_{lw}\mathbf{= -}0.603+1.063{logK}_{ow}$$

n = 89, R^2^ = 0.823, Adj. R^2^ = 0.821, rmse = 0.510, F statistics = 405

1. **Storage lipid-water tp-LFER model equation for polar chemicals only**

$${logK}_{lw}\mathbf{= -}0.143+1.003{logK}_{ow}+0.151{logK}_{aw}$$

n = 89, R^2^ = 0.878, Adj. R^2^ = 0.875, rmse = 0.426, F statistics = 310

1. **Phospholipid-water op-LFER model equation on complete dataset**

$${logK}_{pw}\mathbf{=}0.006+1.059{logK}_{ow}$$

n = 131, R^2^ = 0.946, Adj. R^2^ = 0.945, rmse = 0.440, F statistics = 2269

## **Section 5.** Test sets

1. **Storage lipid-water (logK_lw_) test set**

The test set to predict logK_lw_ was taken from the literature^4^. Here, ultra-pure triolein was considered as model lipid. 53 compounds were measured for triolein-water partition coefficient. Among these, we found 18 chemicals (Table S10 in SM) which were not present in our main dataset (n=305, Table S1 in SM). The experimental values of logK_ow_ and logK_aw_ for these chemicals were obtained from EPI Suite software. Predictions against the experimental values of logK_tw_ were made using the eq 4 of the main manuscript developed to estimate logK_lw_. Residuals were calculated from predicted and experimental values and finally their root mean square error was calculated.

**b. Phospholipid-water (logK_pw_) test set**

The test set to predict logK_pw_ was taken from the literature^5–11^. Here, phospholipid-water partition coefficient was measured for neutral organic chemicals. Among them, 36 chemicals (Table S11 in SM) were selected which were not present in our main dataset (n=131, Table S2 in SM) on which tp-LFER model has been developed to estimate logK_pw_. The experimental values of logK_ow_ and logK_aw_ for these chemicals were obtained from EPI Suite software. Then we predicted the values of logK_pw_ using the eq 6 of the main manuscript. Finally, the residuals and root mean square error were calculated.

## References

1. Li, M. *et al.* Developing the QSPR model for predicting the storage lipid/water distribution coefficient of organic compounds. *Front. Environ. Sci. Eng.* **15**, (2021).

2. Geisler, A., Oemisch, L., Endo, S. & Goss, K. U. Predicting storage-lipid water partitioning of organic solutes from molecular structure. *Environ. Sci. Technol.* **49**, 5538–5545 (2015).

3. Endo, S., Escher, B. I. & Goss, K. U. Capacities of membrane lipids to accumulate neutral organic chemicals. *Environ. Sci. Technol.* **45**, 5912–5921 (2011).

4. Goss, K. U. Predicting the equilibrium partitioning of organic compounds using just one linear solvation energy relationship (LSER). *Fluid Phase Equilib.* **233**, 19–22 (2005).

5. Poole, C. F., Ariyasena, T. C. & Lenca, N. Estimation of the environmental properties of compounds from chromatographic measurements and the solvation parameter model. *J. Chromatogr. A* **1317**, 85–104 (2013).

6. Klamt, A. Prediction of Phospholipid − Water Partition Coe ffi cients of Ionic Organic Chemicals Using the Mechanistic Model COSMO mic. (2014).

7. Barzanti, C. *et al.* Potentiometric determination of octanol-water and liposome-water partition coefficients (log P) of ionizable organic compounds. *Tetrahedron Lett.* **48**, 3337–3341 (2007).

8. Escher, B. I., Schwarzenbach, R. P. & Westall, J. C. Evaluation of liposome - Water partitioning of organic acids bases. 2. Comparison of experimental determination methods. *Environ. Sci. Technol.* **34**, 3962–3968 (2000).

9. Hung, W. N., Chiou, C. T. & Lin, T. F. Lipid-water partition coefficients and correlations with uptakes by algae of organic compounds. *J. Hazard. Mater.* **279**, 197–202 (2014).

10. Ripley, B. D. R. Development Core Team R: A Language and Environmental for Statistical Computing; R Foundation for Statistical Computing: Vienna, Austria. 1–3 (2011).

11. Bittermann, K., Spycher, S. & Goss, K. U. Comparison of different models predicting the phospholipid-membrane water partition coefficients of charged compounds. *Chemosphere* **144**, 382–391 (2016).
